# Rational design and synthesis of furan-based Schiff base quinazolin-4-amine hybrids: spectral elucidation, molecular docking, DFT studies, and *in silico* ADMET-antioxidant study

**DOI:** 10.1039/d6ra08647k

**Published:** 2026-10-20

**Authors:** Sunil D. Narode, Miral Vora, Jay B. Maheta, Yogesh O. Bhola, Darshna K. Lakhnotra, Nikita J. Patel

**Affiliations:** a Department of Chemistry, Krishna School of Science, Drs. Kiran & Pallavi Patel Global University (KPGU) Vadodara Gujarat India patelmiral902@gmail.com; b Department of Chemistry, Shri M. P. Pandya Science College, Shri Govind Guru University Lunawada, Mahisagar Godhra Gujarat India

## Abstract

In this work, a new series of furan-based quinazoline-4-amine hydrazone (Schiff base) derivatives (4a–h) was rationally designed and synthesized in good yields (84–90%) through a multistep route involving nucleophilic aromatic substitution, Suzuki–Miyaura cross-coupling, and hydrazone/Schiff base condensation reactions. The synthesized compounds were characterized by FT-IR, ^1^H NMR, ^13^C NMR, and ESI-mass spectrometry, which confirmed the proposed structures. Density functional theory (DFT) calculations were performed to optimize the molecular geometries and to derive frontier molecular orbital energies, global reactivity descriptors, Mulliken atomic charges, and molecular electrostatic potential (MEP) surfaces, providing insight into the stability, reactivity, and radical-scavenging propensity of the compounds. Molecular docking studies against the Kelch domain of Keap1 (PDB 4L7B), the principal negative regulator of the Nrf2-mediated antioxidant response, were carried out using the Schrödinger Suite (Glide, Maestro interface) to explore, *in silico*, a possible interaction of the derivatives with this target; the docked compounds gave Glide docking scores of −7.325 to −8.603 kcal mol; these results constitute an *in silico* hypothesis of Keap1 binding that remains to be tested experimentally. Computational ADMET profiling indicated drug-like physicochemical properties overall, with the dimethoxy- and phenolic-substituted analogues 4e and 4h showing the most favourable predicted oral bioavailability (score = 0.55) among the series. The *in vitro* antioxidant potential of compounds 4a–h was evaluated using the DPPH radical-scavenging assay, giving IC_50_ values of 85.5 ± 3.7 to 148.1 ± 3.2 µM, compared with 235.2 ± 4.6 µM for the standard antioxidant ascorbic acid; on a molar basis, all eight derivatives were therefore more potent than ascorbic acid in this cell-free assay. Among the series, compounds 4d and 4f displayed the most promising radical-scavenging activity (IC_50_ = 85.5 and 90.8 µM, respectively), although the global DFT reactivity descriptors did not correlate with the measured activity across the series.

## Abstract 2

Furan-based quinazolin-4-amine hydrazone hybrids (4a–h) were synthesized in 84–90% yields. DFT, Keap1 docking and ADMET supported the scaffold. Compounds showed superior DPPH antioxidant activity to ascorbic acid, with 4d and 4f most active.

## Graphical abstract

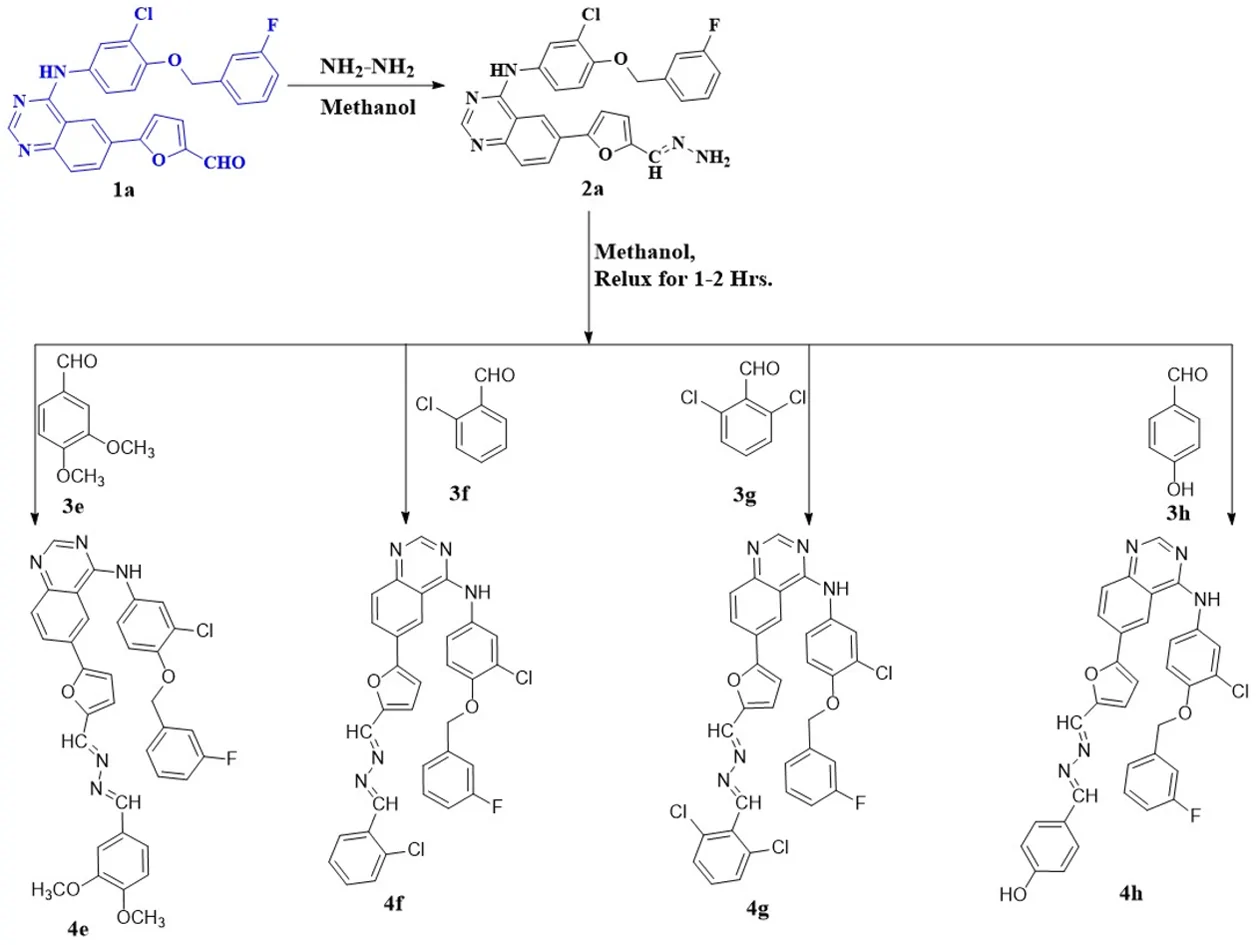

## 1. Introduction

Reactive oxygen species (ROS), including the superoxide anion, hydroxyl radical, and hydrogen peroxide, are continuously generated as by-products of normal aerobic metabolism. Under physiological conditions, their levels are tightly regulated by an intricate network of enzymatic (superoxide dismutase, catalase, glutathione peroxidase) and non-enzymatic antioxidants. However, an imbalance between ROS generation and the endogenous antioxidant defence system leads to a state of oxidative stress, which causes cumulative damage to lipids, proteins, and nucleic acids. Oxidative stress is now well recognized as a central pathological driver in a wide spectrum of chronic and degenerative disorders, including cancer, diabetes mellitus, cardiovascular disease, and neurodegenerative conditions such as Alzheimer's and Parkinson's disease. Consequently, molecules capable of scavenging free radicals and reinforcing the cellular antioxidant defence machinery, such as the Keap1-Nrf2-ARE signalling axis, are of substantial therapeutic interest, and the design of new, low-toxicity synthetic antioxidants remains an actively pursued goal in medicinal chemistry.

Among the many pathological conditions linked to chronic oxidative stress, cancer remains one of the leading causes of death worldwide,^1–6^ and its conventional treatment is limited by poor selectivity, severe side effects and multidrug resistance.^7,8^ Oxidative damage is likewise implicated in diabetic complications, atherosclerosis and neurodegeneration, so compounds able to neutralise free radicals are of broad interest. Small synthetic antioxidants usually act by hydrogen-atom transfer (HAT) or single-electron transfer (SET) to the radical, and their efficiency depends on the presence of labile N–H/O–H bonds and on the ability of a conjugated framework to delocalise the resulting radical. Extended π-conjugation, electron-rich heteroaromatic rings such as furan and thiophene, phenolic hydroxyl groups and hydrazone (–CH=N–N=CH–) linkers are therefore recurring features of antioxidant pharmacophores.

The logical construction of hybrid pharmacophores has become a potent tactic in contemporary drug discovery to address such multifactorial disorders. Several hybrid-based molecules are presently undergoing preclinical and clinical investigation for a range of therapeutic indications, as recent developments in medicinal chemistry have shown that such hybrid structures exhibit increased biological activity and therapeutic promise.^9,10^ The quinazoline motif has drawn special interest among the various heterocyclic scaffolds investigated in drug design due to its wide variety of pharmacological characteristics and as a bioisosteric substitute for carbonyl-containing functions including amides, carboxylic acids, and esters.^11,12^ Due to their widespread and unique biological actions, quinazoline derivatives, which are classified as N-containing heterocyclic compounds, have attracted worldwide attention. Numerous therapeutic activities of quinazoline derivatives have already been identified by researchers, such as anti-cancer,^13–15^ anti-inflammation,^16–18^ anti-bacterial,^19–21^ analgesia,^22–24^ anti-tuberculosis,^25,26^ anti-oxidation,^27–29^ anti-malarial,^30,31^ anti-hypertension,^32,33^ anti-diabetes,^34,35^ and more. By adding different active groups to the quinazoline moiety utilizing evolving synthetic techniques, medicinal chemists created a range of quinazoline molecules with distinct biological activity.

Schiff base-derived scaffolds are very appealing building blocks for hybrid drug creation because the azomethine (C=N) linkage that characterizes Schiff bases provides molecular stiffness and enhanced interactions with biological macromolecules. Due to its capacity to modulate metal-dependent enzymatic activities and to stabilize free-radical intermediates, the quinazoline nucleus has also drawn special attention as an antioxidant pharmacophore. The goal of the current work is to maximize the antioxidant potential of quinazoline pharmacophores while minimizing toxicity by strategically utilizing the biological activity profile of the azomethine moiety through the use of a hydrazone intermediate as the essential Schiff base linker. An integrated computational–experimental strategy has been used to effectively and logically accomplish this goal. To determine the most stable molecular conformations, evaluate reactivity descriptors, and explore, as an *in silico* hypothesis, possible binding interactions of the proposed compounds with the Kelch domain of Keap1, the principal negative regulator of the Nrf2-mediated antioxidant response pathway, quantum chemical calculations (DFT) and molecular docking studies are first utilized. In order to determine the most promising candidates among the synthesized compounds, ADMET profiling, DFT-assisted study, molecular docking, and *in vitro* antioxidant activity were carried out to assess their drug-likeness and predicted pharmacokinetic properties. Computational chemistry offers profound insights into molecule reactivity, stability, and binding behaviour while drastically cutting experimental costs and time in the early stages of drug discovery. All eight designed compounds were synthesized, and their free-radical scavenging potential was evaluated by the *in vitro* DPPH radical-scavenging assay. In addition to improving lead identification efficiency, this hybrid computational–experimental approach fortifies the scientific basis for the logical development of potent, selective, and less toxic antioxidant agents.

## 2. Methodology

### 2.1 Reagents and instruments

All compounds utilized in synthesis, such as triethylamine, hydrazine hydrate, methanol, palladium acetate, substituted aldehydes, and others. We bought acetic acid from SRL, Loba Chemicals, and Merck. The compounds were utilized without any additional purification. Solvents were either distilled before use or of spectroscopic quality. All compounds were investigated using Fourier-transform infrared (FT-IR) in the solid state using a Cary630 FT-IR spectrometer. ^1^H NMR and ^13^C NMR spectrum data were collected on an ECZ400S 400 MHz spectrometer in CDCl_3_ and DMSO-*d*_6_. Coupling constant (*J*) values are in Hz. Melting points were determined by the open capillary method using a melting point apparatus and are uncorrected. Elemental analyses (C, H, N) were performed using a PerkinElmer 2400 Series II CHNS/O Elemental Analyzer, and ESI mass spectra were recorded in positive-ion mode using a Waters Xevo G2-XS QTOF high-resolution mass spectrometer.

### 2.2 General reaction procedure of synthesised furan-based quinazoline-4-amine derivative scaffolds

In the first step, 4-chloro-6-iodoquinazoline was reacted with 3-chloro-4-((3-fluorobenzyl)oxy)aniline in ethanol as a solvent. The reaction mixture was heated at 70–80 °C for 8–10 hours under continuous stirring. During this process, the nucleophilic amine group of the substituted aniline attacks the electron-deficient C-4 position of the quinazoline ring, leading to the displacement of chloro substituent *via* a nucleophilic aromatic substitution (SNAr) mechanism. After completion of the reaction (monitored by TLC), the mixture was cooled to room temperature, resulting in the precipitation of the product. The solid was collected by filtration, washed with cold ethanol to remove impurities, and dried under reduced pressure to afford the intermediate *N*-(3-chloro-4-((3-fluorobenzyl)oxy)phenyl)-6-iodoquinazolin-4-amine.

In the second step, the obtained iodoquinazoline intermediate was subjected to a Suzuki–Miyaura cross-coupling reaction with (5-formylfuran-2-yl)boronic acid (1.2 mmol). The reaction was carried out in a mixed solvent system of THF and methanol, which provides good solubility for both organic and inorganic components. To this mixture, triethylamine (2.5 mmol) was added as a base to facilitate the transmetalation step, along with a catalytic amount of palladium acetate (0.05 mmol) as the coupling catalyst. The reaction mixture was heated at 60–65 °C for 6 hours under stirring. In this step, the palladium catalyst undergoes oxidative addition with the aryl iodide, followed by transmetalation with the boronic acid and subsequent reductive elimination to form a new carbon carbon bond, introducing the furan-2-carbaldehyde moiety onto the quinazoline scaffold. The filtrate was then concentrated under reduced pressure to remove solvents, yielding the product as an aromatic aldehyde compound (1a), that is 5-(4-((3-chloro-4-((3-fluorobenzyl)oxy)phenyl)amino)quinazolin-6-yl)furan-2-carbaldehyde, in good yield (Scheme 1).

**Scheme 1:**
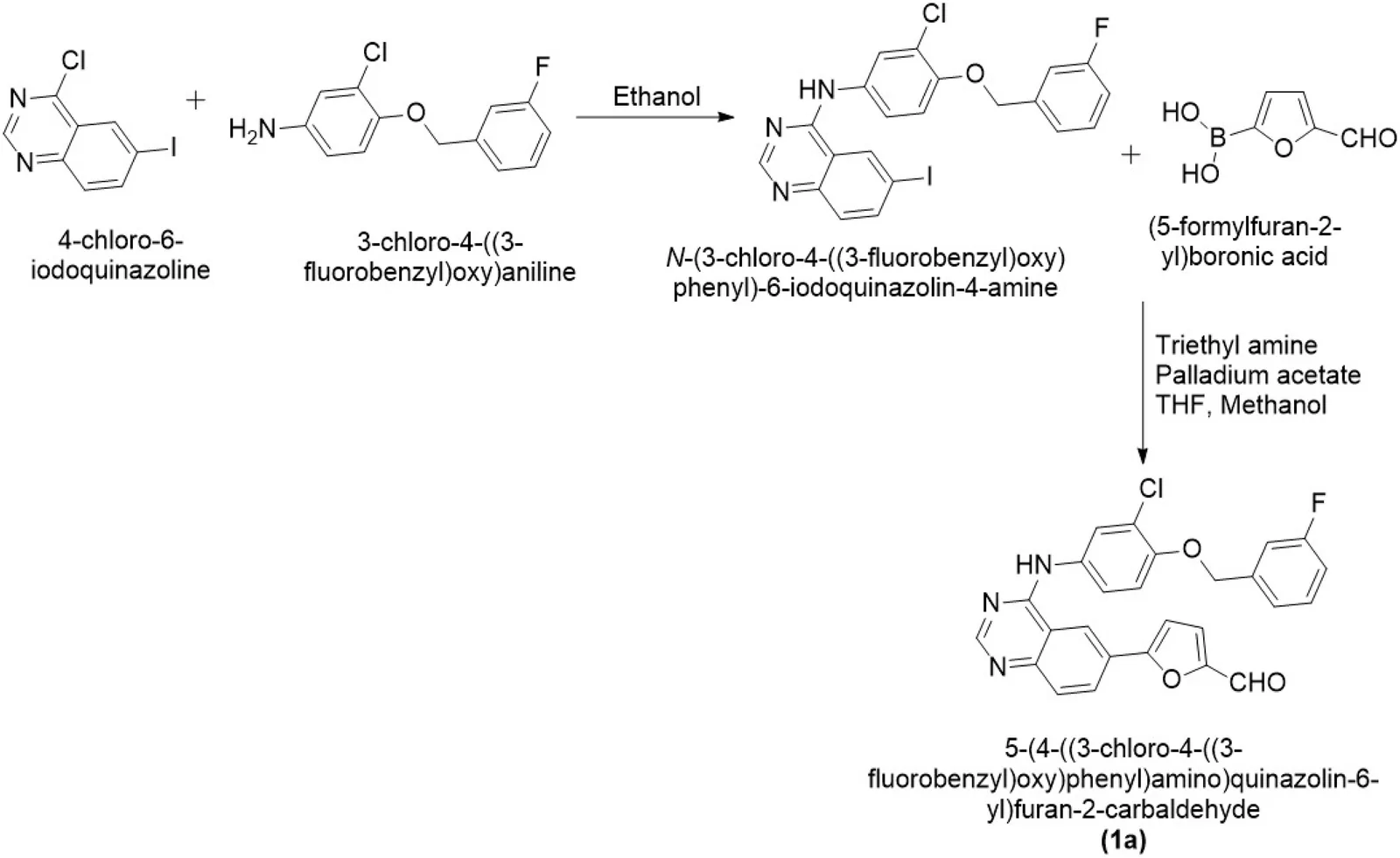
Synthesis of quinazolin-6-furan-2-carbaldehyde derivative compounds (1a).

After dissolving 5-(4-((3-chloro-4-((3-fluorobenzyl)oxy)phenyl)amino)quinazolin-6-yl)furan-2-carbaldehyde (1a) (0.01 mol) and hydrazine hydrate (0.01 mol) in 25–30 mL of methanol, the mixture was agitated at room temperature. The matching hydrazone intermediate (2a) was subsequently obtained by refluxing the reaction mixture for one to two hours. Thin-layer chromatography (TLC) was used to track the reaction's development using silica gel plates and a suitable solvent solution (ethyl acetate : *n*-hexane, 1 : 4 ratio). The precipitated product was filtered, cleaned with cold methanol, and vacuum-dried when the reaction mixture had cooled to room temperature. Without additional purification, the resulting intermediate was utilized for the subsequent stage (Scheme 2).

**Scheme 2:**
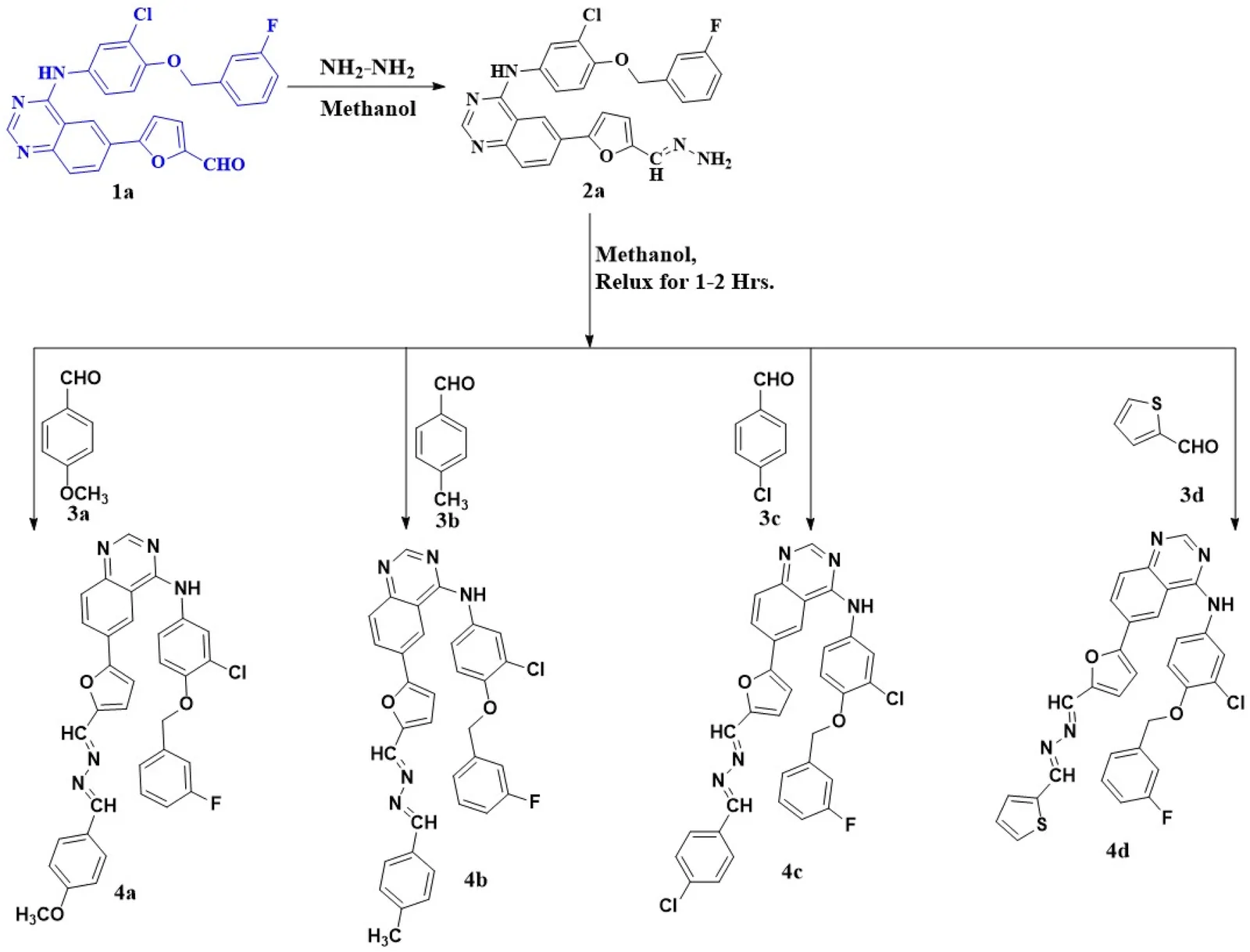
Reaction procedure of synthesised furan-based quinazoline-4-amine derivative scaffolds (4a–d).

Substituted aromatic/heteroaromatic aldehydes (3a–3h) (0.01 mol) were then added after intermediate 2a (0.01 mol) was dissolved in methanol (20–25 mL). The resultant reaction mixture was continuously stirred and refluxed in methanol for one to two hours. TLC analysis verified the reaction's completion. To get pure furan-based quinazoline hydrazone derivatives, the separated solid products (4a–h) were collected by filtering, extensively cleaned with cold methanol to remove impurities, and recrystallized from appropriate solvents after cooling to room temperature. FT-IR, ^1^H NMR, ^13^C NMR, and mass spectrometry were among the spectroscopic techniques used to characterise the products after drying (Scheme 3).

**Scheme 3:**
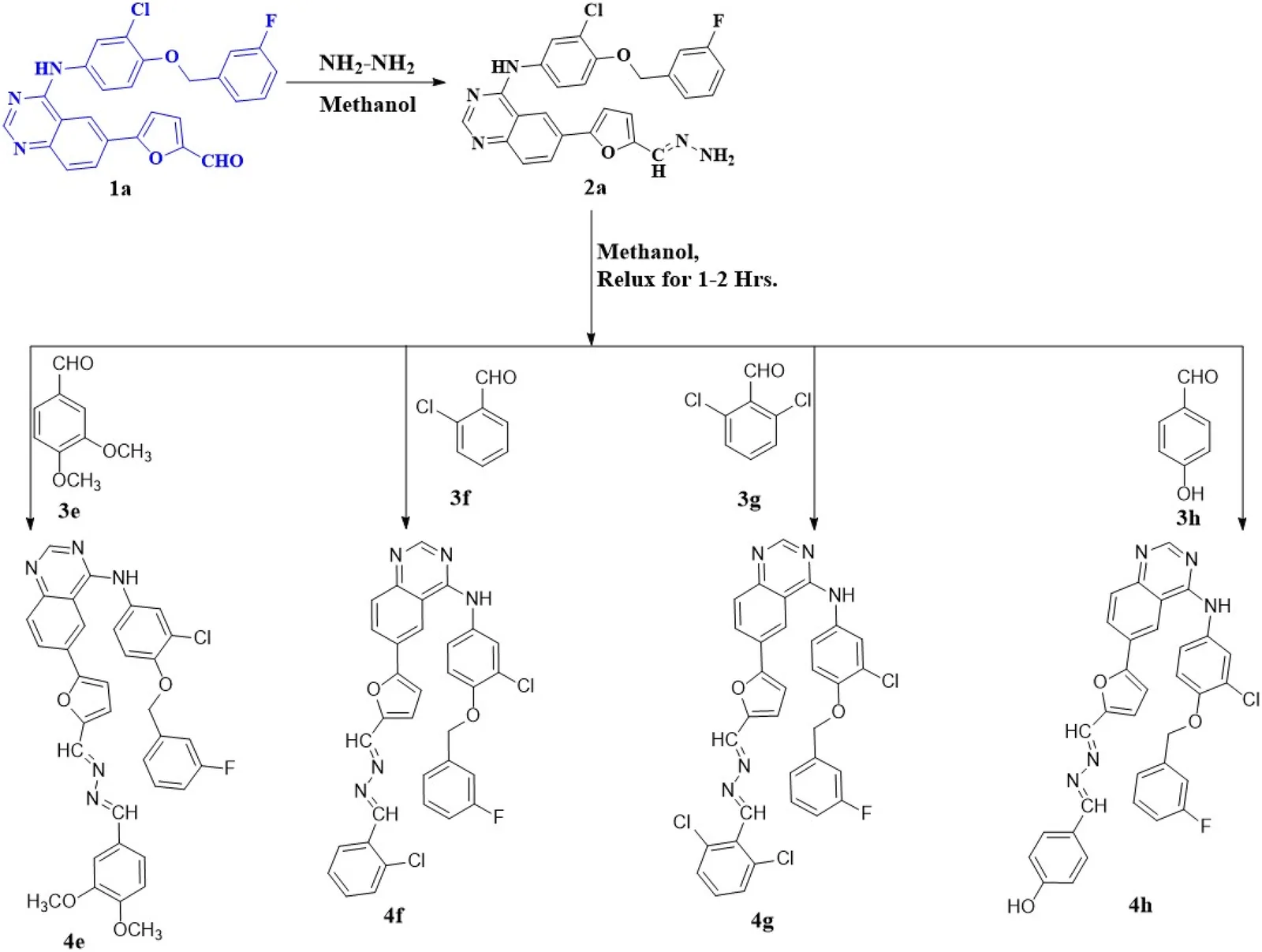
Reaction procedure of synthesised furan-based quinazoline-4-amine derivative scaffolds (4e–h).

#### 2.2.1 Characterization of *N*-(3-chloro-4-((3-fluorobenzyl)oxy)phenyl)-6-(5-((*E*)-(((*E*)-4-methoxybenzylidene)hydrazono)methyl)furan-2-yl)quinazolin-4-amine (4a)

Cream solid; Yield: 87%; M.P.; 240 °C; C_34_H_25_ClFN_5_O_3_; M. Wt.: 606.05 g mol; Elemental Analysis Calc. % (found %): C, 67.38 (67.29); H, 4.16 (4.01); N, 11.56 (11.45); FT-IR (cm^−1^): 3324 (N–H), 2967 (=C–H) Ar stretching, 1602 (–C=C) stretching, 1498 (–C=N) stretching, 1252 (–C–O) stretching, 1058 (–C–F) stretching, 670 (–C–Cl) stretching. ^1^H NMR (DMSO-*d*_6_) *δ* [ppm]: 10.02 (1H, s, N–H), 8.86 (1H, s, Ar–H), 8.71 (1H, s, Ar–H), 8.57 (2H, s, Ar–H), 8.25–8.22 (1H, d, *J* = 8.8 Hz), 7.99 (1H, s, Ar–H), 7.84–7.82 (3H, dd, *J*_1_ = 2.4 Hz, *J*_2_ = 8.4 Hz, Ar–H), 7.79 (1H, m, Ar–H), 7.47–7.46 (1H, m, Ar–H), 7.32–7.26 (5H, m, Ar–H), 7.18–7.10 (2H, m, Ar–H), 7.065–7.043 (1H, d, *J* = 8.8 Hz, Ar–H), 5.25 (2H, s, –OCH_2_), 3.85 (3H, s, –OCH_3_). ^13^C NMR (DMSO-*d*_6_) *δ* [ppm]: 163.95/161.53 (d, ^1^*J*_C–F_ = 243 Hz, Ar C–F), 162.41 (–C–O–CH_3_), 161.96, 158.30, 155.86, 155.36 (Ar, –C–NH–),150.40, 150.14, 150.01 (–C=N–), 140.20/140.13 (d, ^3^*J*_C–F_ = 7 Hz, Ar C-1), 133.47 (Ar, –C=CH–), 131.13, 131.04, 130.75, 129.64 (Ar, –C=CH–),129.21, 127.63, 126.94 (Ar, –C=CH–), 125.00, 123.86, 123.83, 123.20 (Ar, –C=Cl), 121.60, 120.06, 118.78, 115.92 (Ar, –C=CH–), 115.33, 115.13, 114.95, 114.80, 114.66, 114.45, 110.33 (–C=CH–), 69.93 (O–CH_2_), 55.93 (O–CH_3_). ESI-MS (*m*/*z*): 606.74 [M + H]^+^ (calcd 606.17), 608.76, 609.70, 610.77, 415.61, 411.72, 329.62, 257.28.

#### 2.2.2 Characterization of *N*-(3-chloro-4-((3-fluorobenzyl)oxy)phenyl)-6-(5-((*E*)-(((*E*)-4-methylbenzylidene)hydrazono)methyl)furan-2-yl)quinazolin-4-amine (4b)

Yellowish solid; Yield: 89%; M.P.; 242 °C; C_34_H_25_ClFN_5_O_2_; M. Wt.:590.05 g mol^−1^; Elemental Analysis Calc. % (found %): C, 69.21 (69.05); H, 4.27 (4.35); N, 11.87 (11.95); FT-IR (cm^−1^): 3339 (N–H), 1602 (–C=C) stretching, 1490 (–C=N) stretching, 1274 (–C–O) stretching, 1058 (–C–F) stretching, 678 (–C–Cl) stretching. ^1^H NMR (DMSO-*d*_6_) *δ* [ppm]: 10.01 (1H, s, N–H), 8.85 (1H, s, Imine CH), 8.72 (1H, s, Imine CH), 8.57 (2H, s, Ar–H), 8.224–8.22 (1H, d, *J* = 1.6 Hz), 7.99 (1H, s, Ar–H), 7.77 (1H, d, Ar–H), 7.75 (3H, m, Ar–H), 7.47–7.46 (1H, m, Ar–H), 7.34–7.17 (7H, m, Ar–H), 7.17 (1H, m, Ar–H), 5.25 (2H, s, –OCH_2_), 2.35 (3H, s, –CH_3_). ^13^C NMR (DMSO-*d*_6_) *δ* [ppm]: 163.95/161.53 (d, ^1^*J*_C–F_ = 243 Hz, Ar C–F), 162.25, 158.28, 156.01, 155.37 (Ar, –C–NH–), 150.43, 150.40 (–C=N–), 150.16 (–C=N–), 149.88, 141.99, 140.20/140.12 (d, ^3^*J*_C–F_ = 8 Hz, Ar C-1), 133.47, 131.68, 131.12, 131.03, 130.04, 129.20 (Ar, –C=CH–), 128.95, 127.58 (Ar, –C=CH–), 124.98, 123.85, 123.82, 123.18 (Ar, –C=Cl), 121.60, 120.39, 118.84, 115.91 (Ar, –C=CH–), 115.33, 115.12, 114.78, 114.66, 114.44, 110.34 (–C=CH–), 69.92 (O–CH_2_), 21.71 (–C–CH_3_–). ESI-MS (*m*/*z*): 590.75 [M + H]^+^ (calcd 590.18), 592.76, 593.84, 594.78, 329.73, 258.49, 257.28.

#### 2.2.3 Characterization of *N*-(3-chloro-4-((3-fluorobenzyl)oxy)phenyl)-6-(5-((*E*)-(((*E*)-4-chlorobenzylidene)hydrazono)methyl)furan-2-yl)quinazolin-4-amine (4c)

Yellowish solid; Yield: 85%; M.P.; 243 °C; C_33_H_22_Cl_2_FN_5_O_2_; M. Wt.: 610.47 g mol^−1^; Elemental Analysis Calc. % (found %): C, 64.93 (65.06); H, 3.63 (3.59); N, 11.47 (11.35); FT-IR (cm^−1^): 3287 (N–H), 2944 (=C–H) Ar stretching, 1595 (–C=C) stretching, 1483 (–C=N) stretching, 1259 (–C–O) stretching, 1058 (–C–F) stretching, 790 (–C–Cl) stretching. ^1^H NMR (DMSO-*d*_6_) *δ* [ppm]: 10.04 (1H, s, N–H), 8.87–8.77 (2H, s, Imine CH), 8.59–8.58 (2H, d, *J* = 6.4 Hz, Ar–H), 8.25–8.23 (1H, d, *J* = 8.4 Hz, Ar–H), 7.99–7.98 (1H, d, Ar–H), 7.90–7.88 (3H, m, Ar–H), 7.58–7.56 (1H, d, *J* = 8.4 Hz, Ar–H), 7.48 (2H, d, Ar–H), 7.46–7.44 (1H, m, *J* = 8 Hz Ar–H), 7.34–7.27 (5H, m, Ar–H), 7.18 (1H, m Ar–H), 5.26 (2H, s, –OCH_2_). ^13^C NMR (DMSO-*d*_6_) *δ* [ppm]: 163.99/161.53 (d, ^1^*J*_C–F_ = 247 Hz, Ar C–F), 161.10, 158.33, 156.23, 155.45 (Ar, –C–NH–), 151.03, 150.46, 150.18 (–C=N–), 149.73,140.23/140.13 (d, ^3^*J*_C–F_ = 10 Hz, Ar C-1), 136.45, 133.45, 133.26, 131.13, 131.13, 131.05, 130.52, 129.70, 129.58 (Ar, –C=CH–), 129.21, 127.54, 125.01, 123.86, 123.84, 123.21, 121.60, 120.99, 118.95, 115.92, 115.34, 115.13, 114.81, 114.67, 114.45, 110.43 (–C=CH–), 69.92 (O–CH_2_–). ESI-MS (*m*/*z*): 610.64 [M + H]^+^ (calcd 610.12), 612.79, 613.73, 614.67, 615.61, 688.73, 799.75, 473.01, 411.45, 329.46, 257.42.

#### 2.2.4 Characterization of *N*-(3-chloro-4-((3-fluorobenzyl)oxy)phenyl)-6-(5-((*E*)-(((*E*)-thiophen-2-ylmethylene)hydrazono)methyl)furan-2-yl)quinazolin-4-amine (4d)

Yellowish solid; Yield: 86%; M.P.; 240 °C; C_31_H_21_ClFN_5_O_2_S; M. Wt.: 582.05 g mol^−1^; Elemental Analysis Calc. % (found %): C, 63.97 (64.10); H, 3.64 (3.60); N, 12.03 (11.95); FT-IR (cm^−1^): 3332 (N–H), 3056 (=C–H) Ar stretching, 1595 (–C=C) stretching, 1490 (–C=N) stretching, 1252 (–C–O) stretching, 1058 (–C–F) stretching, 700 (–C–Cl) stretching. ^1^H NMR (DMSO-*d*_6_) *δ* [ppm]: 10.03 (1H, s, N–H), 8.94–8.86 (2H, s, Imine CH), 8.57–8.56 (2H, m, *J* = 6.4 Hz, Ar–H), 8.23–8.22 (1H, d, *J* = 1.2 Hz, Ar–H), 7.997–7.991 (1H, d, *J* = 2.4 Hz Ar–H), 7.85 (2H, m, Ar–H), 7.83 (2H, m, Ar–H), 7.46 (1H, m, Ar–H), 7.34–7.22 (5H, m, Ar–H), 7.18 (2H, m Ar–H), 5.26 (2H, s, –OCH_2_). ^13^C NMR (DMSO-*d*_6_) *δ* [ppm]: 163.95/161.53 (d, ^1^*J*_C–F_ = 243 Hz, Ar C–F), 158.30, 156.60, 156.1, 155.40 (Ar, –C–NH–), 150.41, 150.17 (–C=N–), 149.83, 140.20/140.13 (d, ^3^*J*_C–F_ = 7 Hz, Ar C-1), 139.02, 136.97, 134.45, 133.96, 133.46, 131.65, 131.13, 131.04, 129.67 (Ar, –C=CH–), 129.20 128.84, 127.56, 127.32 (Ar, –C=CH–), 124.99, 124.76, 123.86, 123.83, 123.20, 122.93, 121.64, 121.60, 120.76, 119.75, 118.89, 115.91, 115.33, 115.13 (Ar, –C=CH–), 114.80, 114.67, 114.45, 110.41 (–C=CH–), 69.93 (O–CH_2_). ESI-MS (*m*/*z*): 582.68 [M + H]^+^ (calcd 582.12), 583.76, 584.56, 585.64, 586.71, 660.77, 483.89, 411.72, 319.24, 259.43, 257.28.

#### 2.2.5 Characterization of *N*-(3-chloro-4-((3-fluorobenzyl)oxy)phenyl)-6-(5-((*E*)-(((*E*)-3,4-dimethoxybenzylidene)hydrazono)methyl)furan-2-yl)quinazolin-4-amine (4e)

Yellowish solid; Yield: 88%; M.P.; 238 °C; C_35_H_27_ClFN_5_O_4_; M. Wt.: 636.08 g mol^−1^; Elemental Analysis Calc. % (found %): C, 66.09 (66.00); H, 4.28 (4.15); N, 11.01 (11.17); FT-IR (cm^−1^): 3354 (N–H), 2847 (=C–H) Ar stretching, 1610 (–C=C) stretching, 1490 (–C=N) stretching, 1252 (–C–O) stretching, 1028 (–C–F) stretching, 775 (–C–Cl) stretching. ^1^H NMR (DMSO-*d*_6_) *δ* [ppm]: 10.01 (1H, s, N–H), 8.90 (1H, s, Imine CH), 8.85 (1H, s, Imine CH), 8.57–8.56 (2H, d, *J* = 7.2 Hz, Ar–H), 8.25–8.23 (1H, d, *J* = 8.8 Hz, Ar–H), 7.99 (1H, s, Ar–H), 7.98 (1H, d, Ar–H), 7.90 (1H, d, Ar–H), 7.84 (1H, d, Ar–H), 7.34 (1H, m, Ar–H), 7.32–7.27 (5H, m, Ar–H), 7.26 (1H, m, Ar–H), 6.65–6.62 (2H, m, Ar–H), 5.25 (2H, s, –OCH_2_), 3.88–3.86 (3H, s, –OCH_3_), 3.84–3.82 (3H, s, –OCH_3_). ^13^C NMR (DMSO-*d*_6_) *δ* [ppm]: 164.23, 163.95/161.53 (d, ^1^*J*_C–F_ = 243 Hz, Ar C–F), 160.94, 158.28, 157.37, 155.76 (Ar, –C–NH–), 155.34, 150.42, 150.14 (–C=N–),150.10, 149.58, 140.20/140.13 (d, ^3^*J*_C–F_ = 7 Hz, Ar C-1), 133.47, 131.12, 131.04, 129.20 (Ar, –C=CH–), 128.40, 127.66 (Ar, –C=CH–), 125.08, 123.86, 123.83, 123.28 (Ar, –C=Cl), 121.58, 119.72, 118.64, 115.89, 115.33 (Ar, –C=CH–), 115.03, 114.78, 114.67, 114.45, 110.32, 107.21, 98.75, (–C=CH–), 69.92 (O–CH_2_), 56.46 (–C–OCH_3_), 56.08 (–C–OCH_3_). ESI-MS (*m*/*z*): 636.71 [M + H]^+^ (calcd 636.18), 637.79, 638.73, 639.80, 488.60, 331.61, 330.53, 329.59.

#### 2.2.6 Characterization of *N*-(3-chloro-4-((3-fluorobenzyl)oxy)phenyl)-6-(5-((*E*)-(((*E*)-2-chlorobenzylidene)hydrazono)methyl)furan-2-yl)quinazolin-4-amine (4f)

Yellowish solid; Yield: 84%; M.P.; 237 °C; C_33_H_22_Cl_2_FN_5_O_2_; M. Wt.: 610.47 g mol^−1^; Elemental Analysis Calc. % (found %): C, 64.93 (64.99); H, 3.63 (3.60); N, 11.47 (11.42); FT-IR (cm^−1^): 3362 (N–H), 3101 (=C–H) Ar stretching, 1610 (–C=C) stretching, 1490 (–C=N) stretching, 1282 (–C–O) stretching, 1058 (–C–F) stretching, 685 (–C–Cl) stretching. ^1^H NMR (DMSO-*d*_6_) *δ* [ppm]: 10.03 (1H, s, N–H), 9 (1H, s, Imine CH), 8.87 (1H, s, Imine CH), 8.63 (1H, s, Ar–H), 8.57 (1H, s, Ar–H), 8.27–8.24 (1H, d, *J* = 8.4 Hz, Ar–H), 8.1 (1H, d, Ar–H), 7.98 (1H, s, Ar–H), 7.84 (1H, d, Ar–H), 7.7 (1H, d, Ar–H), 7.57–7.46 (4H, m, Ar–H), 7.39–7.34 (5H, m, Ar–H), 7.32–7.26 (1H, t, Ar–H), 5.25 (2H, s, –OCH_2_). ^13^C NMR (DMSO-*d*_6_) *δ* [ppm]: 165.19, 163.95/161.52 (d, ^1^*J*_C–F_ = 244 Hz, Ar C–F), 158.31, 158.03, 156.48, 155.44 (Ar, –C–NH–), 151.68, 150.45, 150.24 (–C=N–), 149.54, 140.2/140.12 (d, ^3^*J*_C–F_ = 8 Hz, Ar C-1), 135.04, 133.41, 133.39 (Ar, –C–Cl), 131.35, 131.13, 131.04, 130.68, 129.77, 129.22 (Ar, –C=CH–), 128.45, 128.21 (Ar, –C=CH2–), 127.45, 125.12, 123.86, 123.32 (Ar, –C=Cl), 121.58, 118.97, 115.88, 115,33, 115.12 (Ar, –C=CH–), 114.79, 114.67, 114.45, 110.51 (–C=CH–), 69.92 (O–CH_2_). ESI-MS (*m*/*z*): 610.77 [M + H]^+^ (calcd 610.12), 612.79, 614.80, 633.76, 638.86, 703.11, 815.34, 840.47, 1388.72, 1466.27, 499.62, 411.72, 351.50, 352.56, 258.63, 257.42.

#### 2.2.7 Characterization of *N*-(3-chloro-4-((3-fluorobenzyl)oxy)phenyl)-6-(5-((*E*)-(((*E*)-2,6-dichlorobenzylidene)hydrazono)methyl)furan-2-yl)quinazolin-4-amine (4g)

Yellowish solid; Yield: 87%; M.P.; 239 °C; C_33_H_21_Cl_3_FN_5_O_2_; M. Wt.: 644.91 g mol^−1^; Elemental Analysis Calc. % (found %): C, 61.46 (61.42); H, 3.28 (3.25); N, 10.86 (10.84); FT-IR (cm^−1^): 3354 (N–H), 3093 (=C–H) Ar stretching, 1610 (–C=C) stretching, 1490 (–C=N) stretching, 1252 (–C–O) stretching, 1028 (–C–F) stretching, 685 (–C–Cl) stretching. ^1^H NMR (DMSO-*d*_6_) *δ* [ppm]: 10.04 (1H, s, N–H), 8.88 (1H, s, Imine CH), 8.84 (1H, s, Imine CH), 8.59–8.57 (2H, s, *J* = 7.6 Hz, Ar–H), 8.25–8.24 (1H, s, *J* = 1.2 Hz, Ar–H), 7.987–7.981 (1H, d, *J* = 2.4 Hz, Ar–H), 7.85 (1H, d, Ar–H), 7.6 (1H, d, *J* = 7.6 Hz, Ar–H), 7.58 (2H, d, *J* = 7.6 Hz, Ar–H), 7.5–7.49 (2H, m, *J* = 7.2 Hz, Ar–H), 7.41–7.4 (1H, d, Ar–H), 7.35–7.31 (4H, m, Ar–H), 7.28–7.26 (1H, m, Ar–H), 5.25 (2H, s, –OCH_2_). ^13^C NMR (DMSO-*d*_6_) *δ* [ppm]: 163.99, 163.99/161.56 (d, ^1^*J*_C–F_ = 244 Hz, Ar C–F), 161.56 (–C=N–), 158.32, 157.53, 156.61, 155.47 (Ar, –C–NH–), 151.68, 150.44, 150.27 (–C=N–), 149.30, 140.24, 140.06, 134.74, 133.42 (Ar, –C–Cl), 132.42 (Ar, –C–Cl), 131.12, 130.72, 129.22, 129.69 (Ar, –C=CH–), 127.44 (Ar, –C=CH–), 125.10, 123.85, 123,82, 123.30 (Ar, –C=Cl), 121.97, 121.59, 119.03, 115.88, 115.32, 115.11 (Ar, –C=CH2–), 114.77, 114.66, 114.44, 110.48 (–C=CH–), 69.91 (O–CH_2_). ESI-MS (*m*/*z*): 644.78 [M + H]^+^ (calcd 644.08), 646.66, 647.73, 649.62, 715.88, 723.54, 1316.95, 638.85, 476.63, 474.48, 388.46, 319.65, 257.28.

#### 2.2.8 Characterization of 4-((*E*)-(((*E*)-(5-(4-((3-chloro-4-((3-fluorobenzyl) oxy) phenyl) amino)quinazolin-6-yl)furan-2-yl)methylene)hydrazono)methyl)phenol (4h)

Cream solid; Yield: 90%; M.P.; 245 °C; C_33_H_23_ClFN_5_O_3_; M. Wt.: 592.03 g mol^−1^; Elemental Analysis Calc. % (found %): C, 66.95 (66.91); H, 3.92 (3.89); N, 11.83 (11.78); FT-IR (cm^−1^): 3056 (N–H), 2981 (=C–H) Ar stretching, 1595 (–C=C) stretching, 1490 (–C=N) stretching, 1215 (–C–O) stretching, 1058 (–C–F) stretching, 678 (–C–Cl) stretching. ^1^H NMR (DMSO-*d*_6_) *δ* [ppm]: 10.16 (1H, s, O–H), 10.02 (1H, s, N–H), 8.86 (1H, s, Imine CH), 8.66 (1H, s, Imine CH), 8.55 (2H, Ar–H), 8.24–8.22 (1H, d, *J* = 8.8 Hz, Ar–H), 7.99 (1H, s, Ar–H), 7.74 (1H, d, Ar–H), 7.72 (3H, m, Ar–H), 7.34 (1H, m, Ar–H), 7.32–7.73 (5H, m, Ar–H), 7.27 (1H, m, Ar–H), 6.94–6.87 (2H, m, Ar–H), 5.25 (2H, s, –OCH_2_). ^13^C NMR (DMSO-*d*_6_) *δ* [ppm]: 163.95/161.53 (d, ^1^*J*_C–F_ = 243 Hz, Ar C–F), 162.27, 161.18, 159.08, 158.29, 156.18, 155.75 (Ar, –C–NH–), 155.32 (Ar, –C–OH), 150.40, 150.10 (–C=N–), 149.61, 148.63, 140.20, 140.12 (=C–NH–)140.20/140.12 (d, ^3^*J*_C–F_ = 8 Hz, Ar C-1), 133.48, 132.62, 131.03, 130.99, 129.64 (Ar, –C=CH–), 129.17, 127.67 (Ar, –C=CH–), 125.41, 125.00, 123.85, 123.20 (Ar, –C=Cl), 121.60, 119.76, 118.73, 116.36, 115.92, 115.33, 115.12 (Ar,–C=CH–), 114.79, 114.67, 114.45, 110.31 (–C=CH–), 69.93 (O–CH_2_). ESI-MS (*m*/*z*): 592.76 [M + H]^+^ (calcd 592.15), 594.78, 595.72, 672.87, 714.27, 1012.51, 411.85, 388.33, 329.59, 258.36, 257.28.

### 2.3 ADMET prediction

The physicochemical and drug-likeness properties of compounds 4a–h and the standard ascorbic acid were predicted using the SwissADME web tool (http://www.swissadme.ch, Swiss Institute of Bioinformatics). Canonical SMILES of each structure were submitted to the server to compute key descriptors: molecular weight, heavy atoms, Csp^3^, rotatable bonds, hydrogen-bond acceptors/donors, and topological polar surface area (TPSA) along with compliance against the Lipinski, Ghose, Veber, Egan, and Muegge drug-likeness filters and the predicted oral bioavailability score, to assess the pharmacokinetic suitability of the synthesized derivatives.^36,37^

### 2.4 Molecular docking study

Molecular docking studies were performed to explore possible binding modes of the synthesized furan–quinazoline hydrazone derivatives (4a–h) using the Schrödinger Suite (Maestro graphical interface, Schrödinger, LLC, New York, NY). The three-dimensional crystal structure of the Kelch domain of Keap1 in complex with a small-molecule inhibitor (PDB ID 4L7B) was retrieved from the RCSB Protein Data Bank and processed using the Protein Preparation Wizard, wherein bond orders were assigned, hydrogen atoms were added, missing side chains were completed, water molecules beyond 5 Å of the co-crystallized ligand were removed, and the protonation states of ionizable residues were optimized at physiological pH (7.0 ± 2.0) using PROPKA/Epik, followed by restrained energy minimization using the OPLS4 force field. The ligand structures of compounds 4a–4h and the standard ascorbic acid were built in Maestro and prepared using LigPrep, which generated the appropriate ionization states at pH 7.0 ± 2.0 (Epik), tautomers, and low-energy 3D conformers using the OPLS4 force field. A receptor grid was generated using the Receptor Grid Generation module, centred on the centroid of the co-crystallized ligand within the Kelch β-propeller binding pocket, with an inner box of 10 Å × 10 Å × 10 Å and an outer box of 20 Å × 20 Å × 20 Å enclosing the binding-site residues. Molecular docking was carried out using Glide in extra-precision (XP) mode, and the resulting poses were ranked according to the Glide docking score (kcal mol^−1^). The best-scoring pose for each ligand was retained for analysis of hydrogen bonding, halogen bonding, π–π stacking, and hydrophobic interactions with the Keap1 binding-site residues.^38^

#### 2.4.1 Validation of the molecular docking protocol

To validate the applied docking protocol, the co-crystallized ligand of Keap1 (PDB ID 4L7B) was extracted from the crystal structure, prepared using LigPrep, and re-docked into the corresponding receptor grid using the same Glide XP protocol and docking parameters employed for the synthesized compounds. As shown in SI 5 (Fig. S1), the re-docked ligand reproduced the experimentally observed binding orientation with an RMSD of 0.87 Å, demonstrating a high degree of agreement between the predicted and crystallographic poses. The corresponding Glide docking score was −6.072 kcal mol^−1^. The 2D interaction analysis further indicated that the re-docked ligand was accommodated within the ligand-binding region of the Keap1 Kelch domain and established multiple hydrophobic, hydrogen-bonding, and other non-covalent interactions with surrounding residues, including Arg326, Leu365, Ala366, Gly367, Cys368, Val369, Gly462, Val463, Gly464, Ala465, Val466, Val467, Val512, Cys513, Gly511, Ala510, Leu557, Gly558, Ile559, Thr560, Val561, Val604, Gly605, Val606, Ala607, Val608, and Leu610. The low RMSD value (<2.0 Å) confirms that the docking protocol can reliably reproduce the experimentally determined ligand-binding pose and therefore supports its use for subsequent docking analysis of compounds 4a–h.

### 2.5 DFT calculations

Density functional theory (DFT) calculations were carried out using the Jaguar module of the Schrödinger Suite (Schrödinger, LLC, New York, NY). The ground-state geometries of compounds 4a–h were fully optimized using the B3LYP hybrid exchange–correlation functional with the 6-31G* basis set, and the optimized structures were confirmed as true energy minima by the absence of imaginary vibrational frequencies. The frontier molecular orbital energies (*E*_HOMO_, *E*_LUMO_), HOMO–LUMO energy gap, global reactivity descriptors, Mulliken atomic charges, and molecular electrostatic potential (MEP) surfaces were derived from the optimized geometries at the same level of theory.^39^

### 2.6 DPPH radical-scavenging assay

The free-radical-scavenging activity of compounds 4a–h was evaluated using the 2,2-diphenyl-1-picrylhydrazyl (DPPH) radical-scavenging assay, with ascorbic acid as the reference standard. Stock solutions of the test compounds and ascorbic acid were prepared in methanol and further diluted with methanol to obtain the required concentrations of 2–10 µg mL^−1^. An aliquot of 1.0 mL of each test solution was mixed with 1.0 mL of freshly prepared 0.1 mM DPPH solution in methanol. The reaction mixtures were thoroughly mixed and incubated in the dark at room temperature for 30 min.

After incubation, the absorbance was measured at 517 nm using a UV-visible spectrophotometer. A control containing 1.0 mL of DPPH solution and 1.0 mL of methanol, without the test compound, was treated under identical experimental conditions. Methanol was used as the blank. The percentage of DPPH radical-scavenging activity was calculated using the following equation:
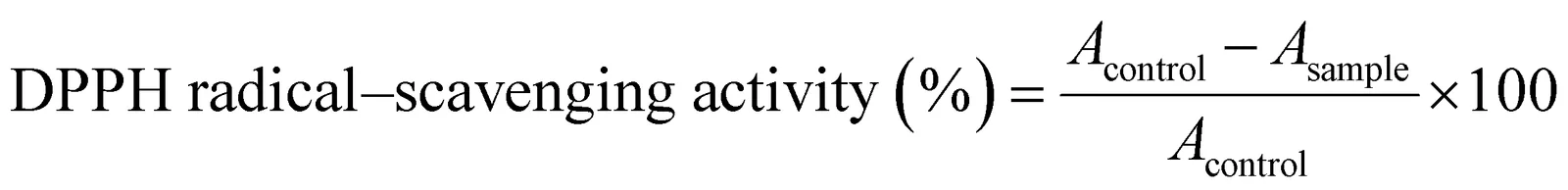
where *A*_control_ is the absorbance of the DPPH control and *A*_sample_ is the absorbance of the reaction mixture containing the test compound. The IC_50_ values, defined as the concentration of the test compound required to inhibit 50% of the DPPH radicals, were determined from the concentration–response curves using nonlinear regression analysis with a four-parameter logistic model. All measurements were performed in triplicate, and the results are expressed as mean ± standard deviation (SD). The IC_50_ values initially obtained in µg mL^−1^ were subsequently converted to µM using the respective molecular weights of the tested compounds.

## 3. Result and discussion

### 3.1 Chemistry

#### 3.1.1 FT-IR spectra

The effective construction of the target scaffold was verified by the FT-IR spectra of the synthesized furan-based quinazoline-4-amine derivatives (4a–h). The quinazoline amine functionality was indicated in all compounds by the presence of a distinctive N–H stretching band in the range of 3056–3362 cm^−1^, whilst bands detected at 1595–1610 cm^−1^ and 1483–1498 cm^−1^ were attributed to aromatic C=C and quinazoline C=N stretching vibrations, respectively. At 3324 cm^−1^ for 4a, 3339 cm^−1^ for 4b, 3287 cm^−1^ for 4c, 3332 cm^−1^ for 4d, 3354 cm^−1^ for 4e and 4g, 3362 cm^−1^ for 4f, and 3056 cm^−1^ for 4h, the broad absorption linked to N–H stretching emerged. The presence of aromatic and heteroaromatic rings in the synthesized compounds was further supported by the observation of aromatic C–H stretching bands in the 2847–3101 cm^−1^ range. The furan moiety's integration into the quinazoline framework was confirmed by the appearance of the furan ring's C–O stretching vibration between 1215 and 1282 cm^−1^. Furthermore, the C–Cl stretching vibrations were found between 670 and 790 cm^−1^, and the C–F stretching bands were found between 1028 and 1058 cm^−1^. These absorptions offer more proof of structural heterogeneity amongst the analogues and are consistent with the presence of halogen substituents in the produced derivatives. The suggested structures of compounds (4a–h) are supported by the measured spectrum data. The presence of N–H, aromatic C=C, quinazoline C=N, furan C–O, and halogen-related stretching bands at the same time suggests that the required substituted products were successfully synthesized and that the quinazoline core remained intact during synthesis. The compounds' slight variations in peak positions are probably caused by variations in the electronic surroundings and substituent effects surrounding the aromatic and heterocyclic systems.

#### 3.1.2 Proton NMR

Strong support for the effective synthesis of the target compounds was provided by the ^1^H NMR spectra of the furan-based quinazoline-4-amine derivatives (4a–h), which were congruent with the suggested structures. The presence of the amine functionality was confirmed in all derivatives by the emergence of a downfield singlet at *δ* 10.01–10.04 ppm, which was attributed to the quinazoline N–H proton. The presence of a hydroxyl substituent in compound 4h was supported by an extra singlet at *δ* 10.16 ppm that matched the phenolic O–H proton. Throughout the series, the quinazoline ring's imine or heteroaromatic protons showed up as singlets in the *δ* 8.84–9.00 ppm range. There were several aromatic resonances between *δ* 6.62 and 8.59 ppm, which is typical for the substituted phenyl and heteroaryl rings as well as the quinazoline nucleus. The creation of the anticipated aromatic framework was further supported by the existence of these signals, as well as their multiplicity patterns and coupling constants.

The methylene resonance at *δ* 5.25–5.26 ppm, which appears as a singlet and corresponds to the –OCH_2_-linker, was a crucial diagnostic signal for all compounds. This signal unmistakably shows that the ether-linked side chain was successfully constructed. Singlets at *δ* 3.85 ppm and *δ* 3.88–3.86/3.84–3.82 ppm, respectively, indicated the presence of methoxy substituents in compounds 4a and 4e. At *δ* 2.35 ppm, compound 4b had a methyl singlet, which is consistent with an aromatic methyl group.

#### 3.1.3 Carbon NMR

The ^13^C NMR spectra of the furan-based quinazoline-4-amine derivatives (4a–h) were fully consistent with the proposed structures and further confirmed the successful synthesis of the target compounds. In all compounds, a pair of signals at *δ* ≈ 163.95 and 161.53 ppm (Δ*δ* ≈ 2.42 ppm, *i.e.* ≈ 243 Hz at 100 MHz) constitutes a doublet arising from one-bond carbon–fluorine coupling (^1^*J*_C–F_) and is assigned to the fluorine-bearing carbon (C-3) of the 3-fluorobenzyl group, rather than to two separate C=N carbons. The remaining deshielded resonances at *δ* ≈ 156–162 ppm were attributed to the quinazoline C=N carbons and other oxygen- or nitrogen-bearing aromatic carbons, indicating the presence of the quinazoline core. Signals observed around *δ* 155–156 ppm were attributed to carbons attached to nitrogen in the heteroaromatic system, while the closely spaced pair near *δ* 140 ppm (*e.g.*, *δ* 140.20/140.13 ppm in 4a, ^3^*J*_C–F_ ≈ 7 Hz) is assigned to the ipso carbon (C-1) of the 3-fluorobenzyl ring rather than to a C=NH carbon. A large number of aromatic carbon signals appeared in the *δ* 110–150 ppm region, which is typical for substituted quinazoline and phenyl rings. These resonances reflected the structural complexity of the derivatives and the presence of multiple substituted aromatic environments. The furan and heteroaryl carbons were also clearly represented within this region, supporting incorporation of the heterocyclic fragment into the final products. Further carbon–fluorine couplings of the 3-fluorobenzyl ring are resolved in this region as closely spaced pairs that recur across the series, *e.g.* for 4a: *δ* 115.33/115.13 and 114.66/114.45 ppm (^2^*J*_C–F_ ≈ 20–21 Hz, C-2 and C-4), *δ* 131.13/131.04 ppm (^3^*J*_C–F_ ≈ 9 Hz, C-5) and *δ* 123.86/123.83 ppm (^4^*J*_C–F_ ≈ 3 Hz, C-6). This characteristic C–F coupling pattern confirms the presence of the intact 3-fluorobenzyloxy fragment in all derivatives.

The methylene carbon of the ether linker was consistently observed at *δ* 69.91–69.93 ppm in all compounds, confirming the presence of the –OCH_2_– bridge. In compounds 4a and 4b, additional aliphatic carbon signals appeared at *δ* 55.93 and 21.71 ppm, respectively, corresponding to –OCH_3_ and aromatic –CH_3_ groups. Compound 4e showed two methoxy signals at *δ* 56.46 and 56.08 ppm, consistent with the presence of two methoxy substituents. In compound 4h, the signal at *δ* 155.32 ppm was assigned to the aromatic carbon bearing the hydroxyl group, supporting the presence of the phenolic substituent. The ^13^C NMR data strongly support the assigned structures of compounds (4a–h). The chemical shifts and number of carbon resonances were in good agreement with the expected substitution patterns, and the data complement the FT-IR and ^1^H NMR results, collectively confirming the formation of the desired furan-based quinazoline-4-amine derivatives.

#### 3.1.4 Mass spectrometry

The electrospray ionization mass spectrometry (ESI-MS) data of the synthesized furan-based quinazoline-4-amine derivatives (4a–h) provided additional confirmation of their molecular identities. In each case, the observed molecular ion peaks were in good agreement with the expected molecular weights of the target compounds, thereby supporting the successful formation of the desired products. The spectra were recorded at nominal (unit) mass resolution, and all observed [M + H]^+^ ions lie within 0.5–0.7 *m*/*z* units of the calculated monoisotopic values given in Section 2.2. For compound 4a, the prominent ion at *m*/*z* 606.74 was assigned to [M + H]^+^, accompanied by isotope peaks at 608.76, 609.70, and 610.77, which are characteristic of halogen-containing molecules. Similarly, compound 4b showed a molecular ion peak at *m*/*z* 590.75 [M + H]^+^ with the expected isotope distribution at 592.76, 593.84, and 594.78. Compound 4c exhibited a molecular ion at *m*/*z* 610.64 [M + H]^+^ together with corresponding isotopic peaks at 612.79, 613.73, 614.67, and 615.61, while compound 4d displayed [M + H]^+^ at *m*/*z* 582.68 with associated isotopic signals at 583.76, 584.56, 585.64, and 586.71. The same pattern was observed for the remaining derivatives. Compound 4e gave a molecular ion at *m*/*z* 636.71 [M + H]^+^, compound 4f at *m*/*z* 610.77 [M + H]^+^, compound 4g at *m*/*z* 644.78 [M]^+^, and compound at 4h*m*/*z* 592.76 [M + H]^+^. The presence of multiple peaks in each spectrum is consistent with fragmentation behavior and isotopic contributions from chlorine (^35^Cl/^37^Cl; fluorine is monoisotopic), further validating the substituted aromatic nature of these compounds. In addition to the molecular ion peaks, several fragment ions were observed in the lower mass region, including signals around *m*/*z* 411, 329, 257, 258, and related values. These fragments likely arose from cleavage of the ether linkage, loss of substituents, and breakdown of the quinazoline-furan framework under ionization conditions. The repeated appearance of fragment ions near *m*/*z* 257–329 across the series suggests a stable common core fragment derived from the quinazoline-based scaffold. A few weak ions at much higher *m*/*z* (*e.g.*, 1388.72 and 1466.27 for 4f, 1316.95 for 4g and 1012.51 for 4h) do not correspond to [M + H]^+^, [M + Na]^+^, [2M + H]^+^ or [2M + Na]^+^ and were not used for structural assignment.

### 3.2 Density functional theory (DFT) study

#### 3.2.1 Molecular geometry

The ground-state geometries of compounds 4a–h, optimized to genuine energy minima, retain the characteristic V-shaped conformation imposed by the central quinazoline-4-amine core flanked by the furan–hydrazone arm on one side and the 3-chloro-4-(3-fluorobenzyloxy)phenyl arm on the other (Fig. 1). The azomethine (–CH=N–N=CH–) linkage connecting the furan and the substituted benzylidene ring is essentially planar in all analogues, favouring extended π-conjugation across the furan–hydrazone–benzylidene framework, a feature expected to facilitate delocalization of an unpaired electron generated upon hydrogen-atom or single-electron transfer during radical scavenging. Minor deviations in the dihedral orientation of the pendant aryl rings among 4a–h arise from the steric and electronic nature of the substituents (methoxy, methyl, chloro, thiophene, hydroxy), which in turn modulate the frontier orbital energies and dipole moments discussed below.

**Fig. 1:**
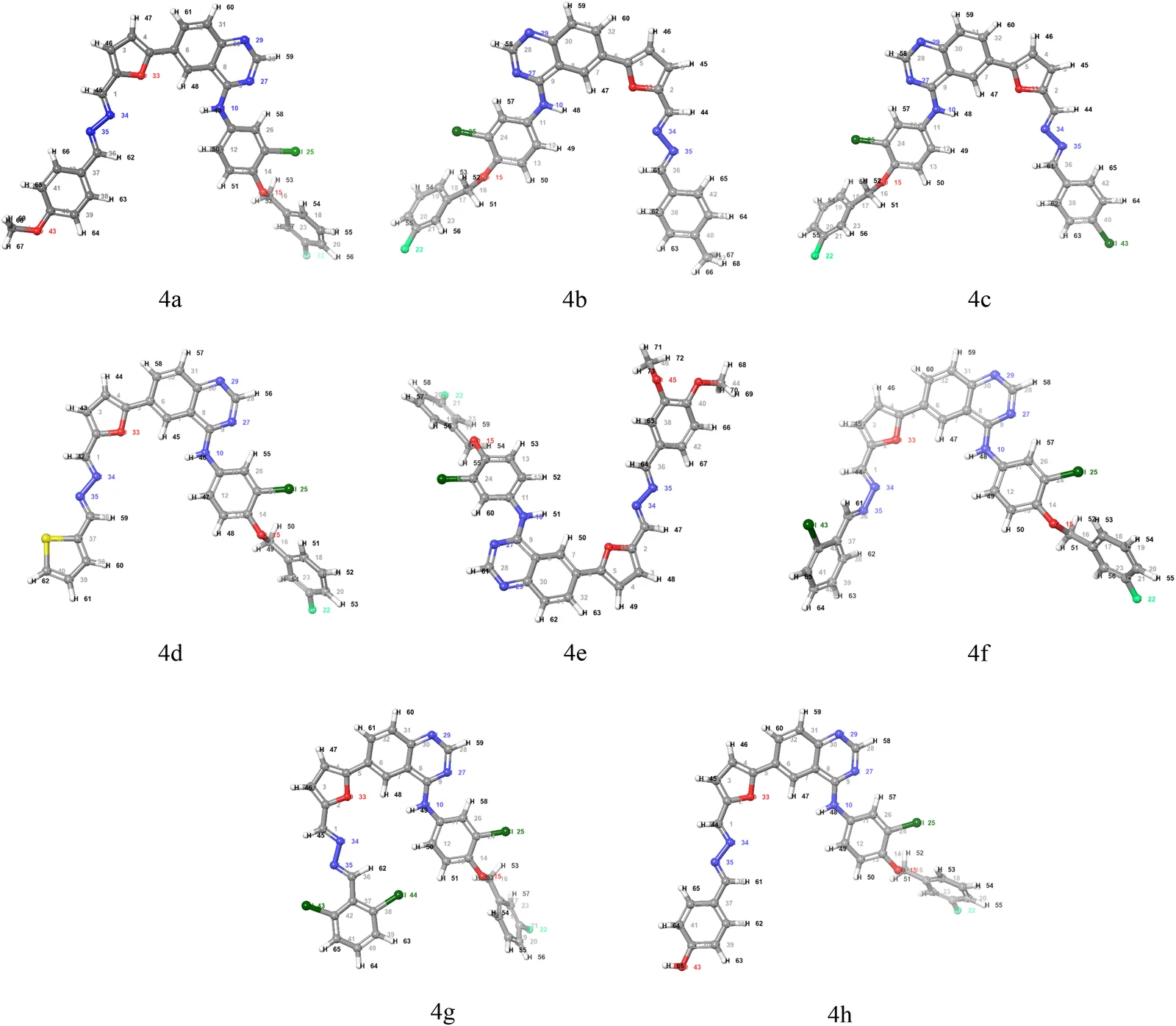
DFT-optimized ground-state molecular geometries of compounds 4a–h showing the atom-numbering scheme used for structural and electronic analysis.

#### 3.2.2 Frontier molecular orbital

The frontier molecular orbital analysis (Fig. 2, Table 1) shows that the HOMO is predominantly delocalized over the furan–hydrazone–benzylidene segment and the electron-rich methoxy-/hydroxy-substituted aryl rings, whereas the LUMO is largely localized on the quinazoline ring and the adjoining azomethine linkage, indicating an intramolecular charge-transfer character on excitation. The calculated *E*_HOMO_ values range from −5.3788 eV (4e) to −5.7397 eV (4g), while the *E*_LUMO_ values span −2.1064 eV (4f) to −2.5437 eV (4c). The resulting HOMO–LUMO energy gap (Δ*E*) varies from 3.1236 eV (4d) to 3.5532 eV (4f); the thiophene-bearing compound 4d and the dimethoxy-substituted compound 4e (Δ*E* = 3.1353 eV) show the narrowest gaps, indicating comparatively higher chemical reactivity and softness, and, in principle, a greater propensity for electron transfer to a free radical. In contrast, the 2-chlorobenzylidene compound 4f displays the widest energy gap (3.5532 eV), consistent with a comparatively more kinetically stable, lower-reactivity electronic configuration.

**Fig. 2:**
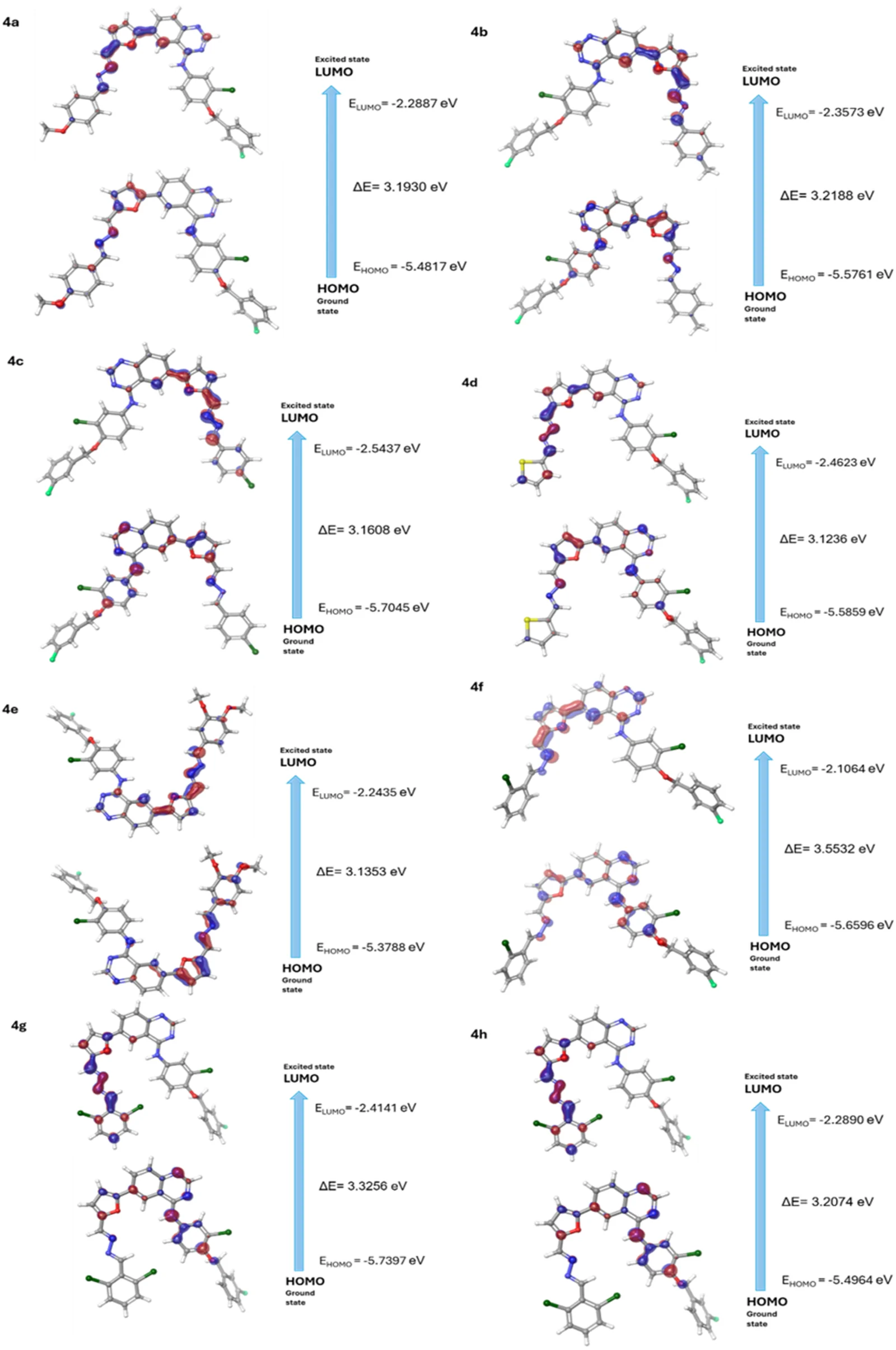
Frontier molecular orbital (HOMO/LUMO) surface plots and the corresponding HOMO–LUMO energy gaps (Δ*E*) of compounds 4a–h.

Frontier molecular orbital energies (*E*_HOMO_, *E*_LUMO_), HOMO–LUMO energy gap (Δ*E*), and global reactivity descriptors (ionization potential, electron affinity, electronegativity, chemical potential, hardness, softness, electrophilicity index) and dipole moment of compounds 4a–h

**Table:** 

Descriptor	Formula	4a (eV)	4b (eV)	4c (eV)	4d (eV)
**Frontier orbital energies**					
*E*_HOMO_	—	−5.4817	−5.5761	−5.7045	−5.5859
*E*_LUMO_	—	−2.2887	−2.3573	−2.5437	−2.4623
Δ*E*	*E*_LUMO_ − *E*_HOMO_	3.1930	3.2188	3.1608	3.1236

**Global reactivity descriptors**					
Ionization potential (*I*)	−*E*_HOMO_	5.4817	5.5761	5.7045	5.5859
Electron affinity (*A*)	−*E*_LUMO_	2.2887	2.3573	2.5437	2.4623
Electronegativity (*χ*)	(*I* + *A*)/2	3.8852	3.9667	4.1241	4.0241
Chemical potential (*µ*)	−*χ*	−3.8852	−3.9667	−4.1241	−4.0241
Hardness (*η*)	(*I* − *A*)/2	1.5965	1.6094	1.5804	1.5618
Softness (*S*)	1/*η*	0.6264	0.6213	0.6328	0.6403
Electrophilicity index (*ω*)	*µ* ^2^/2*η*	4.7275	4.8884	5.3810	5.1842
Dipole moment (*D*)	—	4.1563	4.2598	3.6248	5.8943

**Table:** 

Descriptor	Formula	4e (eV)	4f (eV)	4g (eV)	4h (eV)
**Frontier orbital energies**					
*E*_HOMO_	—	−5.3788	−5.6596	−5.7397	−5.4964
*E*_LUMO_	—	−2.2435	−2.1064	−2.4141	−2.2890
Δ*E*	*E*_LUMO_ − *E*_HOMO_	3.1353	3.5532	3.3256	3.2074

**Global reactivity descriptors**					
Ionization potential (*I*)	−*E*_HOMO_	5.3788	5.6596	5.7397	5.4964
Electron affinity (*A*)	−*E*_LUMO_	2.2435	2.1064	2.4141	2.2890
Electronegativity (*χ*)	(*I* + *A*)/2	3.8112	3.8830	4.0769	3.8927
Chemical potential (*µ*)	−*χ*	−3.8112	−3.8830	−4.0769	−3.8927
Hardness (*η*)	(*I* − *A*)/2	1.5677	1.7766	1.6628	1.6037
Softness (*S*)	1/*η*	0.6379	0.5629	0.6014	0.6236
Electrophilicity index (*ω*)	*µ* ^2^/2*η*	4.6327	4.2434	4.9979	4.7244
Dipole moment (*D*)	—	2.6357	5.2184	2.6984	6.2348

#### 3.2.3 Global reactivity descriptors

The global reactivity descriptors derived from the frontier orbital energies (Table 1) further substantiate the reactivity trends noted above. The chemical hardness (*η*) values range from 1.5618 eV (4d) to 1.7766 eV (4f), mirroring the HOMO–LUMO gap trend, while the corresponding softness (*S*) values (0.5629–0.6403 eV^−1^) indicate that 4d and 4e are the most polarizable and, therefore, the most readily oxidizable members of the series. The electrophilicity index (*ω*), a measure of the energy stabilization a molecule gains upon accepting additional electronic charge, is highest for 4c (5.3810 eV) and lowest for 4f (4.2434 eV), suggesting that 4c behaves as a comparatively stronger electrophile whereas 4f behaves as a comparatively better electron donor. Lower hardness (higher softness) is often associated with easier electron donation to a free radical. However, the measured DPPH activities (Section 3.5) do not follow these global descriptors across the series: 4d, the most active compound, combines the lowest *η* with the narrowest gap, but 4f, the second most active, has the highest *η* (1.7766 eV) and the widest gap (3.5532 eV), and the correlation between Δ*E* and IC_50_ (µM) over the eight compounds is weak and opposite in sign to that expected (*r* = −0.37). The global frontier-orbital descriptors therefore do not account for the observed radical-scavenging trend, which is more likely governed by local properties such as N–H/O–H bond dissociation enthalpies and the stability of the resulting radicals; these were not calculated in the present work. The calculated dipole moments (2.64–6.23 debye) reflect the asymmetric charge distribution imposed by the halogenated aryl and furan–hydrazone arms.

#### 3.2.4 Mulliken charge distributions

The Mulliken atomic charge distribution plots (Fig. 3) reveal a consistent charge-polarization pattern across the series, with the heteroatoms of the quinazoline and furan rings (ring nitrogens, ether/furan oxygen) and the halogen substituents (Cl, F) carrying pronounced negative charges, while the ring and azomethine carbons directly bonded to these electronegative atoms bear compensating positive charges. The imine/azomethine carbons (–CH=N–) consistently display the most positive charge values in each compound, identifying them as the principal electrophilic centres, whereas the furan- and quinazoline-ring nitrogen/oxygen atoms represent the most electron-rich, nucleophilic sites. This charge asymmetry supports facile delocalization of electron density along the conjugated azomethine–furan–quinazoline backbone and provides favourable sites for donor–acceptor interactions with the Keap1 Kelch domain, as well as for single-electron/hydrogen-atom abstraction from DPPH radicals during the *in vitro* antioxidant assay.

**Fig. 3:**
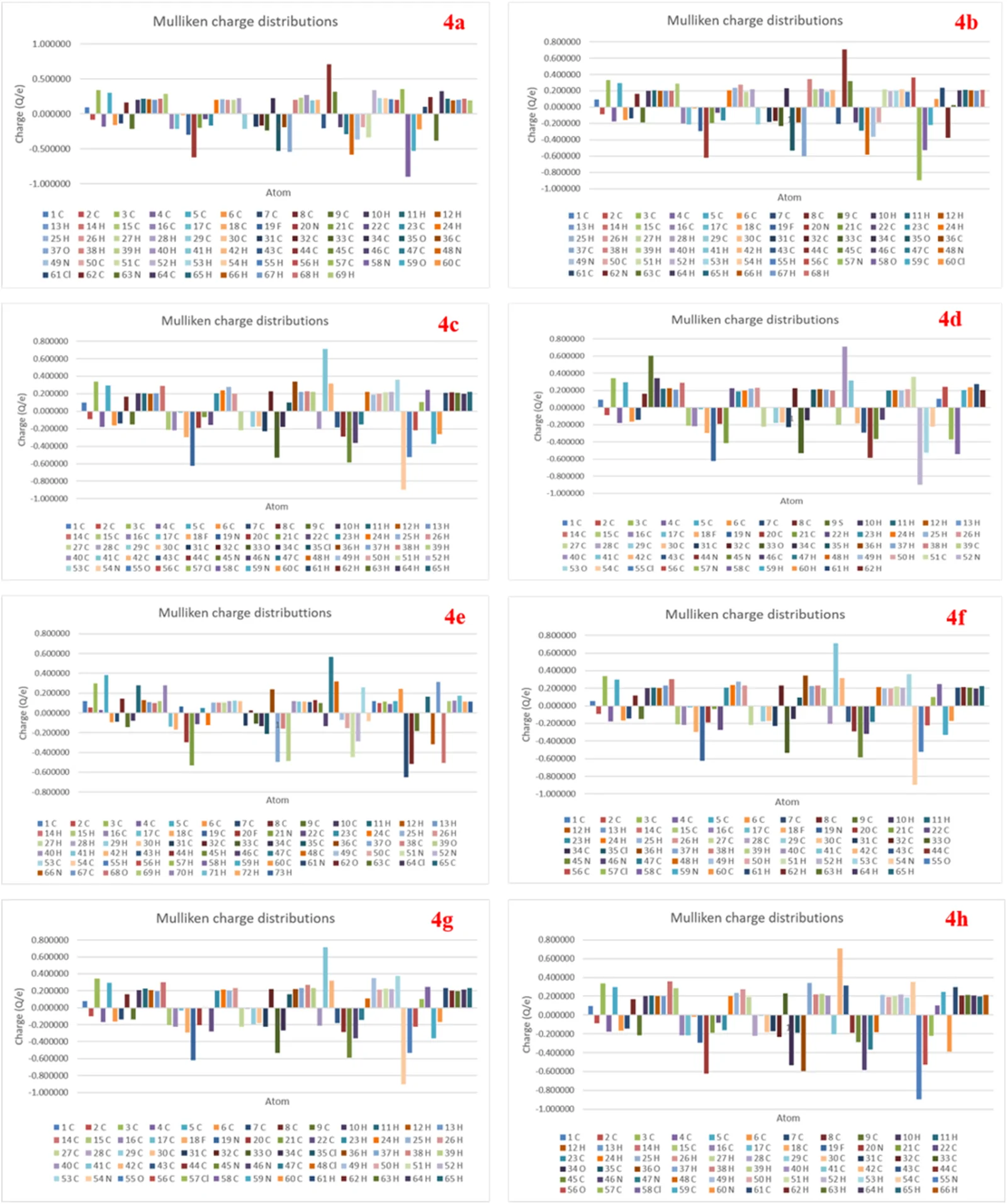
Mulliken atomic charge distribution profiles of compounds 4a–h.

#### 3.2.5 Molecular electrostatic potential

The molecular electrostatic potential (MEP) surfaces (Fig. 4), mapped on the total electron density with the standard red (electron-rich, nucleophilic) to blue (electron-deficient, electrophilic) colour scale, show the most negative potential (red regions) localized over the furan-ring oxygen, the azomethine/imine nitrogen atoms, and, in compound 4h, the phenolic oxygen, marking these as the preferred sites for electrophilic attack and hydrogen-bond acceptance. Conversely, the most positive potential (blue regions) is concentrated over the quinazoline N–H and aromatic C–H hydrogens, consistent with their role as hydrogen-bond donors, such as the backbone hydrogen bond to Val420 observed in several of the docked poses (Section 3.4). The coexistence of well-defined electron-rich (furan/azomethine) and electron-poor (N–H) regions within a single, extended conjugated framework is a favourable electronic signature for radical-scavenging molecules, since it enables both hydrogen-atom donation from the N–H/aryl–OH groups and stabilization of the resulting radical by delocalization over the electron-rich heteroaromatic system.

**Fig. 4:**
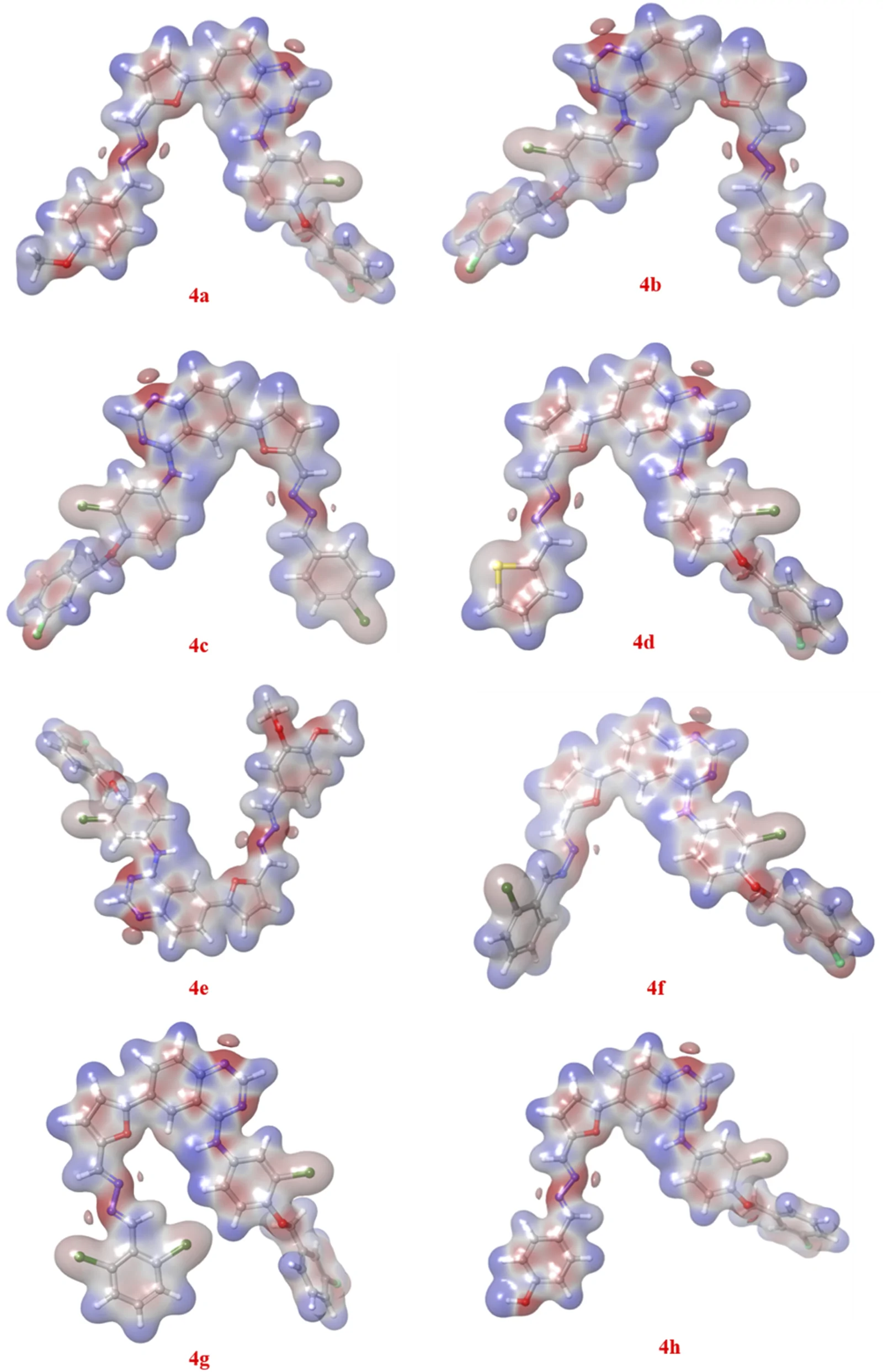
Molecular electrostatic potential (MEP) surface maps of compounds 4a–h (red = electron-rich/nucleophilic region; blue = electron-deficient/electrophilic region).

### 3.3 ADMET analysis

The predicted physicochemical and drug-likeness properties of compounds 4a–h, computed using SwissADME, are summarized in Table 2 and visualized as bioavailability radar plots in Fig. 5. All eight derivatives possess molecular weights in the range of 582.05–644.91 g mol^−1^, appreciably higher than the 500 g mol^−1^ threshold recommended by Lipinski's rule of five, a consequence of the extended furan–hydrazone–quinazoline architecture bearing halogenated aryl substituents. Consistently, all compounds returned at least one Lipinski violation on the basis of molecular weight alone, while 4a, 4b, 4c, 4d, 4f, and 4g accumulated a second violation (attributable to a calculated lipophilicity beyond the recommended limit), whereas the more polar dimethoxy- (4e) and phenolic (4h) analogues incurred only a single violation, reflecting their comparatively lower lipophilicity. The topological polar surface area (TPSA) values (84.90–113.14 Å^2^) remain below the ∼140 Å^2^ threshold generally associated with poor passive membrane permeability, and the number of rotatable bonds (9–11) and hydrogen-bond acceptors/donors (7–9 HBA; 1–2 HBD) fall largely within acceptable medicinal-chemistry ranges, with the exception of 4e, which alone registers a Veber-rule violation owing to its eleven rotatable bonds. In line with these trends, the calculated oral bioavailability scores are 0.55 for 4e and 4h, appreciably higher than the 0.17 recorded for the remaining six analogues, a difference that is also visually reflected in the more balanced, contracted radar profiles of 4e and 4h relative to the LIPO/INSATU-skewed profiles of the other derivatives (Fig. 5). Notably, this places the two most drug-like compounds of the series (4e, 4h) in partial contrast with the two most potent radical scavengers identified by the DPPH assay (4d, 4f; Section 3.5) a common trade-off encountered in early-stage lead identification between potency and pharmacokinetic optimality, which could be addressed in future analogues through targeted reduction of molecular weight and lipophilicity without compromising the core radical-scavenging pharmacophore. As expected for a small, highly polar natural antioxidant, ascorbic acid violated none of the Lipinski, Ghose, Veber, Egan, or Muegge filters and returned the highest bioavailability score (0.56) among all compounds evaluated.

**Table 2:** Physicochemical, drug-likeness, and bioavailability parameters of compounds 4a–h and ascorbic acid predicted using SwissADME

Sr. no	Physiological properties					Drug likeness properties (#violations)							
	MW	Heavy atoms	Csp^3^	*n*ROTB	HBA	HBD	TPSA	Lipinski	Ghose	Veber	Egan	Muegge	Bio. score
4a	606.05	44	0.06	10	8	1	94.13	2	3	0	1	2	0.17
4b	590.05	43	0.06	9	7	1	84.9	2	3	0	1	1	0.17
4c	610.46	43	0.03	9	7	1	84.9	2	3	0	1	2	0.17
4d	582.05	41	0.03	9	7	1	113.14	2	3	0	1	1	0.17
4e	636.07	46	0.09	11	9	1	103.36	1	4	1	1	2	0.55
4f	610.46	43	0.03	9	7	1	84.9	2	3	0	1	2	0.17
4g	644.91	44	0.03	9	7	1	84.9	2	3	0	1	2	0.17
4h	592.02	43	0.03	9	8	2	105.13	1	3	0	1	1	0.55
Ascorbic acid	176.12	12	0.5	2	6	4	107.22	0	2	0	0	1	0.56

**Fig. 5:**
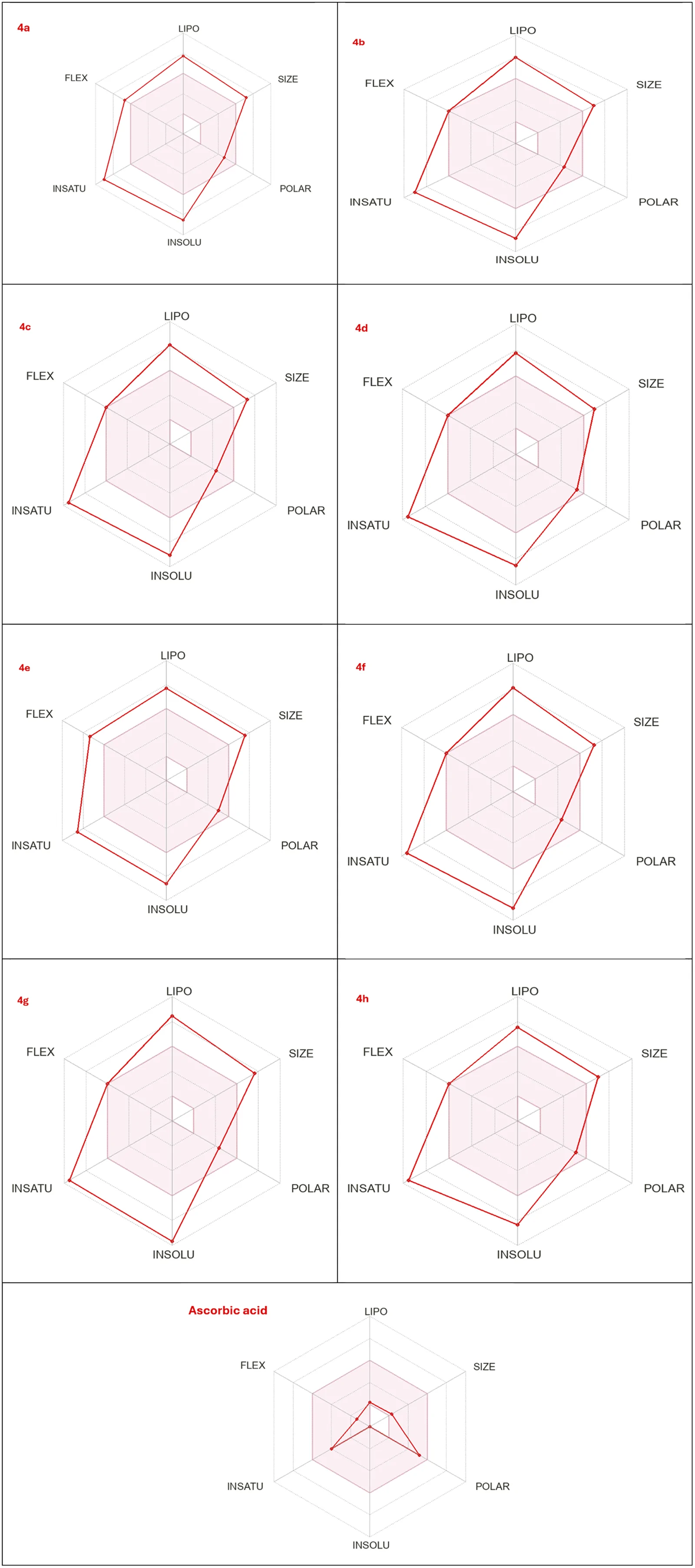
SwissADME bioavailability radar plots of compounds 4a–h and ascorbic acid; the pink hexagonal region represents the optimal physicochemical range for oral bioavailability (LIPO, SIZE, POLAR, INSOLU, INSATU, FLEX).

### 3.4 Molecular docking results

All the synthesized derivatives (4a–h) were docked into the Kelch domain of Keap1 (PDB 4L7B), giving Glide docking scores ranging from −7.325 to −8.603 kcal mol^−1^ (Table 3); ascorbic acid, which was docked only because it is the standard of the DPPH assay and is not an established Keap1 ligand, gave −7.541 kcal mol^−1^. Compound 4f gave the most favourable docking score (−8.603 kcal mol^−1^), followed by 4d (−8.325 kcal mol^−1^) and 4g (−8.126 kcal mol^−1^), while 4h and 4a gave less favourable scores (−7.325 and −7.549 kcal mol^−1^, respectively). A hydrogen bond to the backbone of Val420 was consistently observed for 4a, 4b, 4f and 4h, anchoring the quinazoline nitrogen within the Kelch β-propeller cavity, whereas 4d and 4e formed a halogen bond between Arg326 and the chlorine atom of the common 3-chloro-4-(3-fluorobenzyloxy)phenyl arm, and 4g interacted with Arg326 through a ring nitrogen. Compound 4g, bearing two ortho-chlorine atoms, formed the most extensive halogen-bonding network (five halogen contacts with Val418, Val465, Val606, Ile559, and Asn517) alongside a cation–π-type contact between Arg326 and the hydrazone ring. Across the series, the hydrophobic pocket lined by Leu365, Ala366, Cys368, Ile416, Val418, Cys513, Val514, Leu515, Ile559, Thr560, Val561, and the Val604–Ala607 loop provided the principal stabilizing contacts for the quinazoline-furan core, mirroring the hydrophobic contacts made by ascorbic acid within the same pocket (Ile559, Leu557, Gly558, Val604/605, Ala510, Gly511, Cys513, Ala366). Although the re-docked reference ligand of 4L7B occupies the Nrf2-binding pocket (Section 2.4; Fig. S1), it should be noted that the contacts in the poses of 4a–h are made mainly with hydrophobic valine, leucine and isoleucine residues, and none of the canonical Nrf2-binding residues of the Kelch domain (*e.g.*, Tyr334, Ser363, Arg380, Asn382, Arg415, Arg483, Ser508, Tyr525, Gln530, Ser555, Tyr572 and Ser602) appears in the contact lists (Table 3). The docking results therefore do not show that these compounds occupy the Nrf2-binding site, and any effect on the Keap1–Nrf2 interaction remains an *in silico* hypothesis that would require cellular studies (*e.g.*, Nrf2 nuclear translocation, ARE-reporter or HO-1/NQO1 expression assays) to test. Likewise, although 4f and 4d gave the two most favourable docking scores and were also the two most active compounds in the DPPH assay, the DPPH assay is a cell-free chemical test that does not involve Keap1, so this coincidence is not evidence of a mechanistic link (Fig. 6).

**Table 3:** Glide docking scores (kcal mol^−1^) and key binding interactions of compounds 4a–h and ascorbic acid within the Kelch domain of Keap1 (PDB 4L7B)

Compound	Binding energy (kcal mol^−1^)	H-bonds (backbone)	Halogen bonds	π-Interactions	Key hydrophobic contacts
4a	−7.549	Val420	—	—	Leu365, Ala366, Cys368, Ile416, Val418, Ile559, Thr560, Val561, Cys513, Val514, Leu515, Val604, Val606, Ala607
4b	−8.087	Val420	—	—	Leu365, Ala366, Cys368, Ile416, Val418, Val512, Cys513, Val514, Leu515, Val467, Leu468, Ile559, Thr560, Val561, Val604/606
4c	−7.891	—	—	—	Leu365, Ala366, Gly367/368, Val369/370, Val463–467, Ile416, Val418, Ile421, Val512–514, Val604 (purely hydrophobic pocket; no polar contacts marked)
4d	−8.325	—	Arg326 → Cl	Hie516 ↔ fluorophenyl ring (π–π)	Leu365, Cys368, Val369, Ile559, Thr560, Val561, Val604/606/608, Ala607, Leu610, Cys513, Val512/514, Leu515
4e	−8.038	Val467	Arg326 → Cl	—	Ile416, Val418, Val463/465, Ala466, Leu365, Ala366, Val514, Leu515, Ala556, Leu557, Ile559, Val561, Val604/606/608
4f	−8.603	Val420	—	—	Leu365, Ala366, Cys368, Ile416, Val418, Cys513, Val514, Leu515, Ile559, Thr560, Val561, Val604/606
4g	−8.126	Arg326 → ring N; Val514 → HN	Val418 → Cl; Val465 → Cl; Val606 → Cl; Ile559 → Cl; Asn517 → Cl (5 total)	—	Ile416, Leu365, Ala366, Val463/467/468, Cys513, Leu515, Val604/608/610
4h	−7.325	Val420	—	—	Leu365, Ala366, Cys368, Ile416, Val418, Val514, Leu515, Ile559, Thr560, Val561, Val604–607
Ascorbic acid	−7.541	Val418; Val606; Gly367; Val512; Val465 (5 total)	—	—	Ile559, Leu557, Gly558, Val604, Gly605, Ala510, Gly511, Cys513, Ala366

**Fig. 6:**
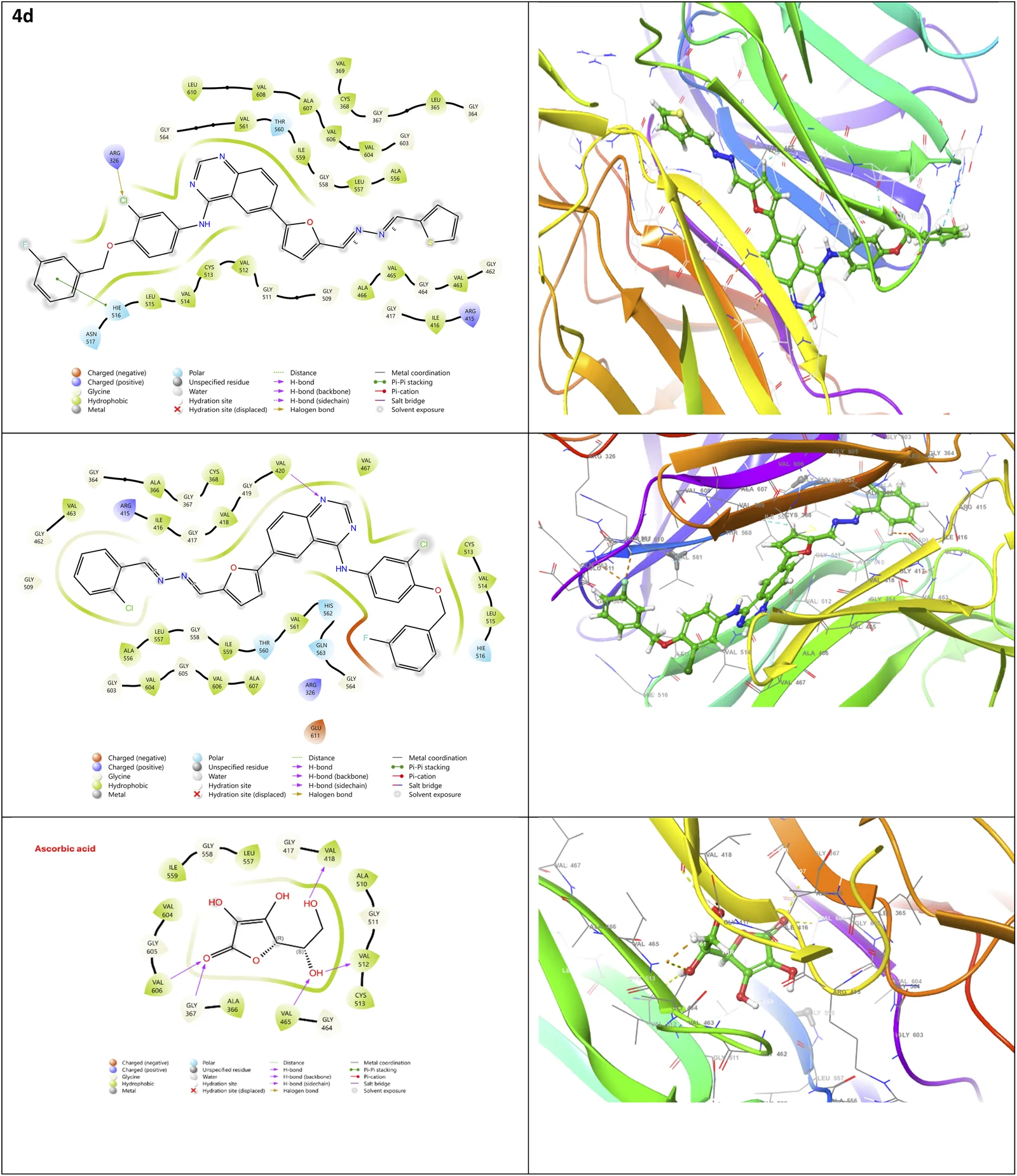
Two-dimensional ligand–interaction diagrams (left) and the corresponding three-dimensional docked binding pose within the Kelch β-propeller domain of Keap1 (right) for the representative compounds 4d, 4f, and ascorbic acid (PDB 4L7B).

### 3.5 Antioxidant assay

The *in vitro* free-radical-scavenging potential of the synthesized furan–quinazoline hydrazone derivatives (4a–h) was evaluated using the DPPH radical-scavenging assay, and the results are summarized in Table 4. All eight compounds exhibited concentration-dependent scavenging of the stable DPPH radical, with IC_50_ values of 85.5 ± 3.7 to 148.1 ± 3.2 µM (49.74–90.40 µg mL^−1^), compared with 235.2 ± 4.6 µM (41.42 µg mL^−1^) for the standard antioxidant ascorbic acid. Because the derivatives (MW 582–645 g mol^−1^) are more than three times heavier than ascorbic acid (176.12 g mol^−1^), potencies are compared on a molar basis; expressed in µM, all eight compounds were more potent than ascorbic acid in this assay. Among the series, compound 4d (IC_50_ = 85.5 ± 3.7 µM), bearing a thiophen-2-ylmethylene arm, and compound 4f (IC_50_ = 90.8 ± 3.8 µM), bearing a 2-chlorobenzylidene arm, emerged as the most potent radical scavengers, followed by 4a (108.0 ± 2.7 µM, 4-methoxybenzylidene) and 4h (108.3 ± 6.6 µM, phenolic –OH substituent). Compounds 4b, 4g, 4e, and 4c displayed comparatively weaker scavenging activity (IC_50_ = 118.2–148.1 µM), with the 3,4-dimethoxy- (4e) and 4-chloro-substituted (4c) analogues being the least active members of the series. The activity order does not follow the global DFT descriptors (Section 3.2.3): 4d has the narrowest HOMO–LUMO gap, but 4f has the widest, and Δ*E* shows only a weak correlation with IC_50_ (*r* = −0.37). Likewise, the docking results (Section 3.4) cannot explain the DPPH data, because this cell-free assay does not involve Keap1. Notably, the presence of an electron-rich heteroaromatic thiophene ring (4d) or a halogen substituent capable of stabilizing the resulting radical through inductive/resonance effects (4f) appears to enhance radical-scavenging efficiency more effectively than simple alkoxy substitution (4e), while the free phenolic –OH group in 4h, although classically associated with antioxidant activity *via* direct H-atom donation, showed only moderate activity in this scaffold, suggesting that the overall electronic environment of the conjugated furan–quinazoline–hydrazone framework, rather than any single substituent, governs the radical-scavenging efficiency of this series. Overall, these results indicate that the furan-Schiff-based quinazoline-4-amine scaffold constitutes a promising, structurally tunable antioxidant pharmacophore, with compounds 4d and 4f warranting further investigation as lead candidates. Concentration–inhibition plots for compounds 4a–h are shown in SI 6.

**Table 4:** DPPH radical-scavenging activity of furan-based quinazolin-4-amine hydrazone derivatives (4a–h) and ascorbic acid^a^

Compound	IC_50_, µM (µg mL^−1^), mean ± SD
4a	108.0 ± 2.7 (65.44 ± 1.65)
4b	118.2 ± 4.0 (69.77 ± 2.37)
4c	148.1 ± 3.2 (90.40 ± 1.98)
4d	85.5 ± 3.7 (49.74 ± 2.13)
4e	136.5 ± 3.8 (86.85 ± 2.39)
4f	90.8 ± 3.8 (55.44 ± 2.34)
4g	123.2 ± 5.2 (79.44 ± 3.34)
4h	108.3 ± 6.6 (64.12 ± 3.89)
Ascorbic acid	235.2 ± 4.6 (41.42 ± 0.81)

a Data were expressed as mean ± SD of three experiments; µM values were calculated from the µg mL^−1^ values using the molecular weight of each compound (ascorbic acid, 176.12 g mol^−1^).

## 4. Conclusion

In summary, a new series of furan-based quinazoline-4-amine hydrazone (Schiff base) derivatives (4a–h) was successfully designed and synthesized in good yields (84–90%) through a sequential nucleophilic aromatic substitution, Suzuki–Miyaura cross-coupling, and hydrazone condensation strategy, and unambiguously characterized by FT-IR, ^1^H NMR, ^13^C NMR, and ESI-mass spectrometry. DFT calculations revealed that all the synthesized compounds adopt an extended, planar azomethine-conjugated geometry, with frontier orbital energy gaps (Δ*E* = 3.12–3.55 eV), global reactivity descriptors, Mulliken charge distributions, and molecular electrostatic potential surfaces were obtained for all compounds, although the global descriptors did not account for the measured radical-scavenging trend. Molecular docking of the synthesized derivatives against the Kelch domain of Keap1 (PDB 4L7B), the master negative regulator of the Nrf2-ARE antioxidant signalling pathway, gave docking scores of −7.325 to −8.603 kcal mol^−1^ with poses stabilised mainly by hydrophobic contacts; because these poses do not involve the Nrf2-binding residues and no cellular assays were performed, any modulation of the Keap1–Nrf2 pathway remains an *in silico* hypothesis. Computational ADMET profiling revealed generally drug-like physicochemical properties across the series (molecular weight 582.05–644.91 g mol^−1^; TPSA 84.90–113.14 Å^2^), with the dimethoxy- and phenolic-substituted analogues 4e and 4h showing only a single Lipinski violation and the highest predicted oral bioavailability scores (0.55) of the series, in partial trade-off with the superior radical-scavenging potency of 4d and 4f. Finally, the *in vitro* DPPH radical-scavenging assay confirmed the antioxidant potential of the series, with all eight compounds more potent than ascorbic acid on a molar basis and 4d (IC_50_ = 85.5 ± 3.7 µM) and 4f (IC_50_ = 90.8 ± 3.8 µM) emerging as the most active analogues. Taken together, these findings support the furan-Schiff-based quinazoline-4-amine scaffold as a promising and structurally tunable platform for the future development of synthetic antioxidant agents, warranting cell-based antioxidant and Keap1–Nrf2 pathway studies and further lead optimization of the most active candidates.

## Author contributions

Sunil D. Narode: methodology, investigation, formal analysis. Miral Vora: writing – original draft, visualization, software, writing – review & editing, validation, supervision, methodology, investigation, data curation, conceptualization. Jay B. Maheta: visualization, investigation, formal analysis. Yogesh O. Bhola: software, data curation, writing – original draft. Darshna K. Lakhnotra: software, formal analysis, data curation. Nikita J. Patel: visualization.

## Conflicts of interest

The authors affirm that the work described in this publication was not influenced by any known competing financial interests or personal relationships.

## Supplementary Material

RA-016-D6RA08647K-s001

## Data Availability

Additional raw data are available from the corresponding author upon reasonable request. All data supporting the findings of this study, including NMR, FT-IR, and mass spectra, computational DFT data, molecular docking results, and ADME analysis, are provided in the supplementary information (SI) accompanying this manuscript. Supplementary information is available. See DOI: https://doi.org/10.1039/d6ra08647k.
