# Supplementary material for: Rational design and synthesis of furan-based Schiff base quinazolin-4-amine hybrids: spectral elucidation, molecular docking, DFT studies, and in silico ADMET-antioxidant study
Source: RSC Adv. 2026 Oct 20;16(48):64680–98. doi: 10.1039/d6ra08647k (PMC13682218; doi:10.1039/d6ra08647k)
Supplement: Supplementary file 1 [file RA-016-D6RA08647K-s001.pdf]

Supplementary Information (SI) for RSC Advances.
This journal is © The Royal Society of Chemistry

Rational Design and Synthesis of Furan-Based Schiff Base Quinazolin-4-amine Hybrids: Spectral Elucidation, Molecular Docking, DFT Studies, and *In Silico* ADMET-Antioxidant study

Sunil D. Narode[1], Miral Vora*[1], Jay B. Maheta[2], Yogesh O. Bhola[2], Darshna K. Lakhnotra[2], Nikita J. Patel[1]

[1] Department of Chemistry, Krishna School of Science,
Drs. Kiran & Pallavi Patel Global University, (KPGU) Vadodara, Gujarat, India.
[2] Department of Chemistry, Shri M. P. Pandya Science College, Lunawada, Mahisagar, Shri Govind Guru University, Godhra, Gujarat, India.
**Department of Chemistry, Krishna School of Science,*
Drs. Kiran & Pallavi Patel Global University, (KPGU) Vadodara, Gujarat, India.
Corresponding author e-mail: patelmiral902@gmail.com

Supplementary Material: 1. FT-IR spectra of synthesised compounds (4a to 4h)

Compound 4a

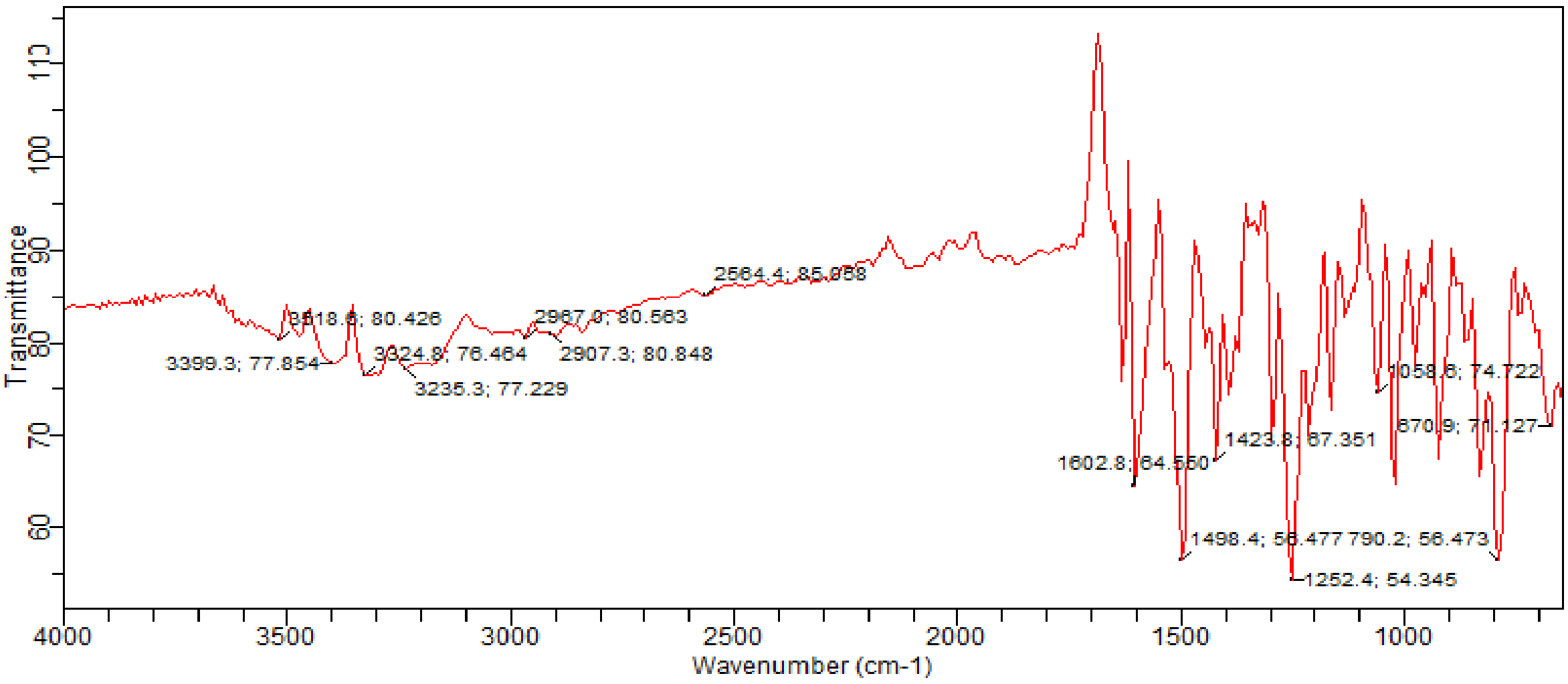

Compound 4b

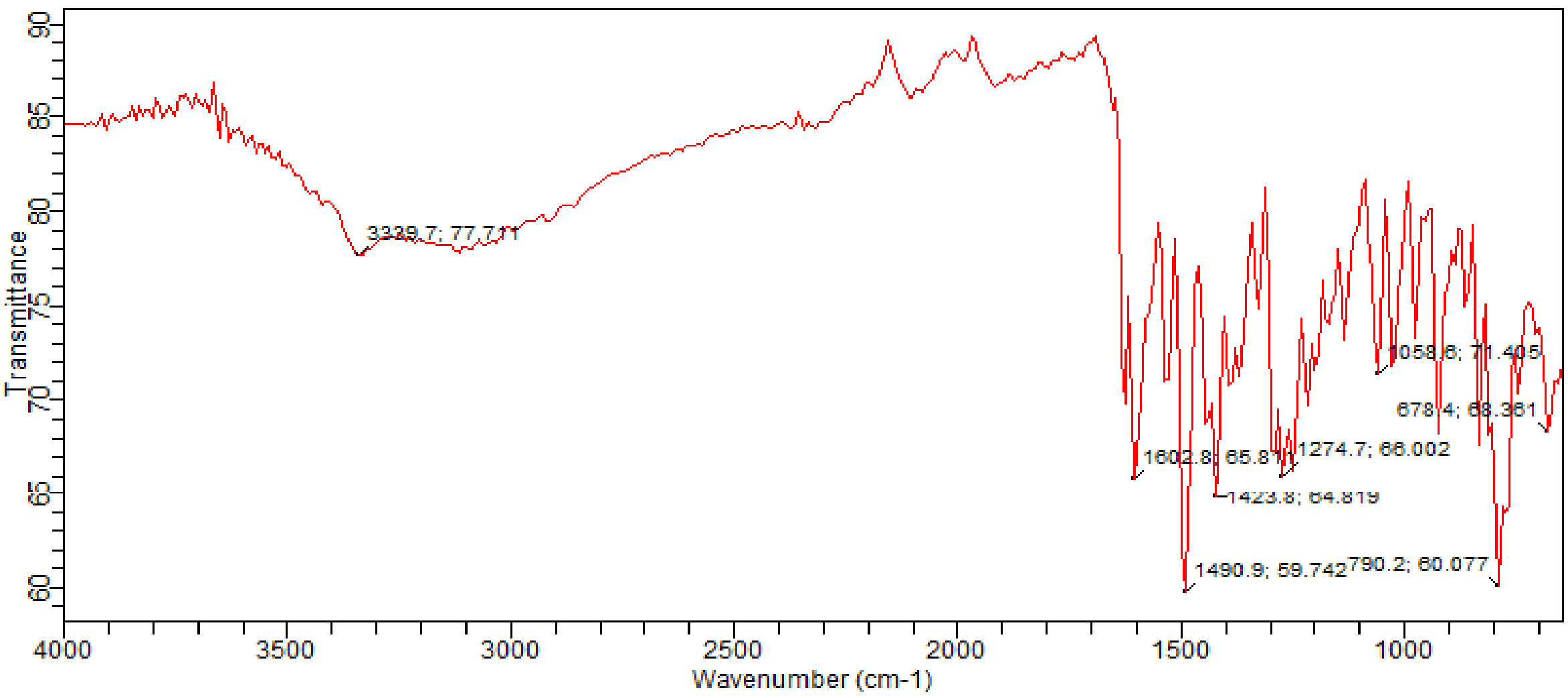

Compound 4c

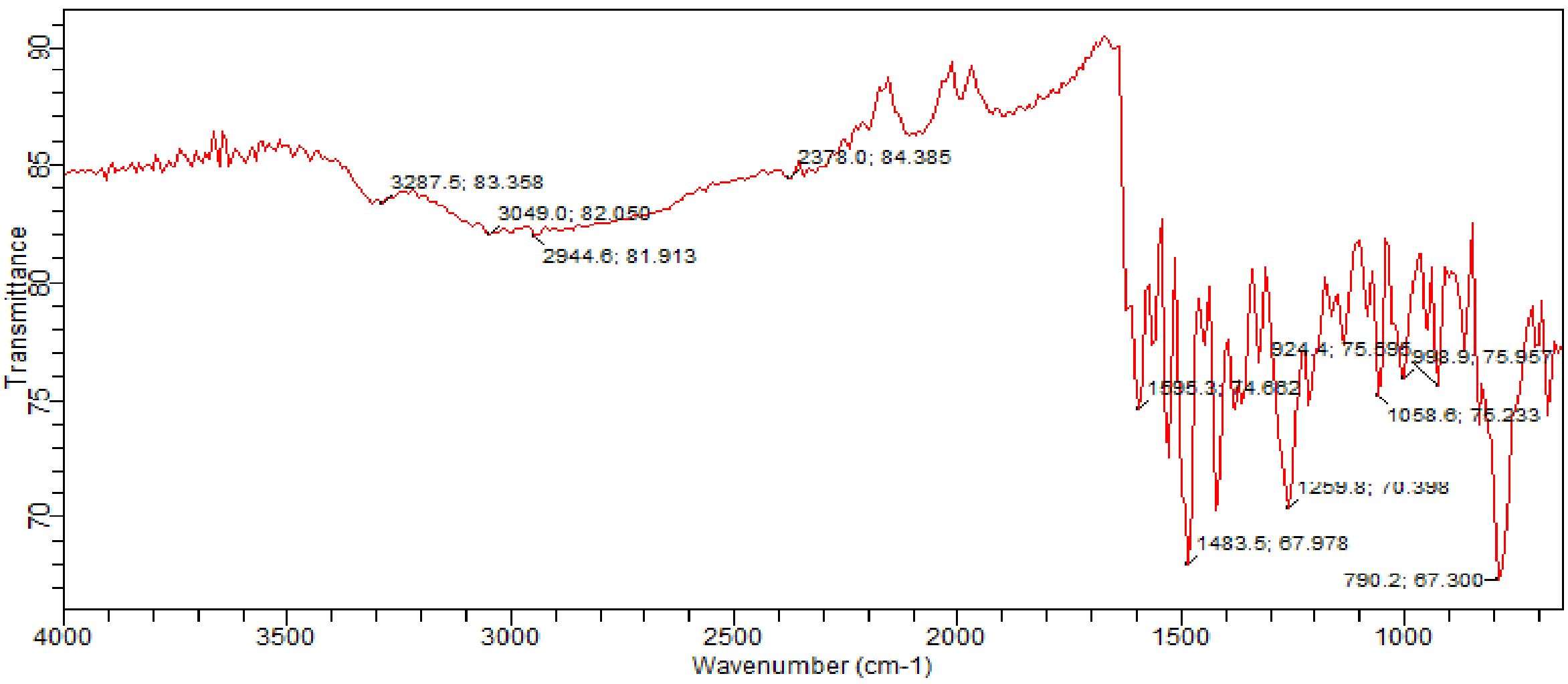

Compound 4d

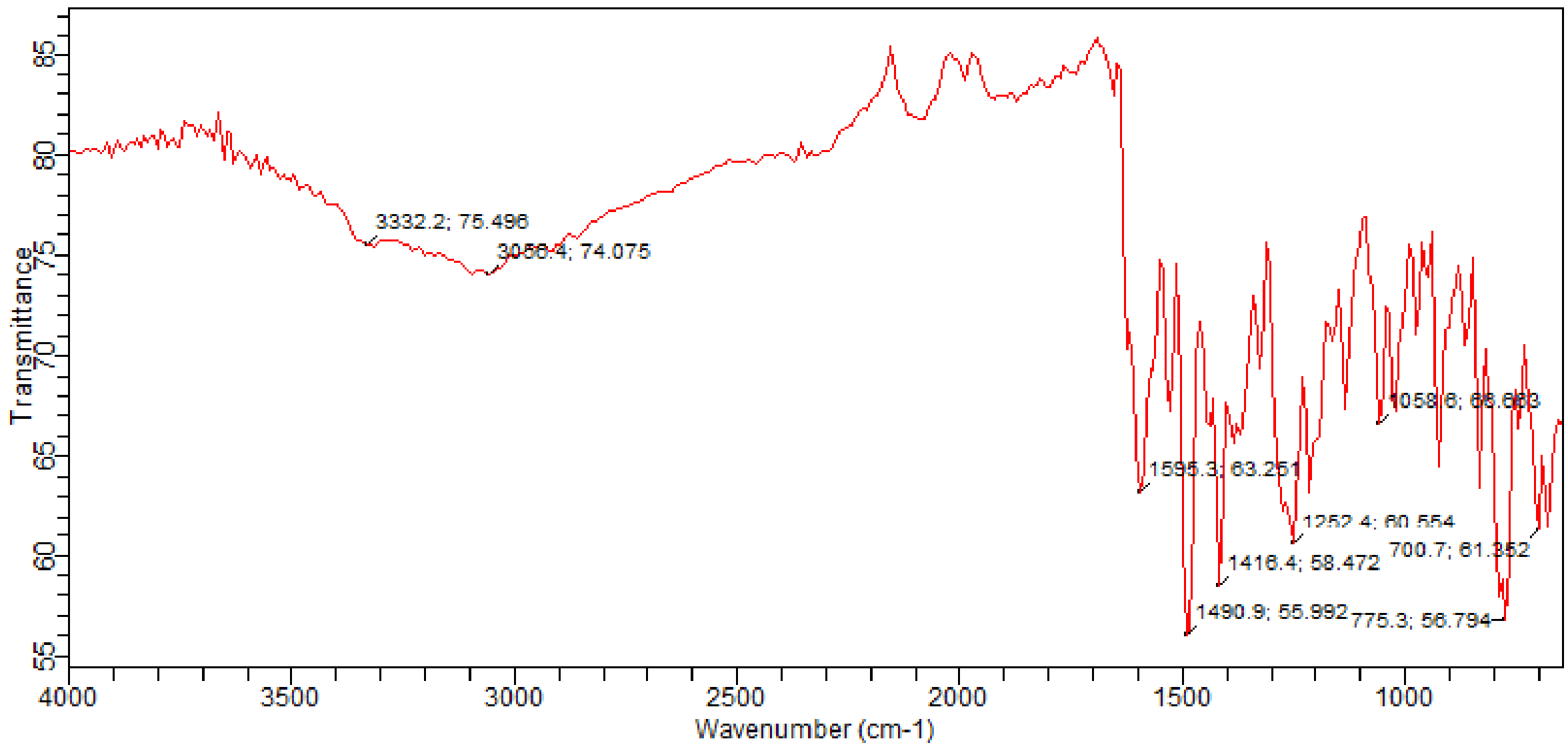

Compound 4e

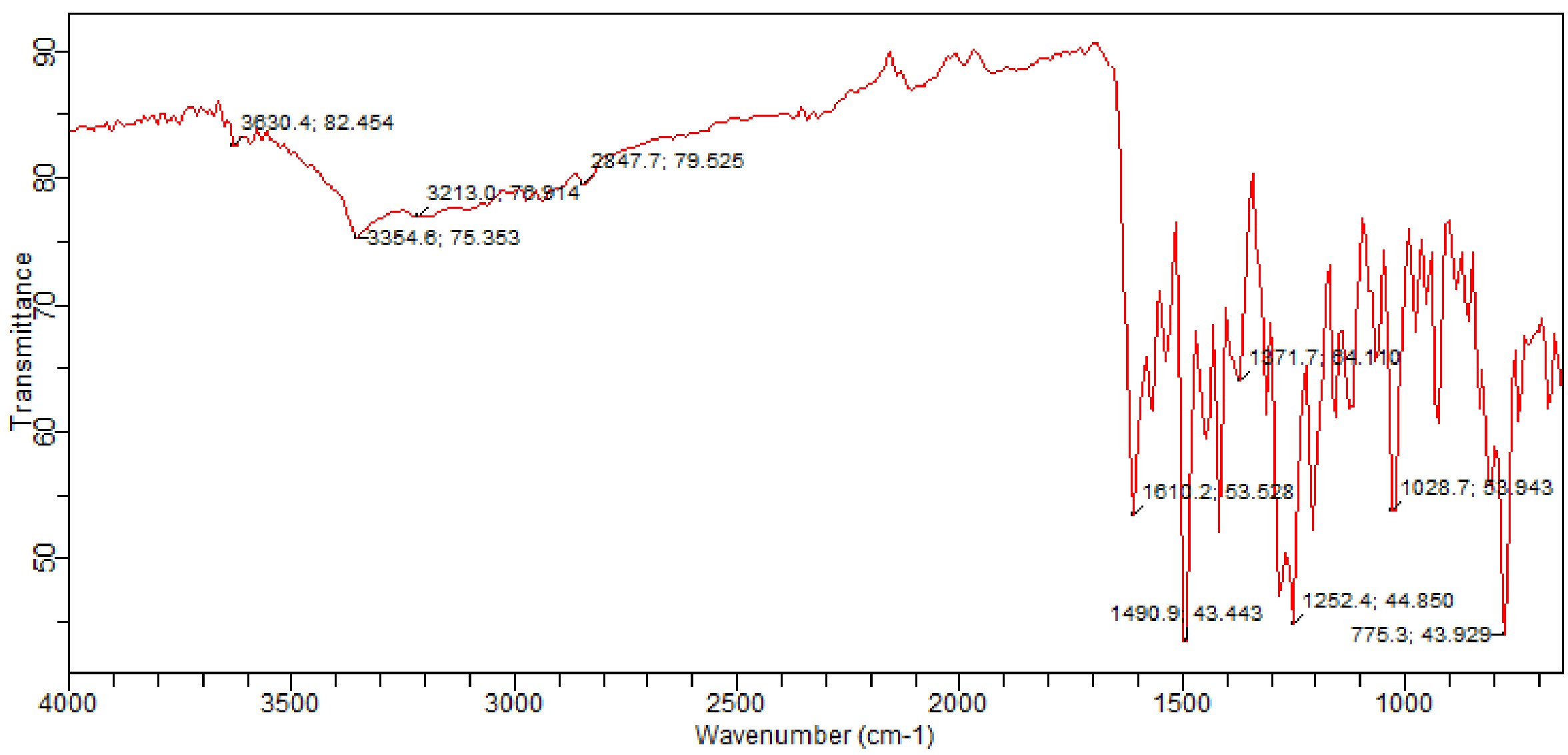

Compound 4f

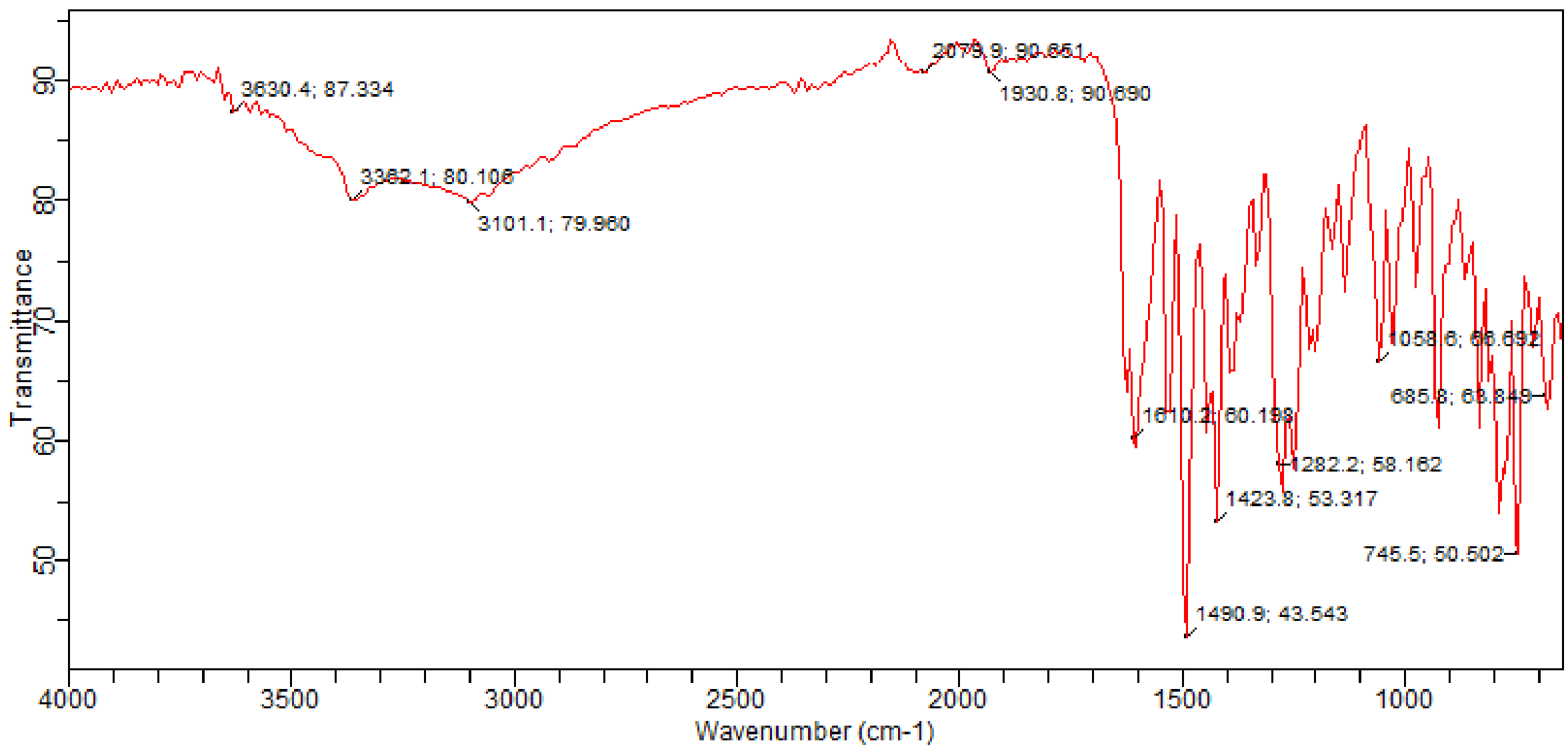

Compound 4g

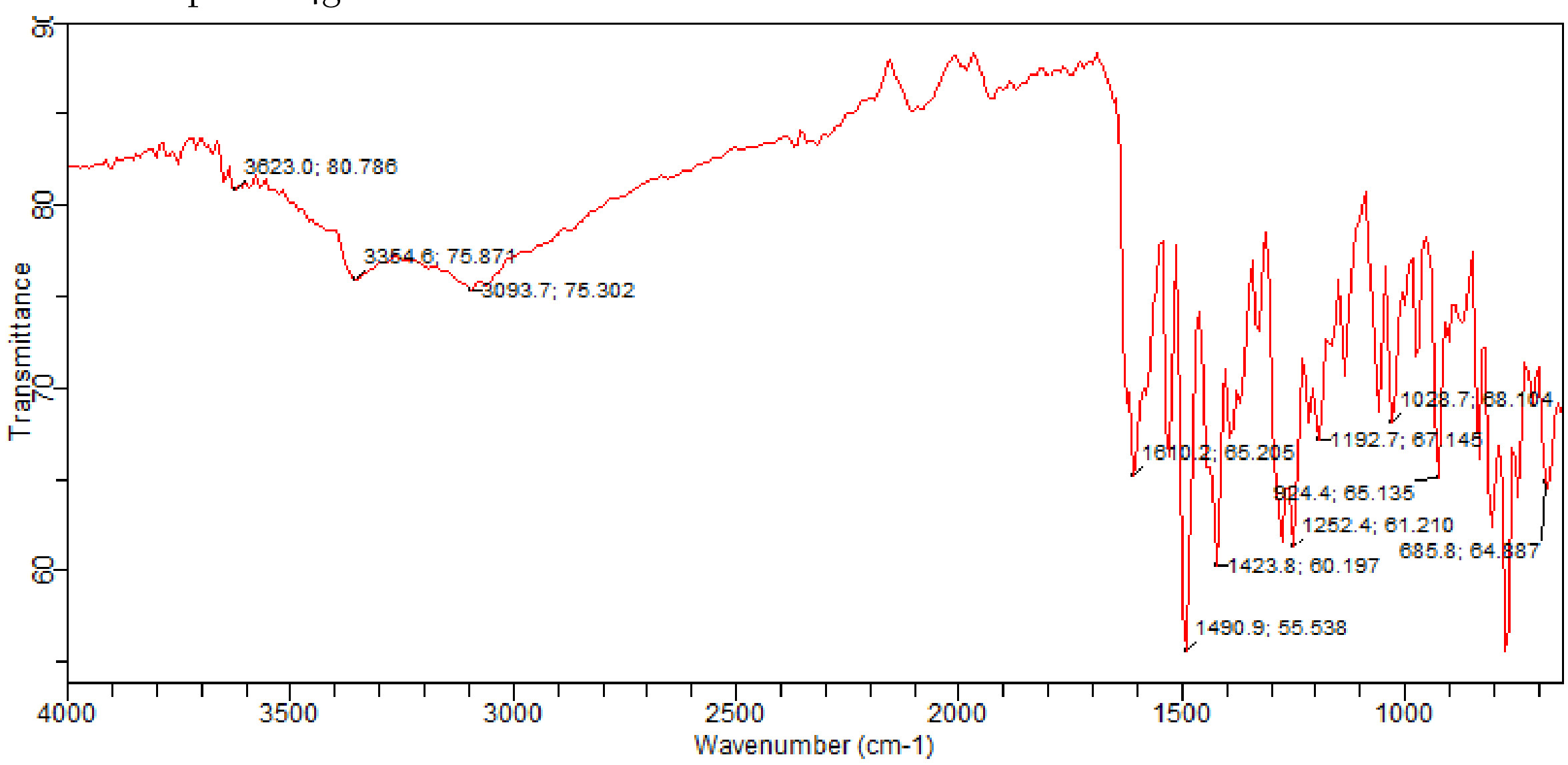

Compound 4h

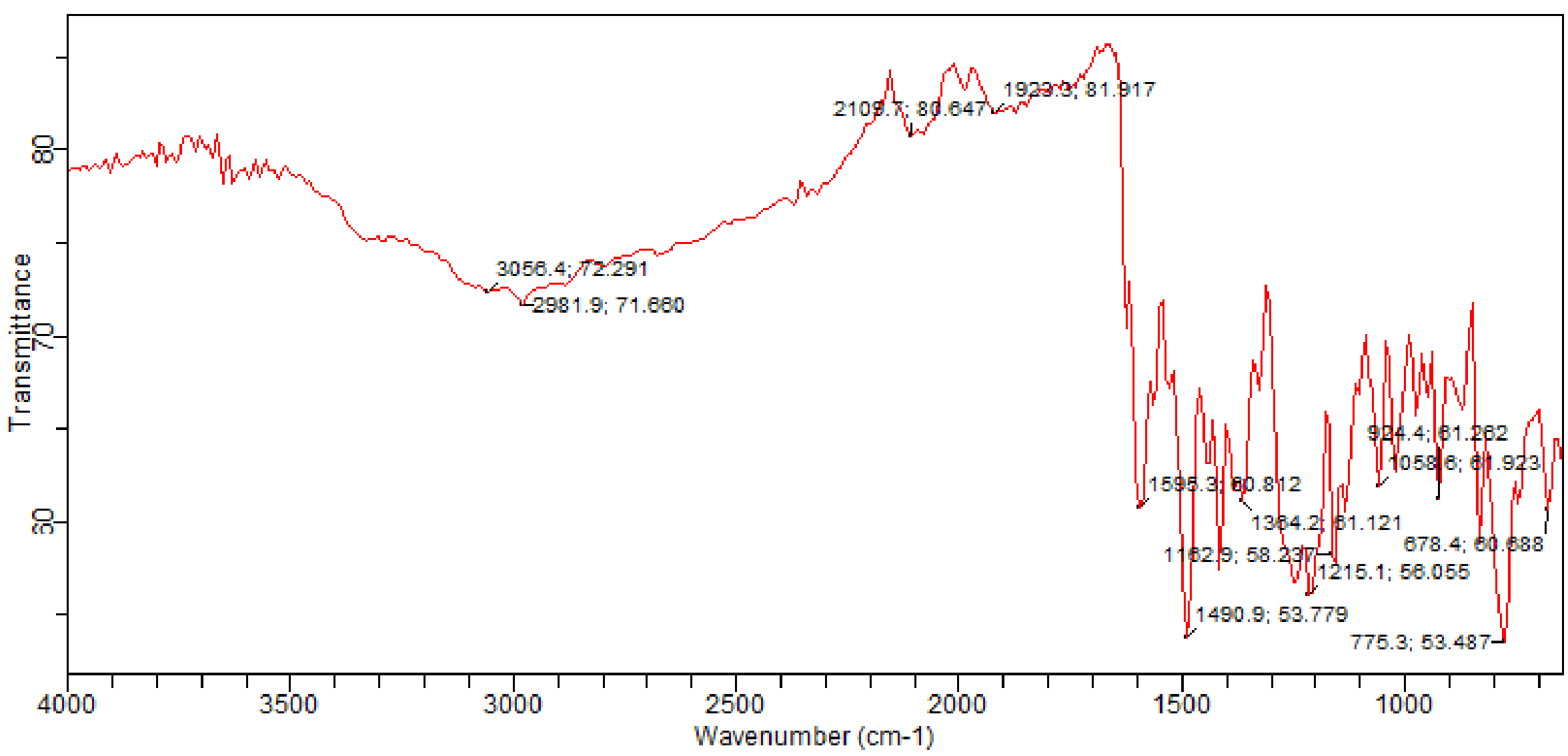

Supplementary Materials: 2. ^{1}H NMR spectra of synthesised compounds (4a to 4h).

Compound 4a: ^{1}H NMR (400 MHz, DMSO-d$_6$)

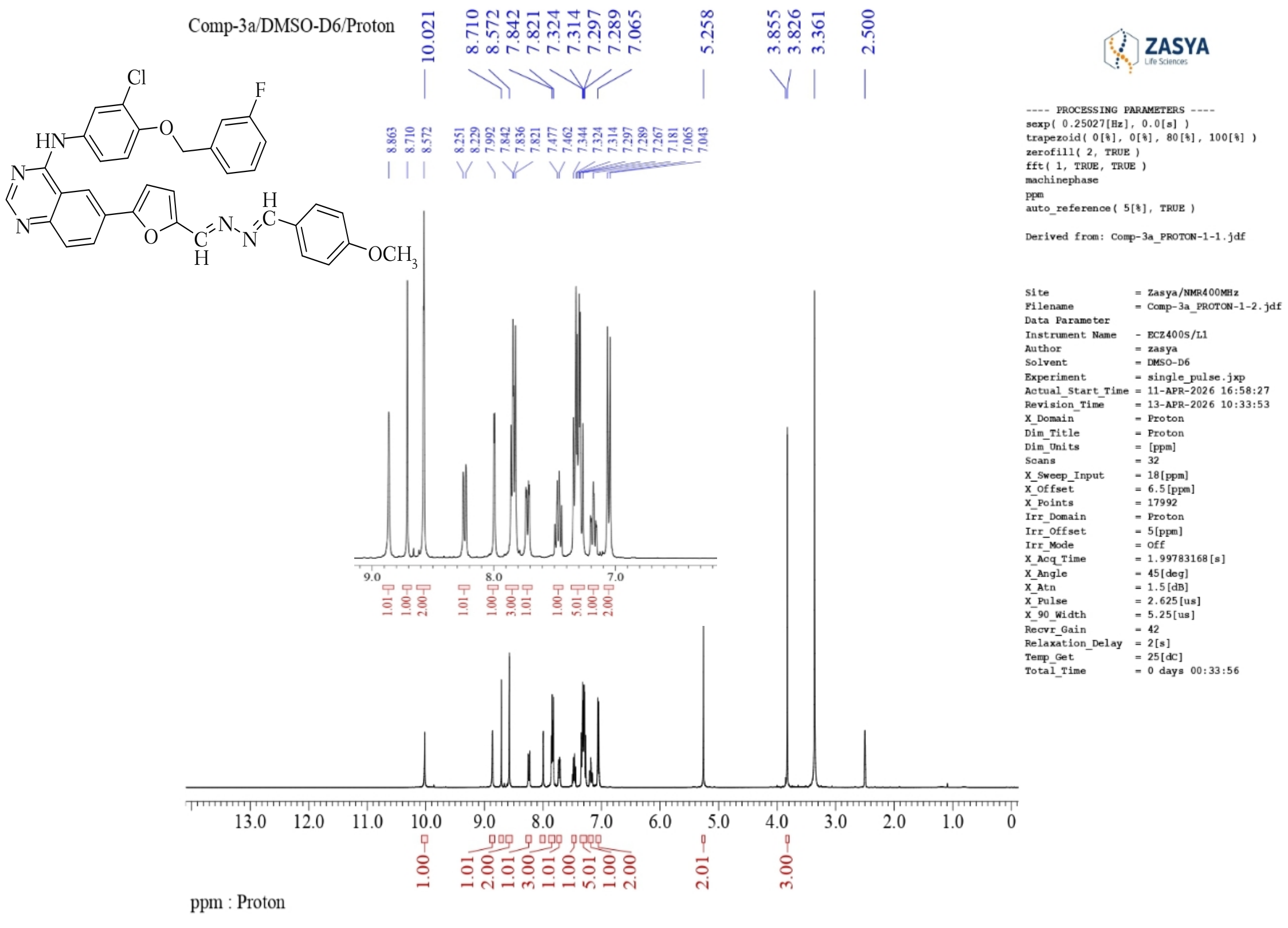

Compound 4b: ^{1}H NMR (400 MHz, DMSO-d$_6$)

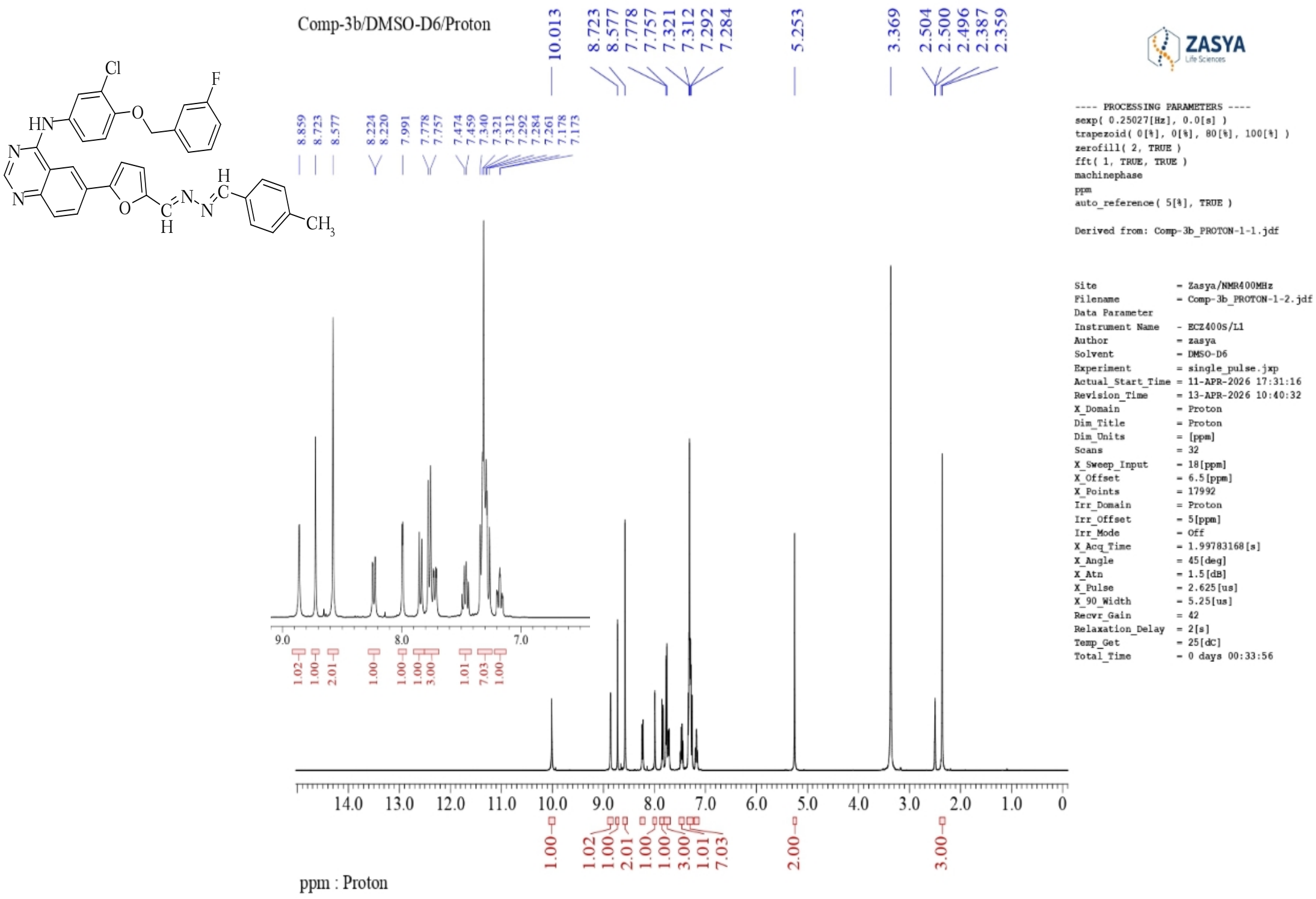

Compound 4c: ^{1}H NMR (400 MHz, DMSO-d_6)

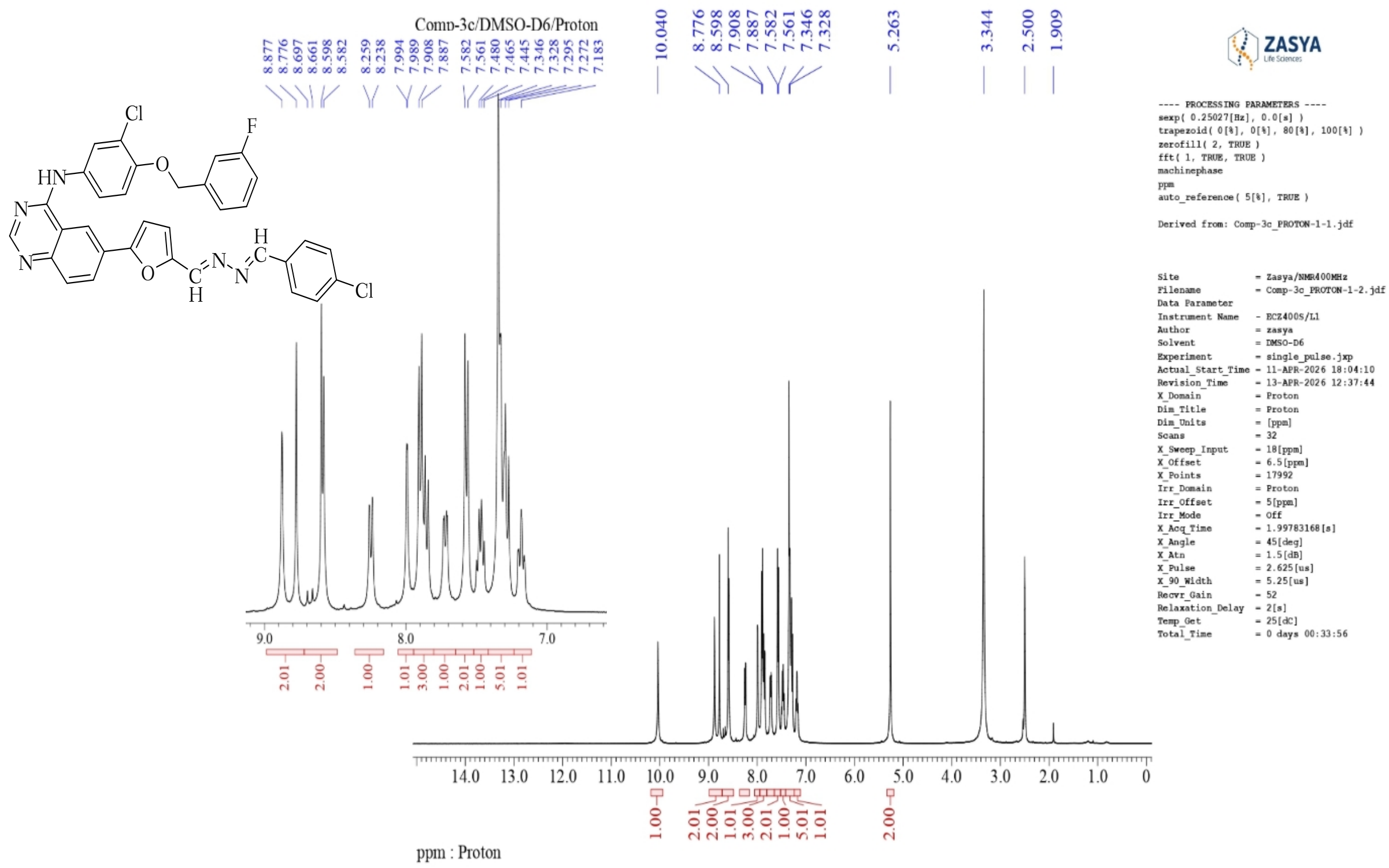

Compound 4d: ^{1}H NMR (400 MHz, DMSO-d_6)

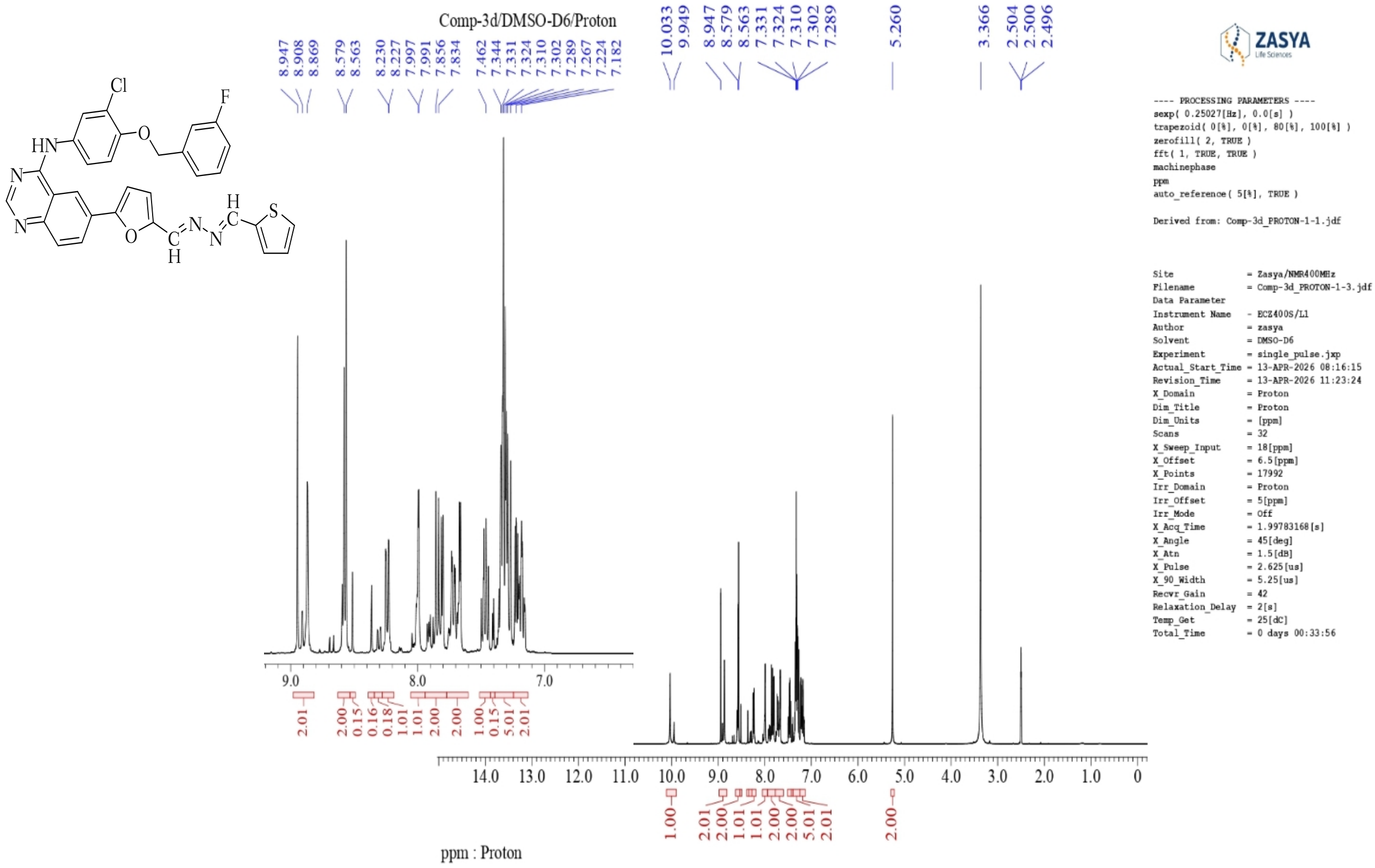

Compound 4e: ^{1}H NMR (400 MHz, DMSO-d$_6$)

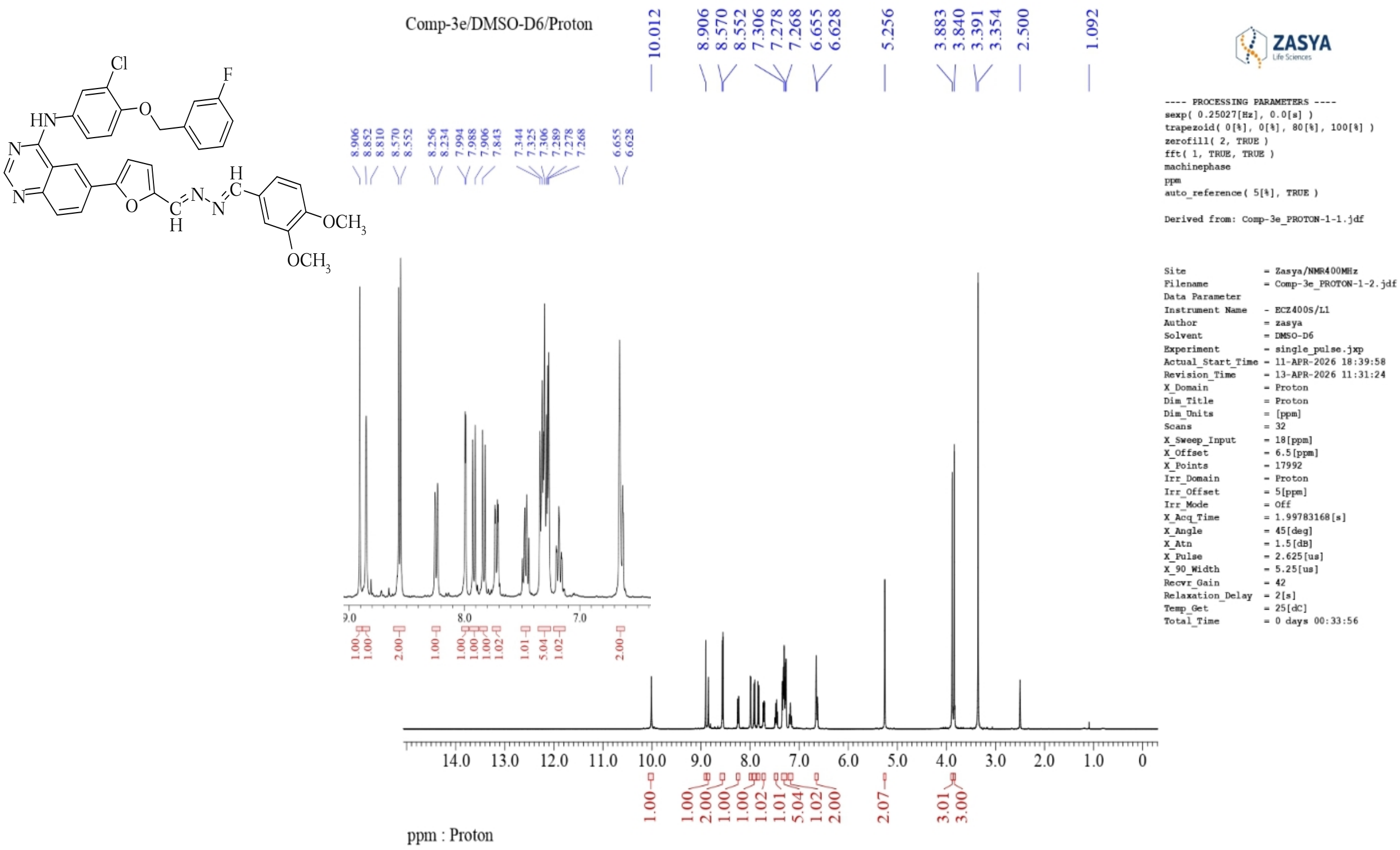

Compound 4f: ^{1}H NMR (400 MHz, DMSO-d$_6$)

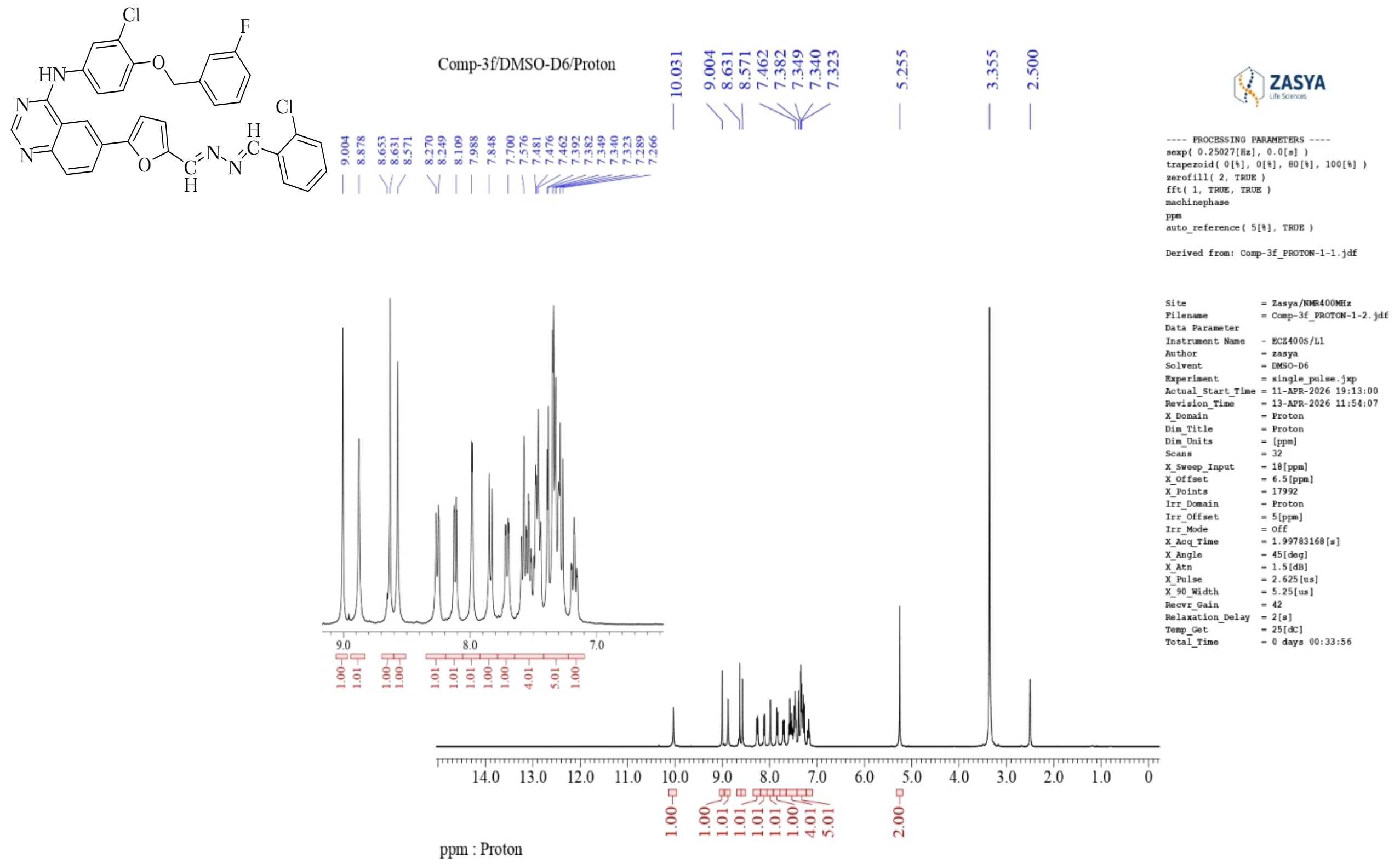

Compound 4g: ^{1}H NMR (400 MHz, DMSO-d_6)

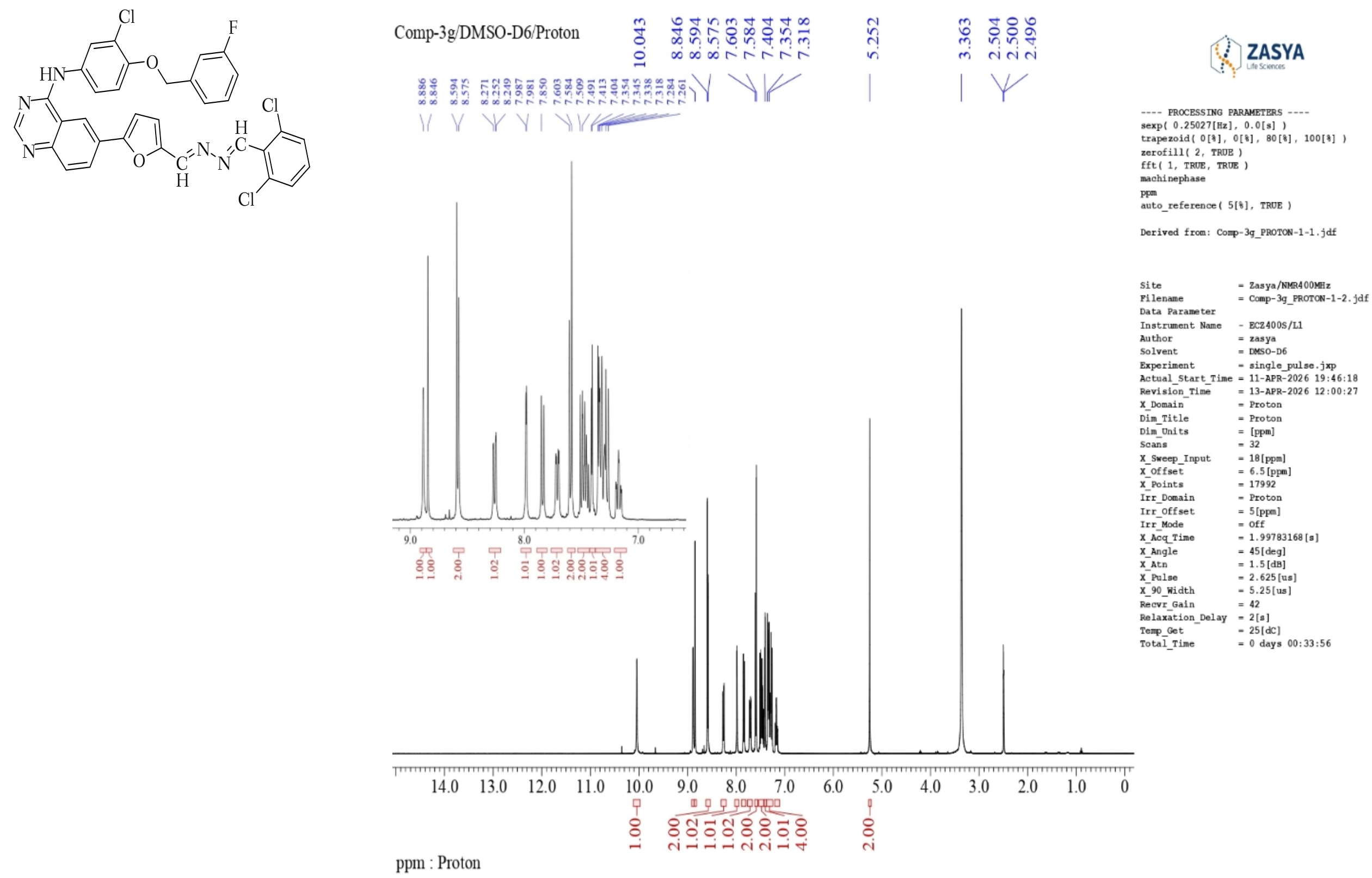

Compound 4h: ^{1}H NMR (400 MHz, DMSO-d_6)

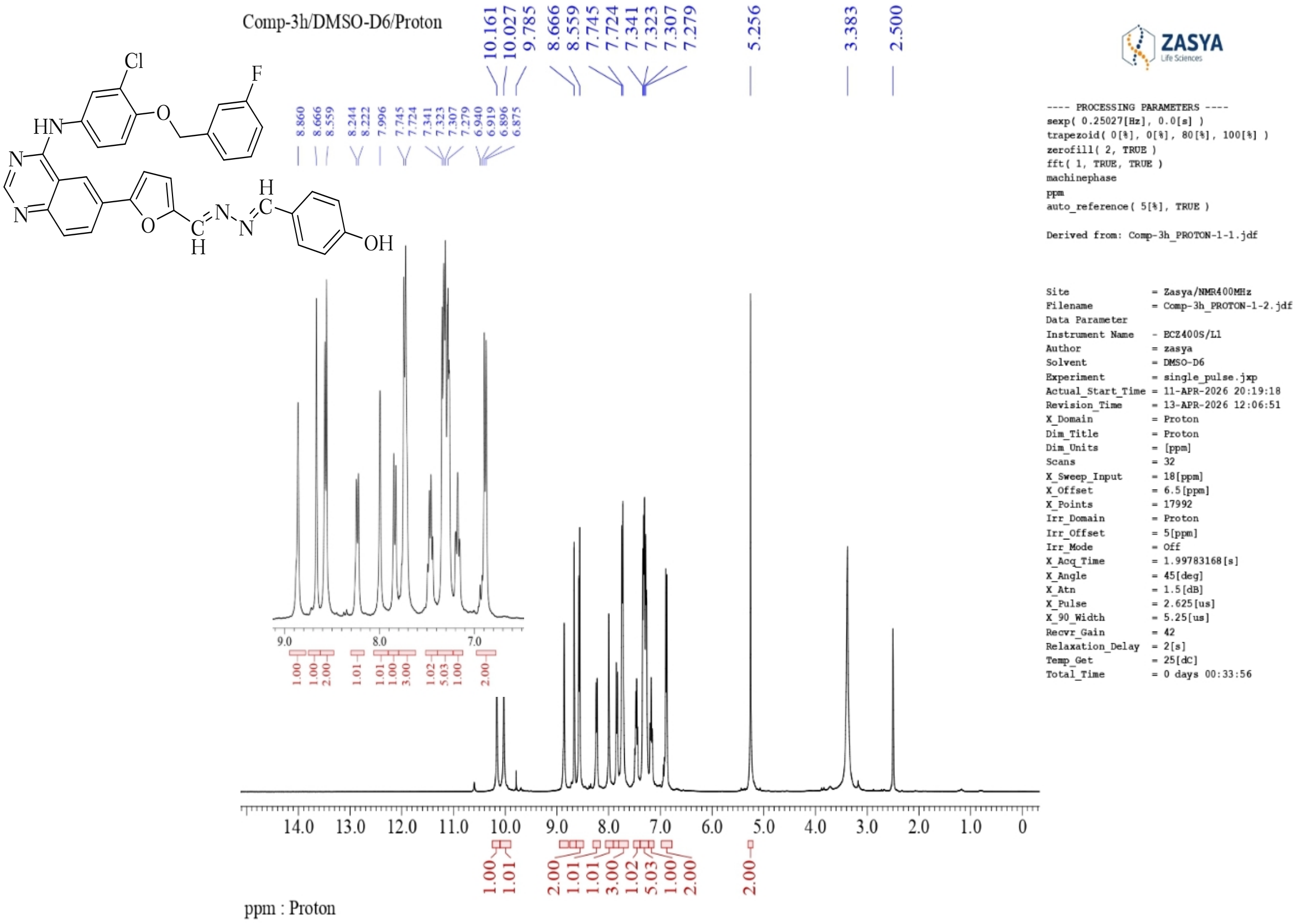

Supplementary Materials: 3 ^{13}C NMR spectra of synthesised compounds (4a to 4h)

Compound 4a: ^{13}C NMR (100 MHz, DMSO-d_6)

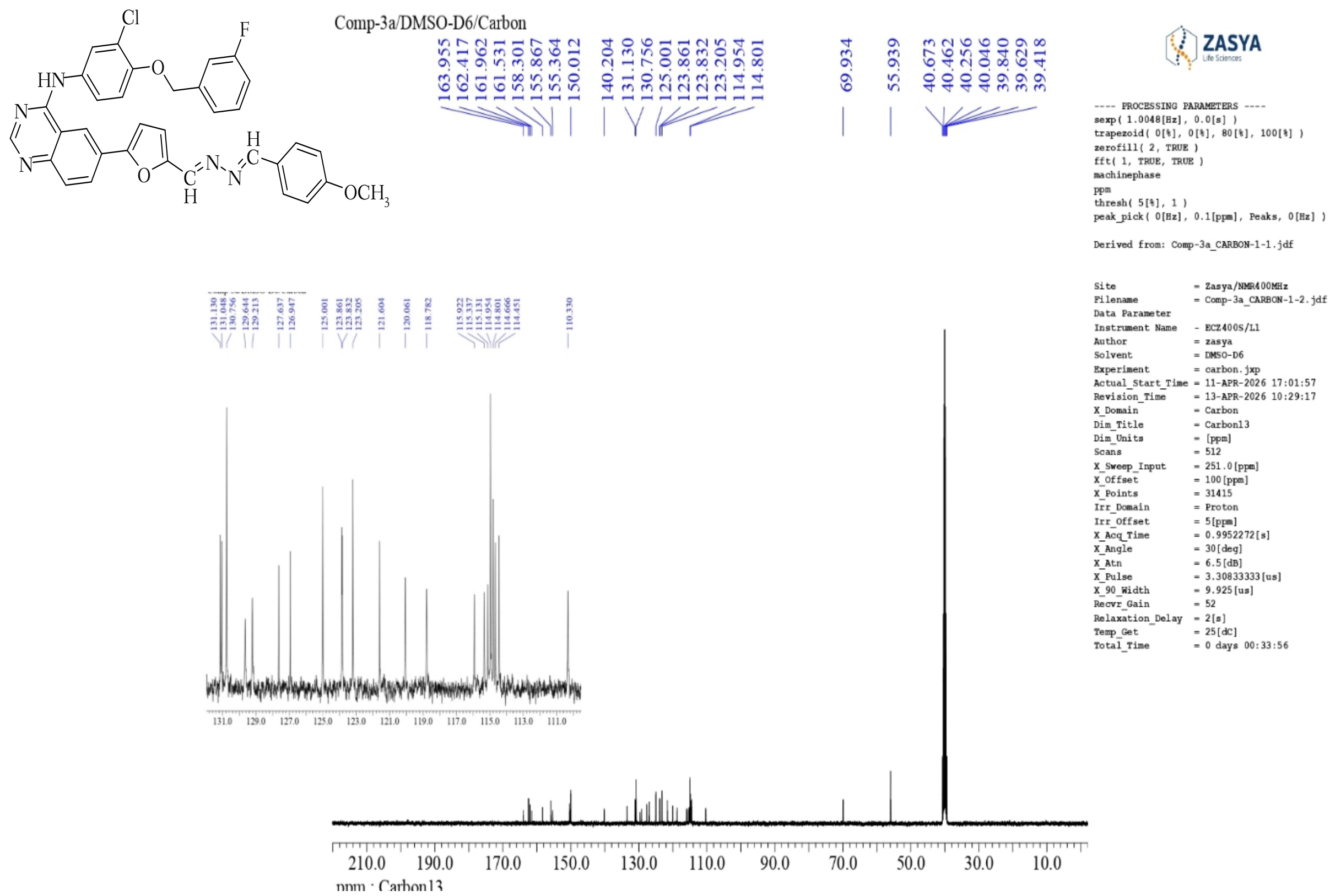

Compound 4b: ^{13}C NMR (100 MHz, DMSO-d_6)

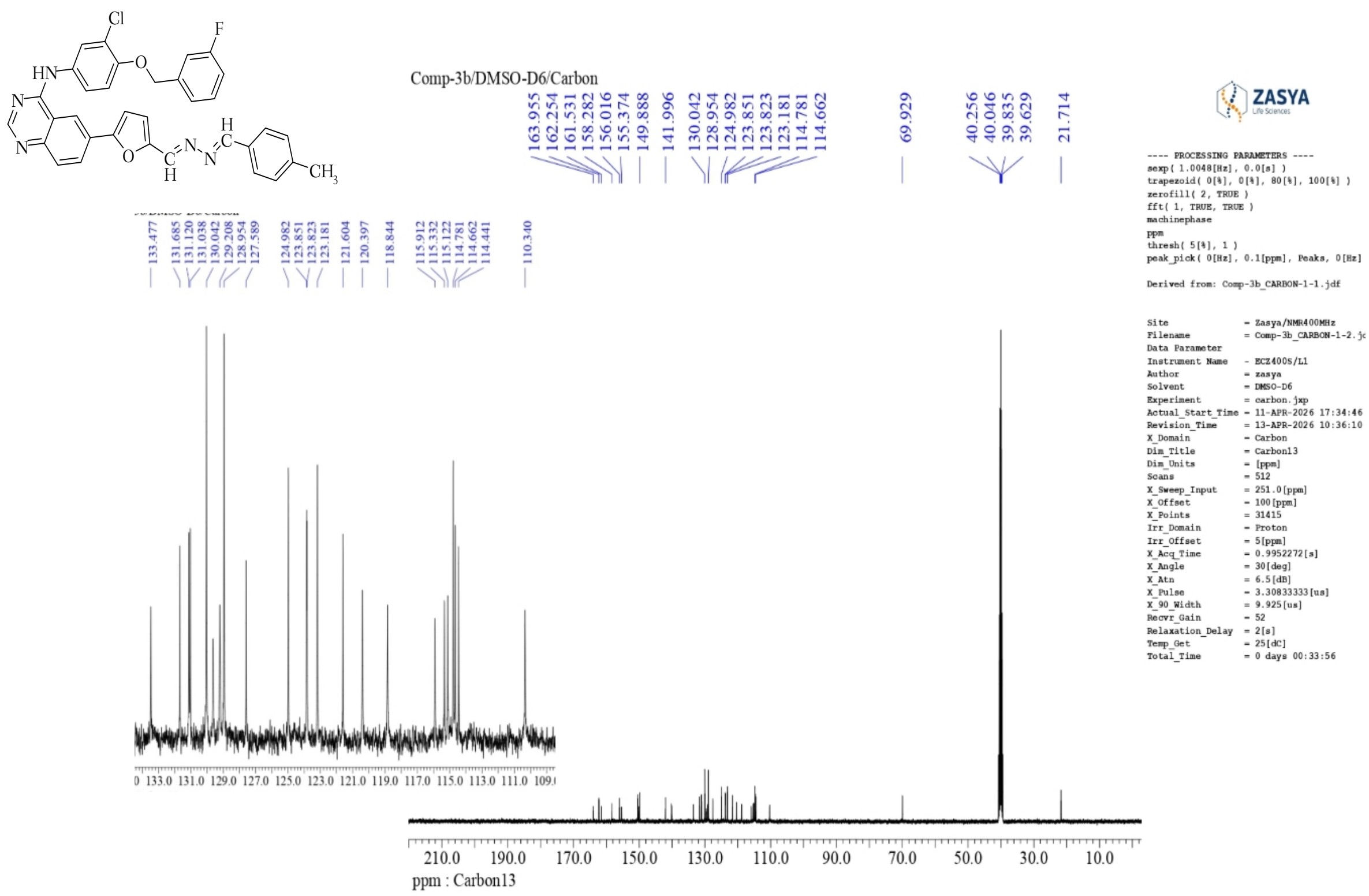

Compound 4c: ^{13}C NMR (100 MHz, DMSO-d_6)

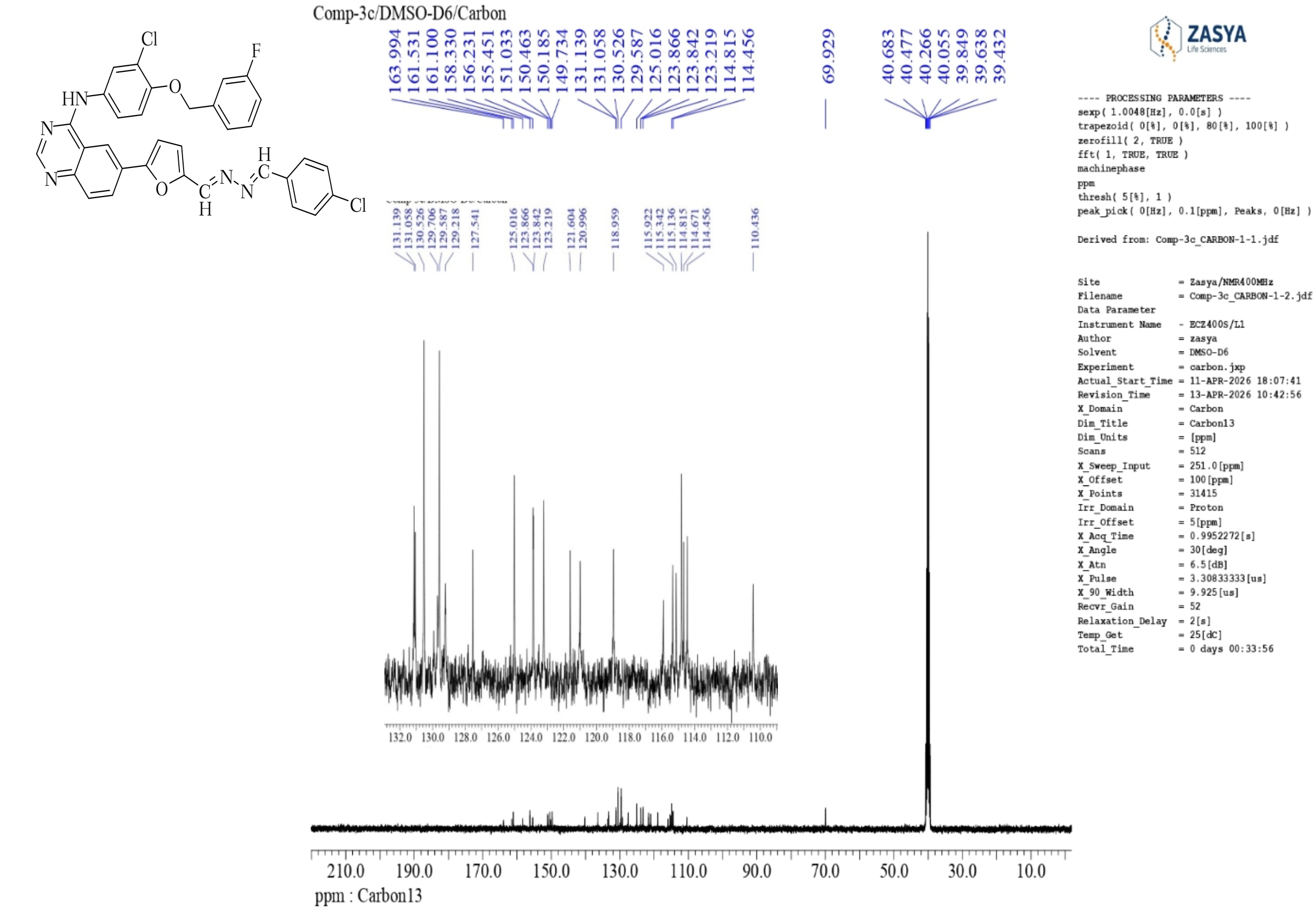

Compound 4d: ^{13}C NMR (100 MHz, DMSO-d_6)

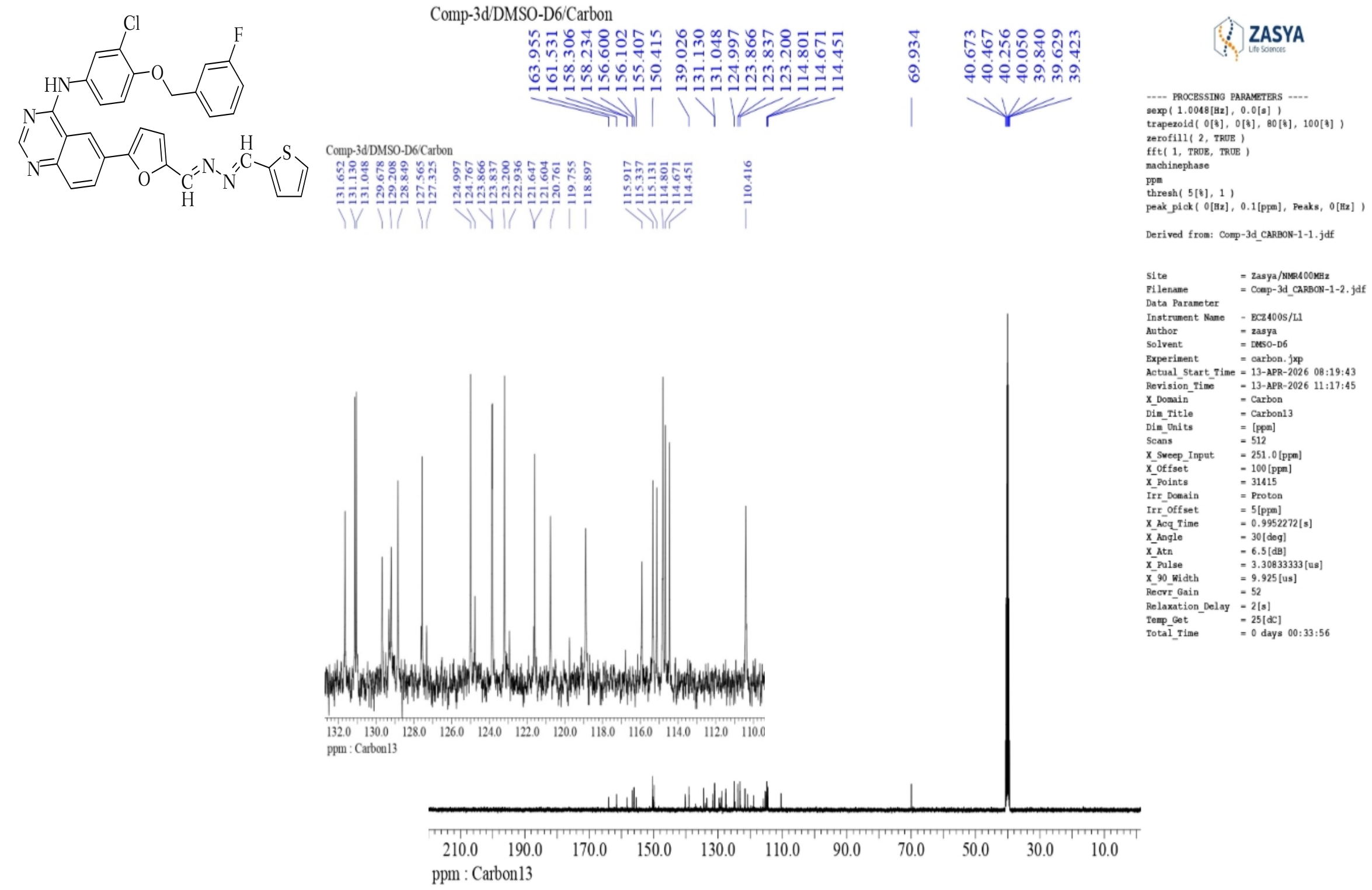

Compound 4e: ^{13}C NMR (100 MHz, DMSO-d_6)

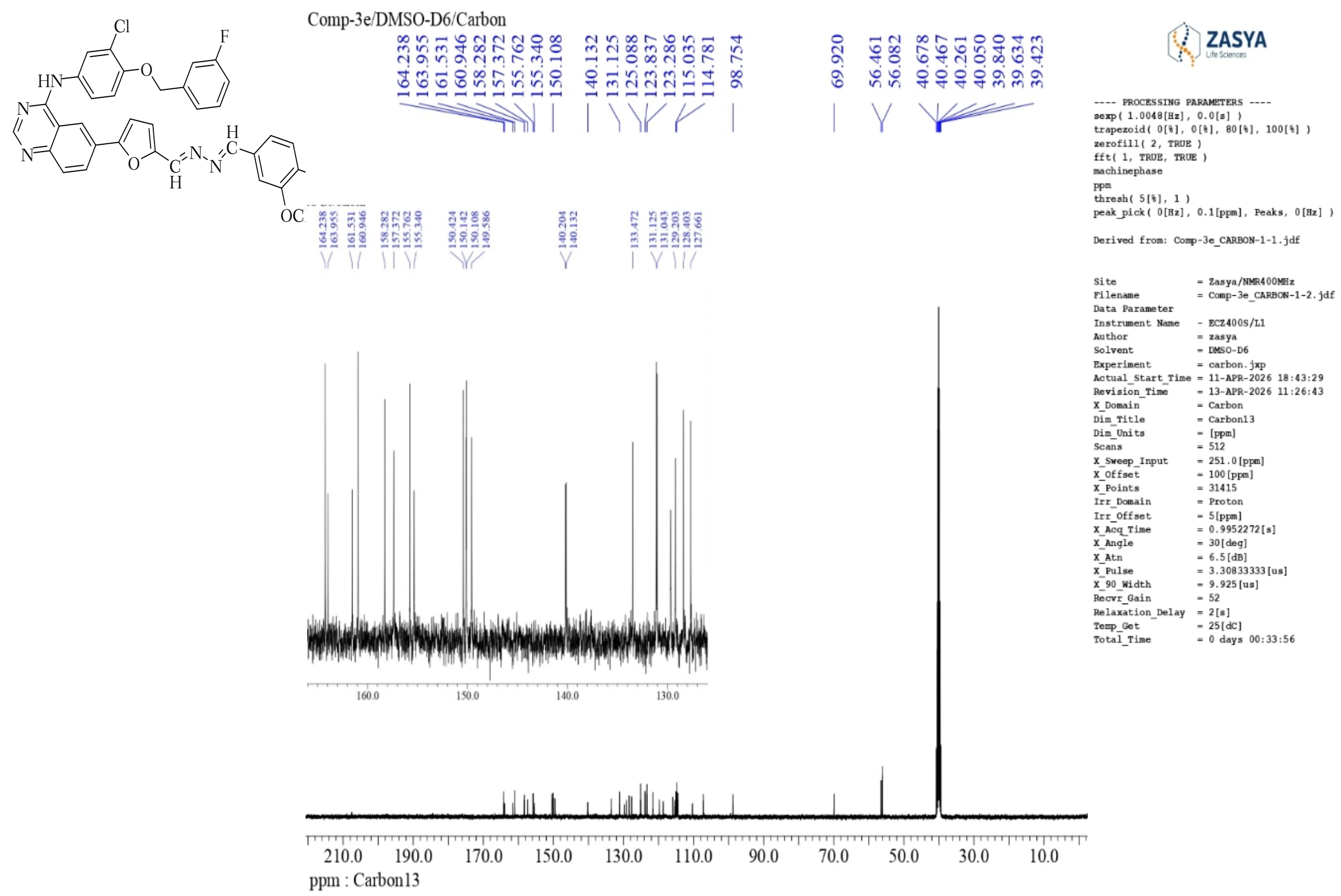

Compound 4f: ^{13}C NMR (100 MHz, DMSO-d_6)

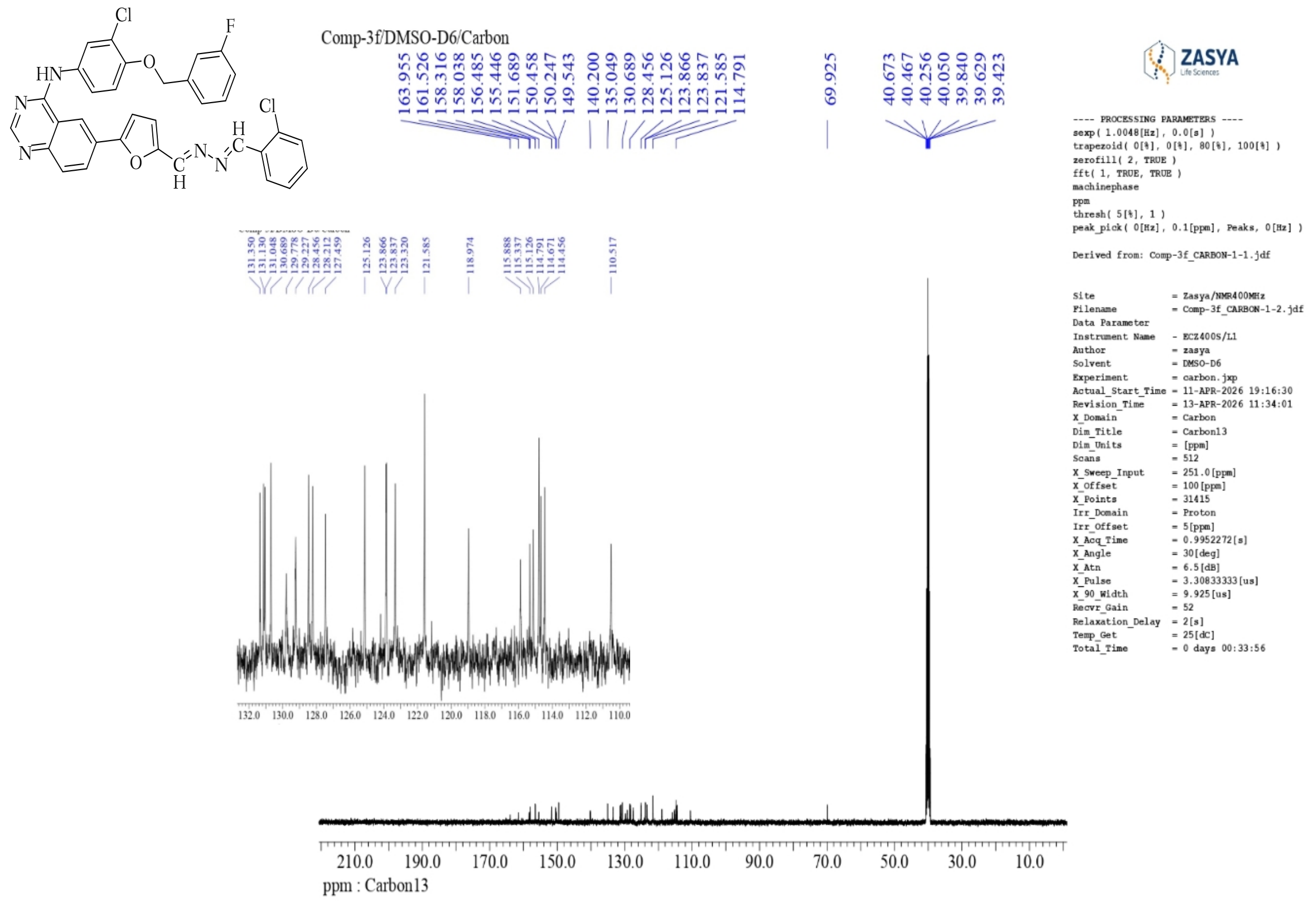

Compound 4g: ^{13}C NMR (100 MHz, DMSO-d_6)

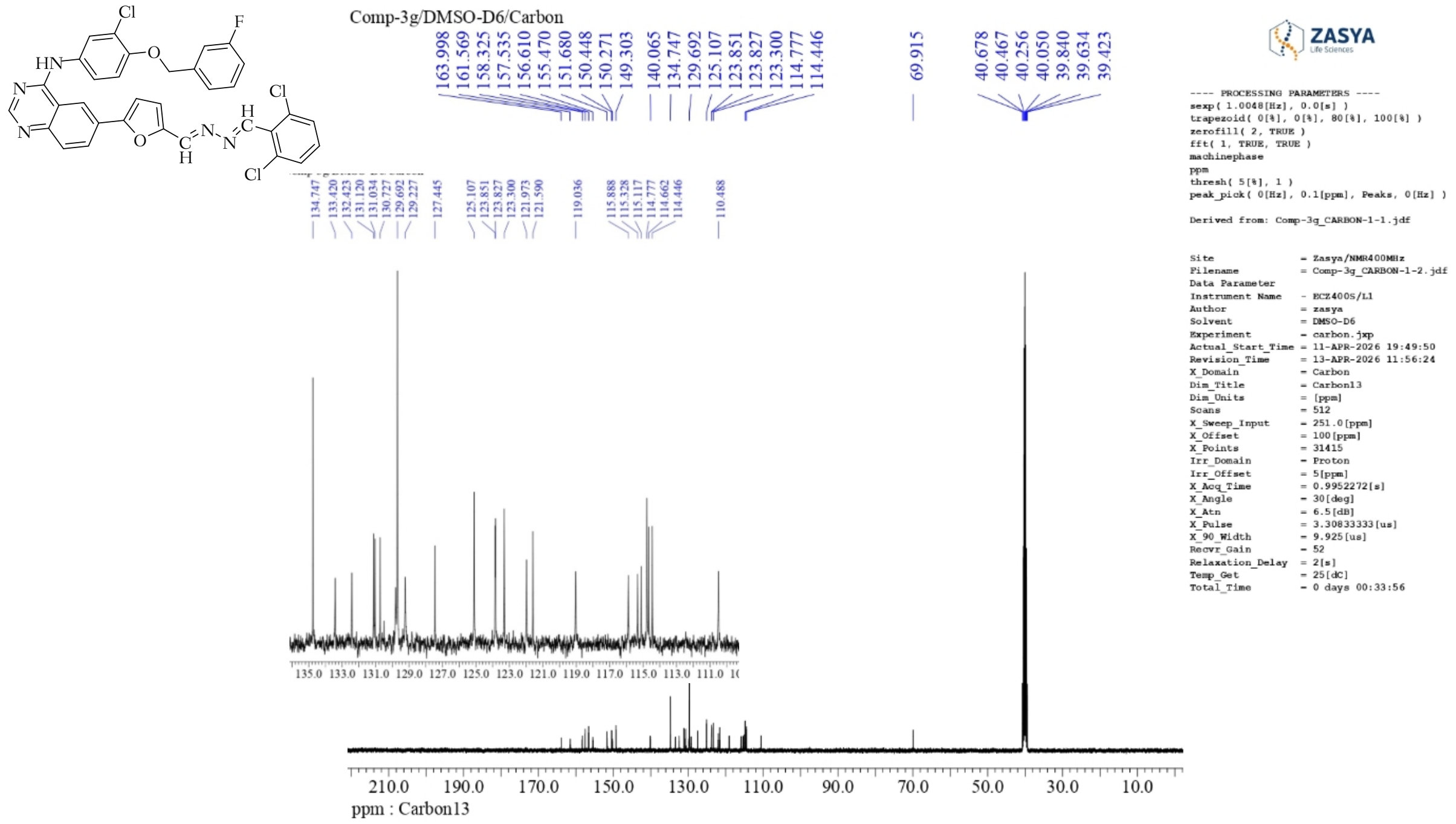

Compound 4h: ^{13}C NMR (100 MHz, DMSO-d_6)

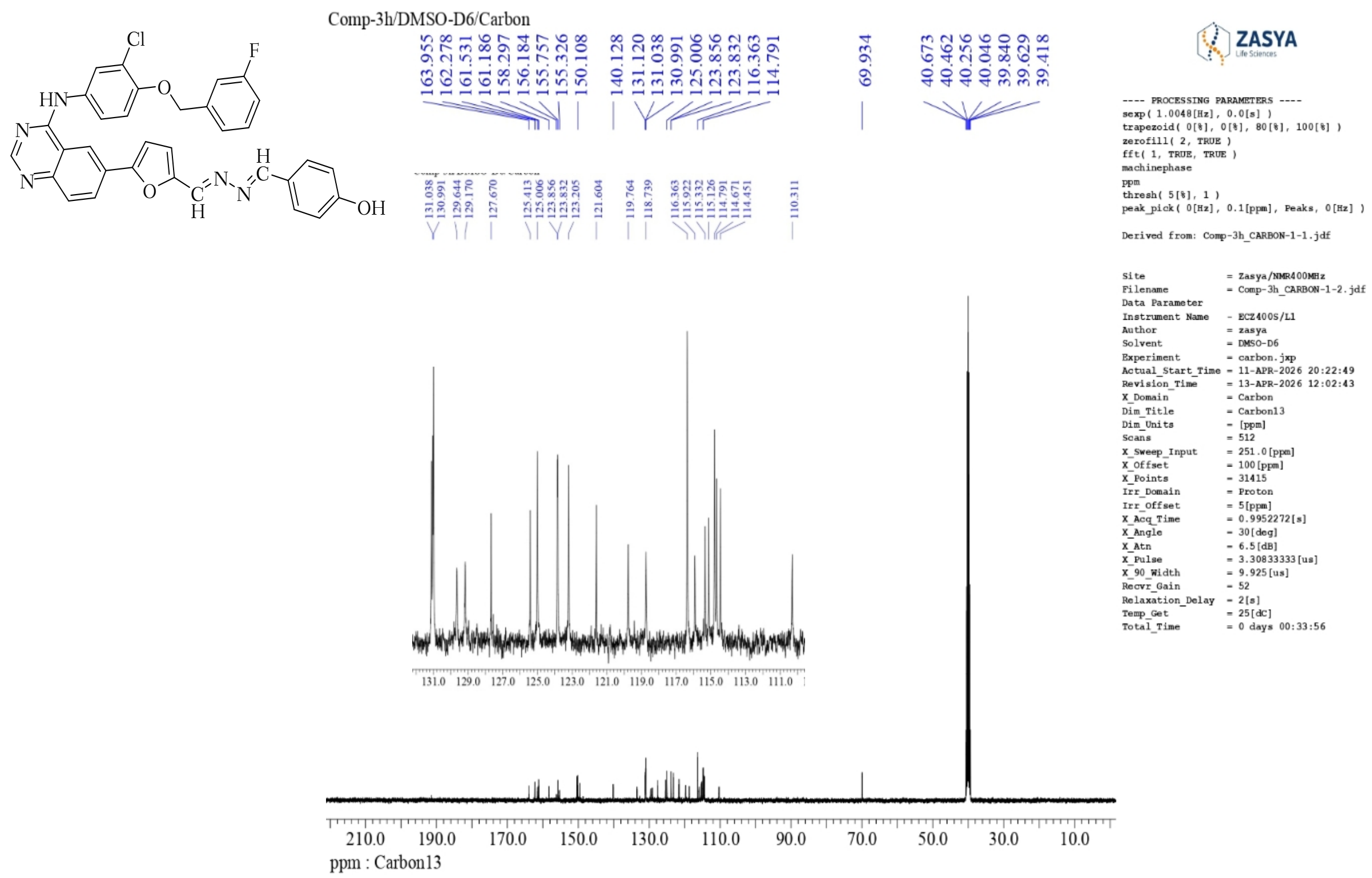

Supplementary Materials: 4 Mass spectra of synthesised compounds (4a to 4h)

Compound 4a

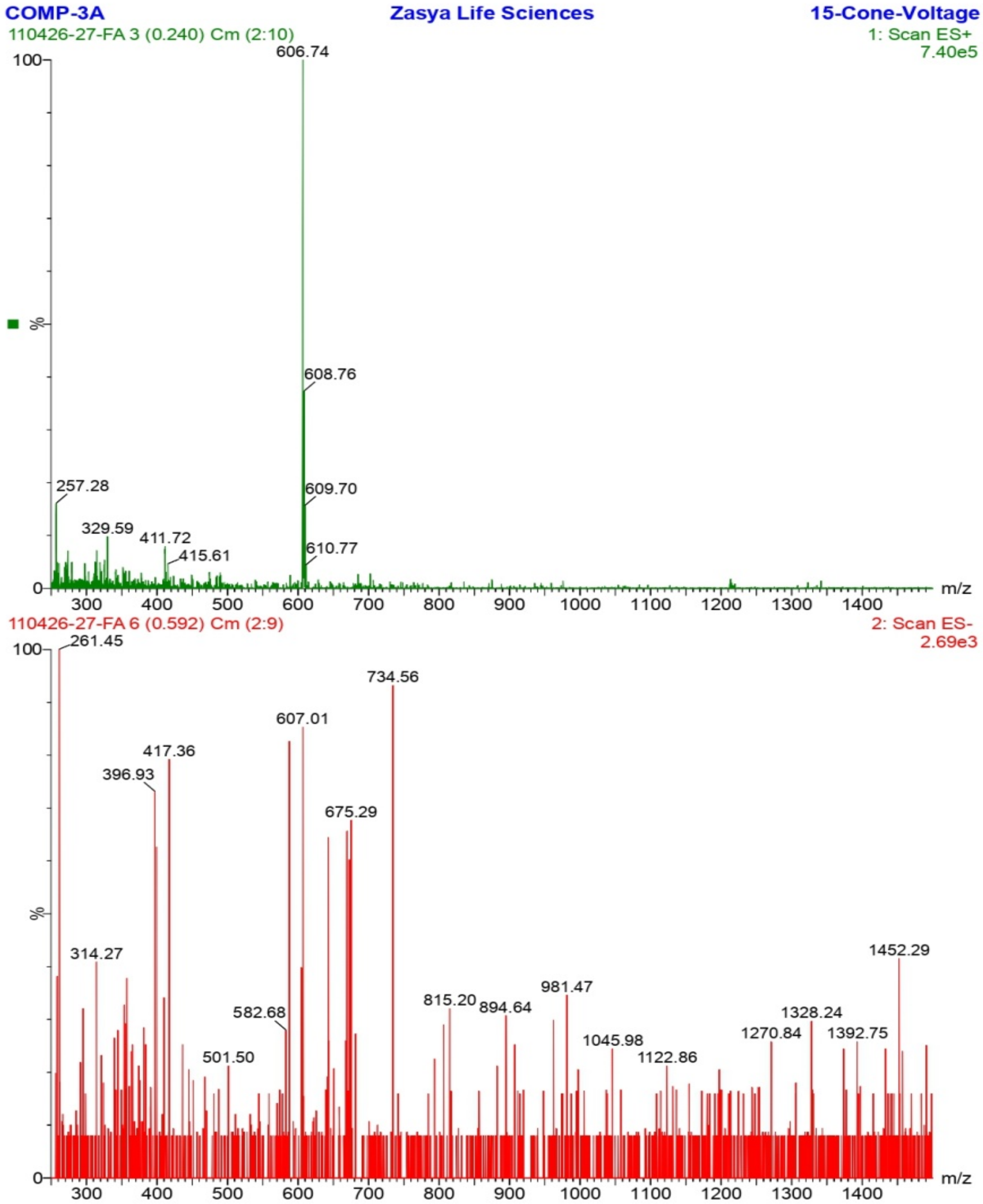
COMP-3A
Zasya Life Sciences
15-Cone-Voltage
110426-27-FA 3 (0.240) Cm (2:10)
1: Scan ES+
7.40e5
606.74
608.76
609.70
610.77
257.28
329.59
411.72
415.61
110426-27-FA 6 (0.592) Cm (2:9)
2: Scan ES-
2.69e3
261.45
734.56
607.01
417.36
396.93
675.29
314.27
1452.29
981.47
815.20
894.64
582.68
1328.24
1270.84
1392.75
1045.98
501.50
1122.86
m/z

Compound 4b

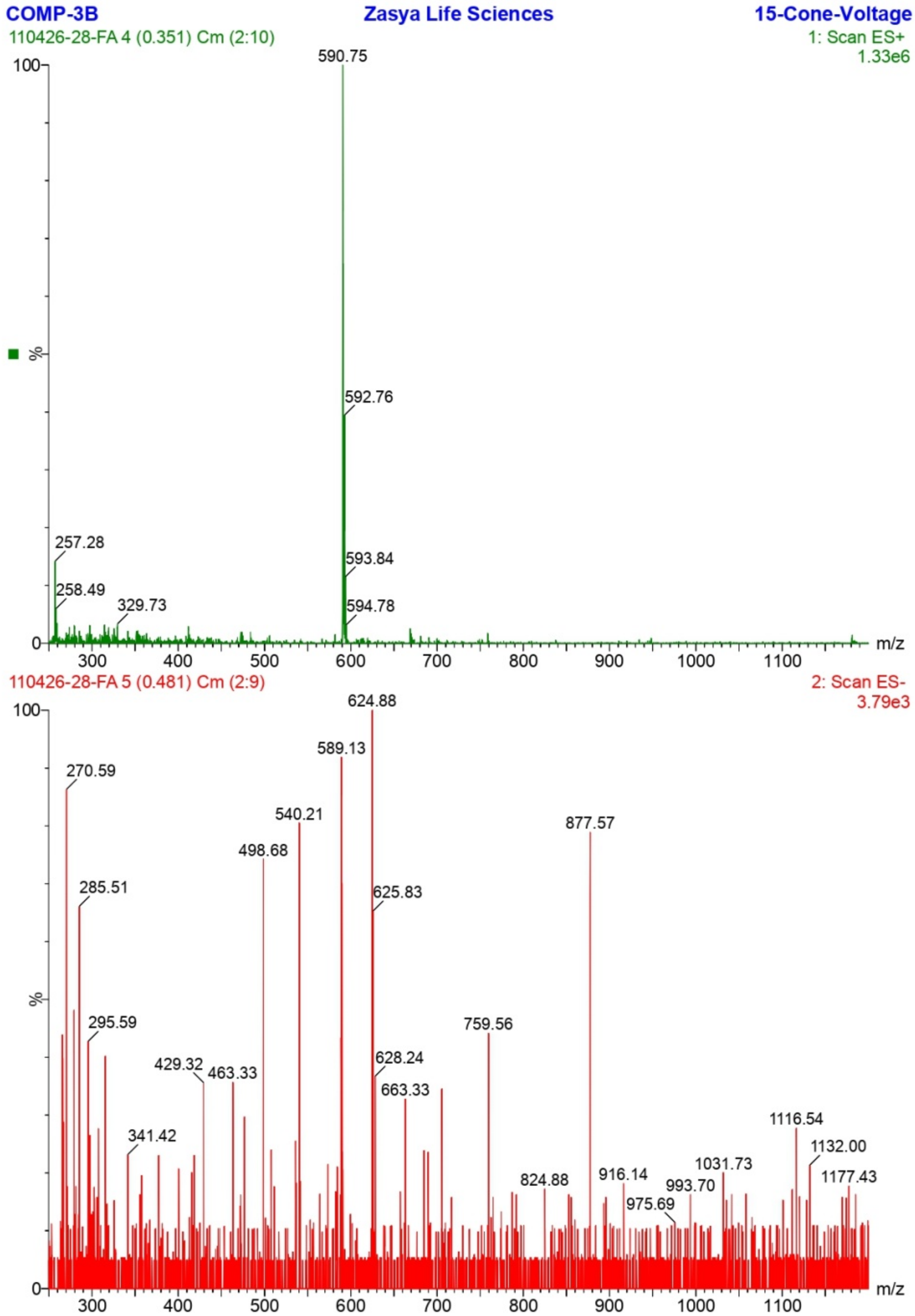
COMP-3B
Zasya Life Sciences
15-Cone-Voltage
110426-28-FA 4 (0.351) Cm (2:10)
1: Scan ES+
1.33e6
590.75
592.76
257.28
593.84
258.49
329.73
594.78
100
%
0
300
400
500
600
700
800
900
1000
1100
m/z
110426-28-FA 5 (0.481) Cm (2:9)
2: Scan ES-
3.79e3
624.88
589.13
270.59
540.21
877.57
498.68
285.51
625.83
295.59
759.56
429.32
463.33
628.24
663.33
341.42
1116.54
1132.00
1031.73
824.88
916.14
1177.43
993.70
975.69
100
%
0
300
400
500
600
700
800
900
1000
1100
m/z

Compound 4c

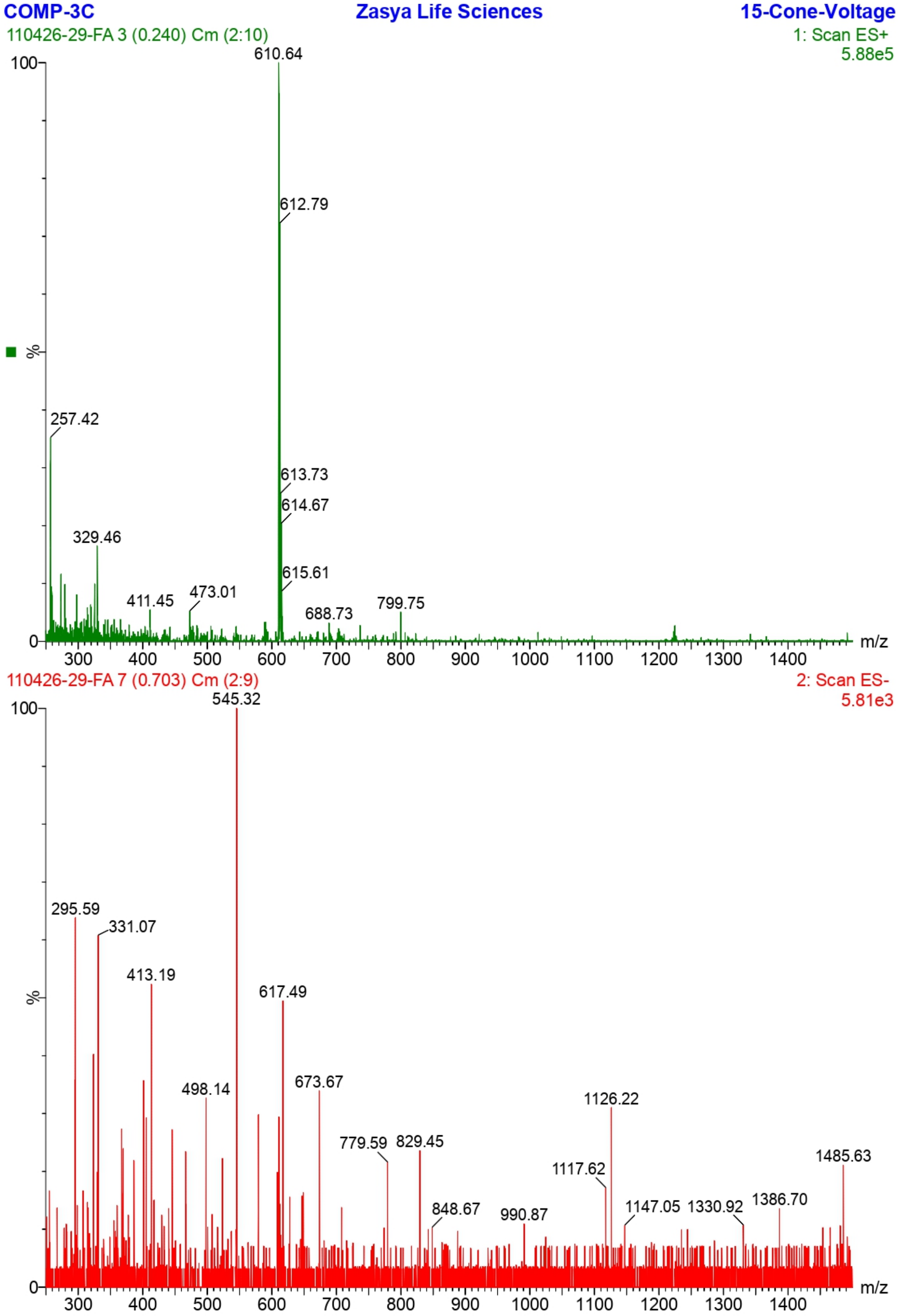

COMP-3C
Zasya Life Sciences
15-Cone-Voltage
110426-29-FA 3 (0.240) Cm (2:10)
1: Scan ES+
5.88e5
610.64
612.79
257.42
613.73
614.67
329.46
615.61
411.45
473.01
688.73
799.75
%
m/z
110426-29-FA 7 (0.703) Cm (2:9)
2: Scan ES-
5.81e3
545.32
295.59
331.07
413.19
617.49
498.14
673.67
1126.22
779.59
829.45
1117.62
1485.63
848.67
990.87
1147.05
1330.92
1386.70
%
m/z

Compound 4d

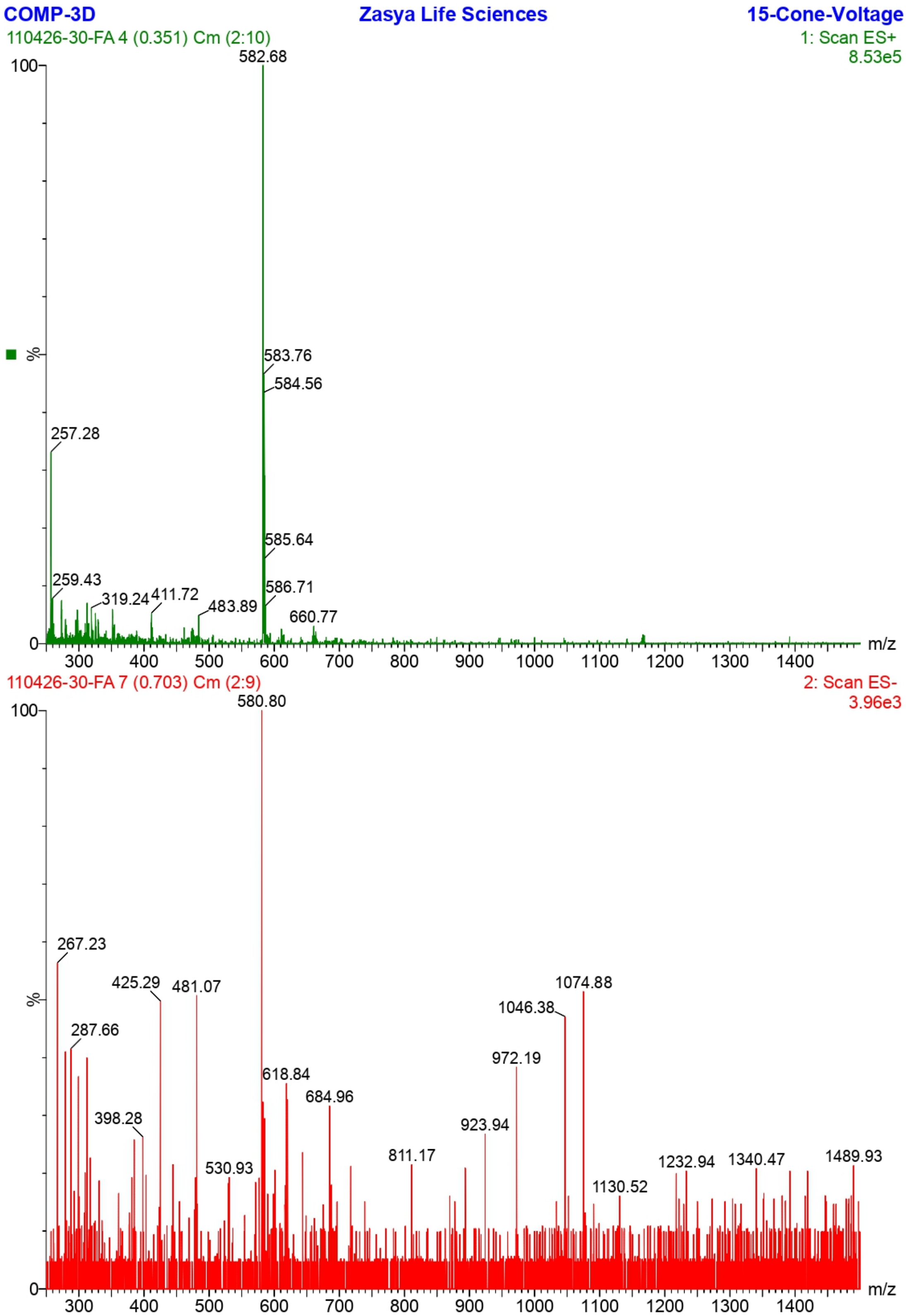
COMP-3D
Zasya Life Sciences
15-Cone-Voltage
110426-30-FA 4 (0.351) Cm (2:10)
1: Scan ES+
8.53e5
582.68
583.76
584.56
257.28
585.64
259.43
319.24
411.72
483.89
586.71
660.77
%
m/z
110426-30-FA 7 (0.703) Cm (2:9)
2: Scan ES-
3.96e3
580.80
267.23
425.29
481.07
1074.88
1046.38
287.66
972.19
618.84
684.96
398.28
923.94
811.17
530.93
1232.94
1340.47
1489.93
1130.52
m/z

Compound 4e

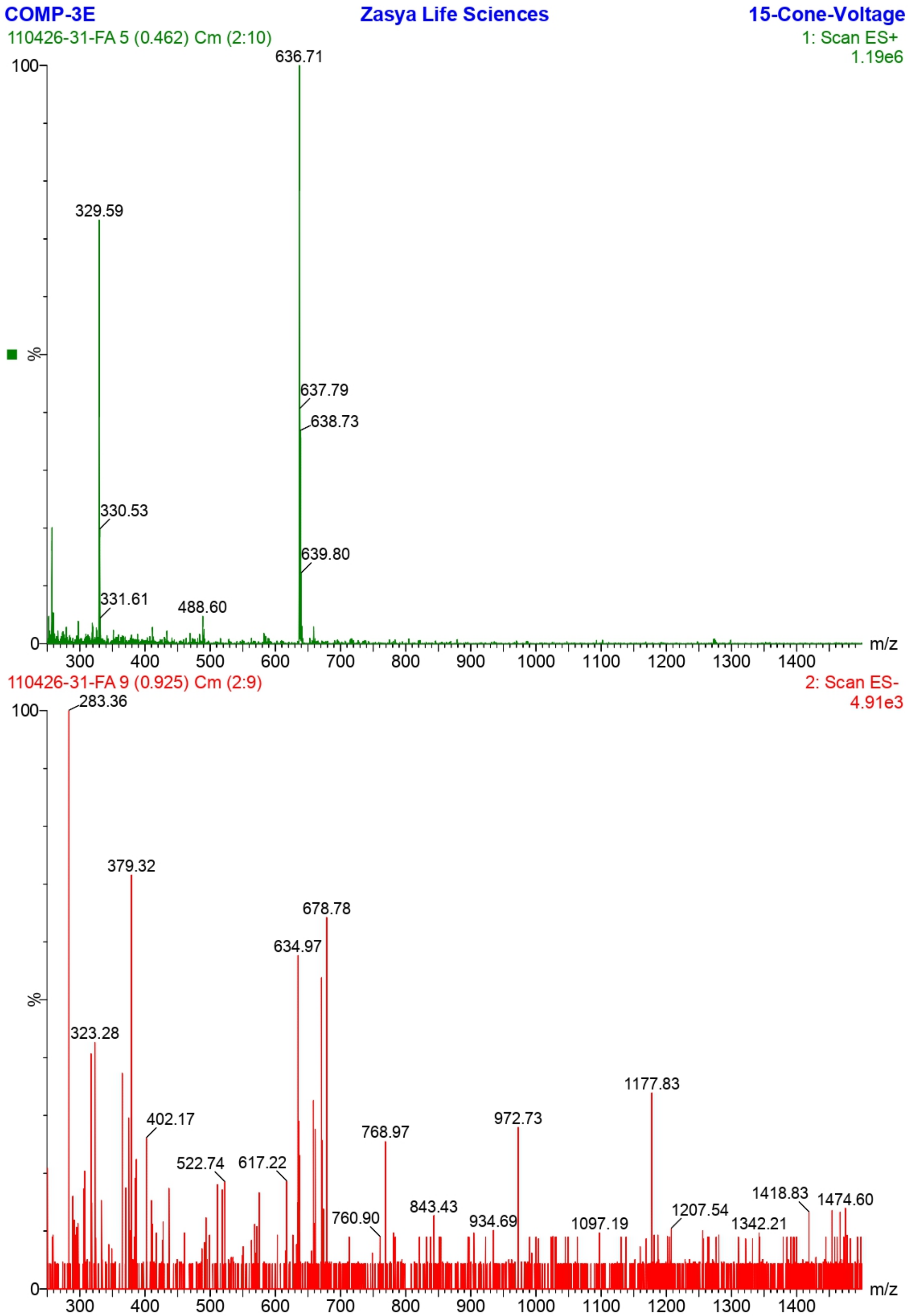
COMP-3E
Zasya Life Sciences
15-Cone-Voltage
110426-31-FA 5 (0.462) Cm (2:10)
1: Scan ES+
1.19e6
636.71
329.59
637.79
638.73
330.53
639.80
331.61
488.60
%
100
0
300
400
500
600
700
800
900
1000
1100
1200
1300
1400
m/z
110426-31-FA 9 (0.925) Cm (2:9)
2: Scan ES-
4.91e3
283.36
379.32
678.78
634.97
323.28
1177.83
402.17
972.73
768.97
522.74
617.22
1418.83
1474.60
843.43
1207.54
760.90
934.69
1097.19
1342.21

Compound 4f

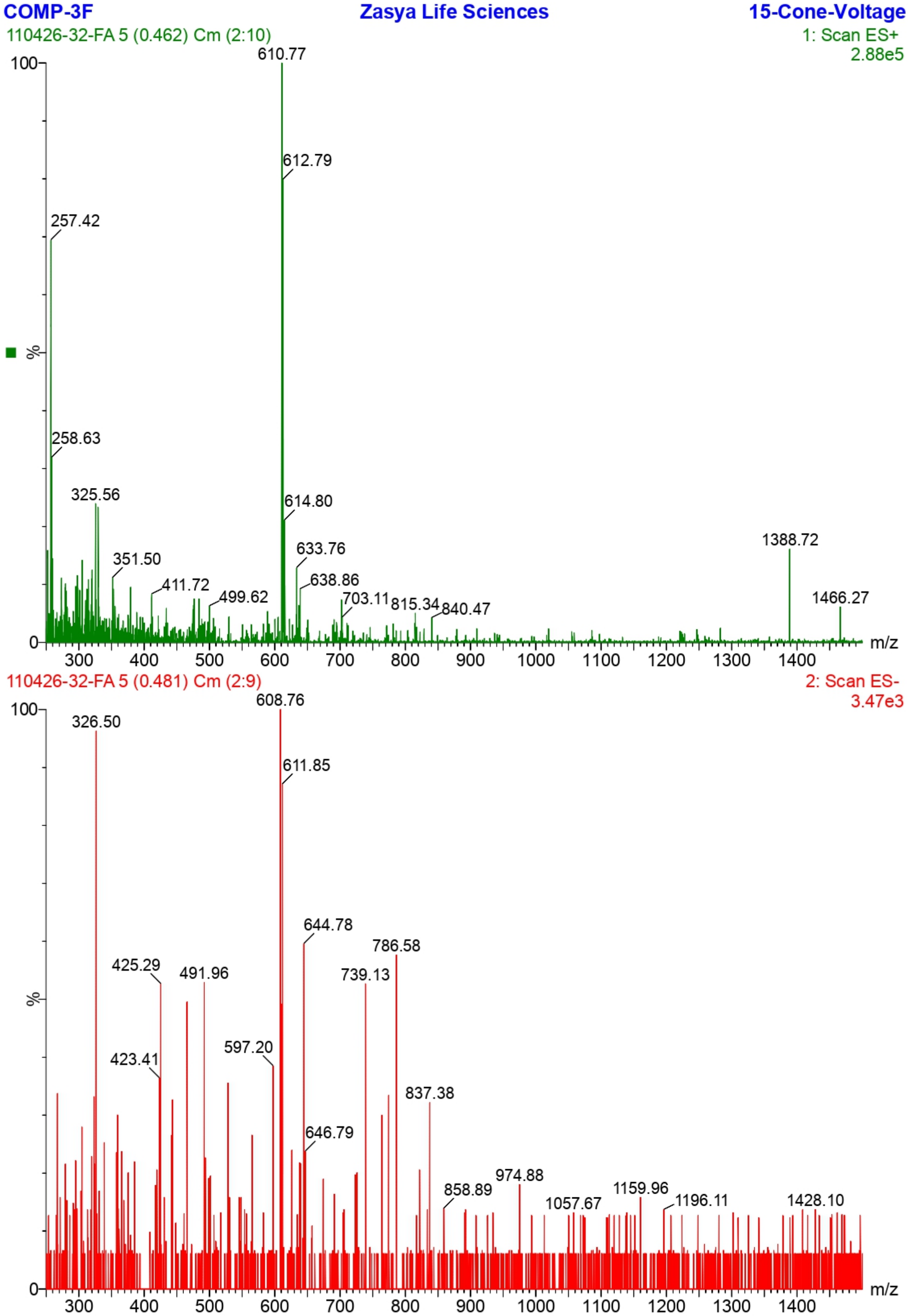
COMP-3F
Zasya Life Sciences
15-Cone-Voltage
110426-32-FA 5 (0.462) Cm (2:10)
1: Scan ES+
2.88e5
610.77
612.79
257.42
258.63
325.56
614.80
351.50
633.76
1388.72
638.86
411.72
499.62
703.11
815.34
840.47
1466.27
%
m/z
110426-32-FA 5 (0.481) Cm (2:9)
2: Scan ES-
3.47e3
608.76
326.50
611.85
644.78
786.58
425.29
491.96
739.13
597.20
423.41
837.38
646.79
974.88
858.89
1159.96
1057.67
1196.11
1428.10
%
m/z

Compound 4g

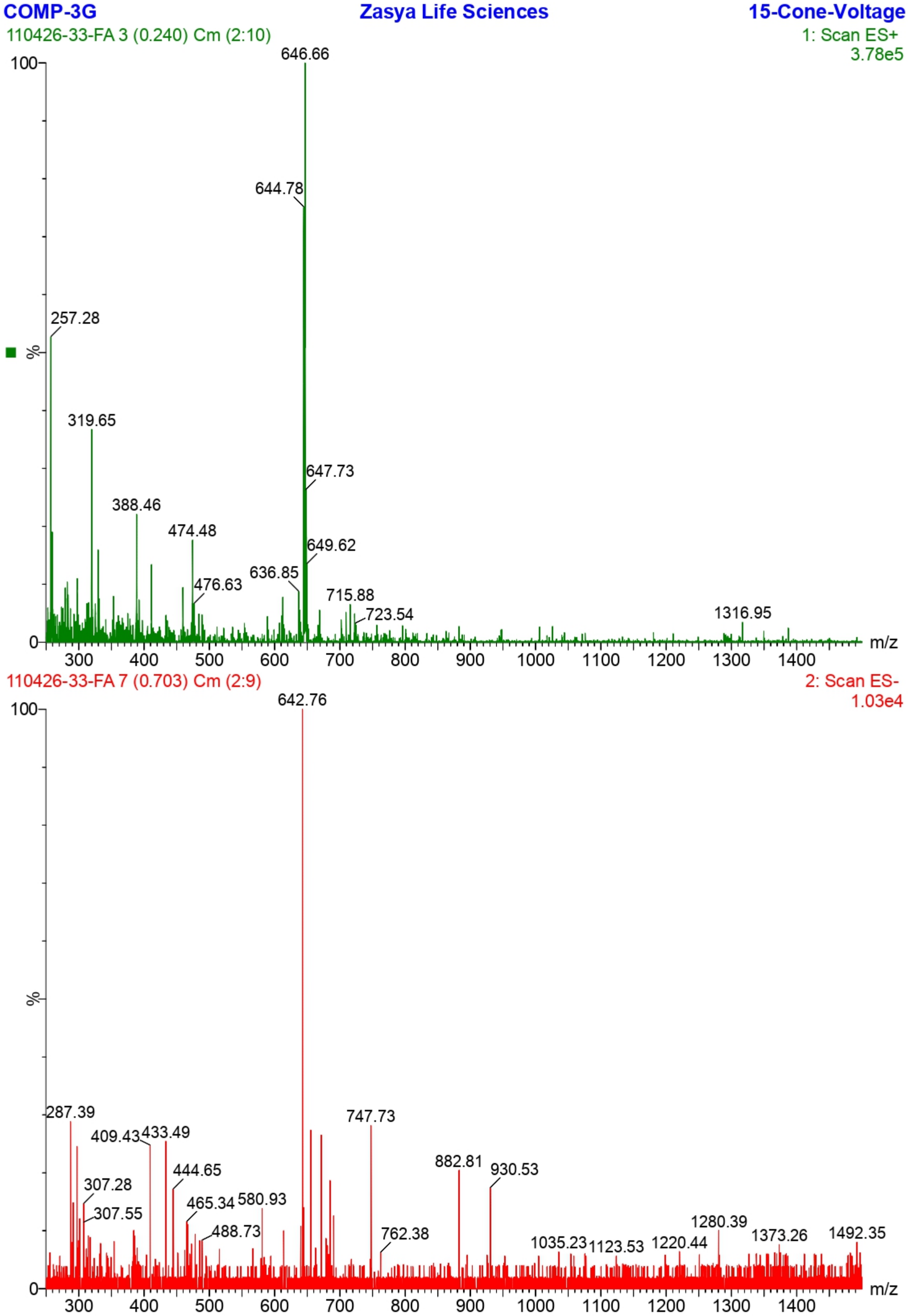

COMP-3G
Zasya Life Sciences
15-Cone-Voltage
110426-33-FA 3 (0.240) Cm (2:10)
1: Scan ES+
3.78e5
646.66
644.78
257.28
319.65
647.73
388.46
474.48
649.62
636.85
476.63
715.88
723.54
1316.95
m/z
%
110426-33-FA 7 (0.703) Cm (2:9)
2: Scan ES-
1.03e4
642.76
287.39
747.73
409.43
433.49
444.65
882.81
930.53
307.28
307.55
465.34
580.93
488.73
762.38
1280.39
1035.23
1123.53
1220.44
1373.26
1492.35

Compound 4h

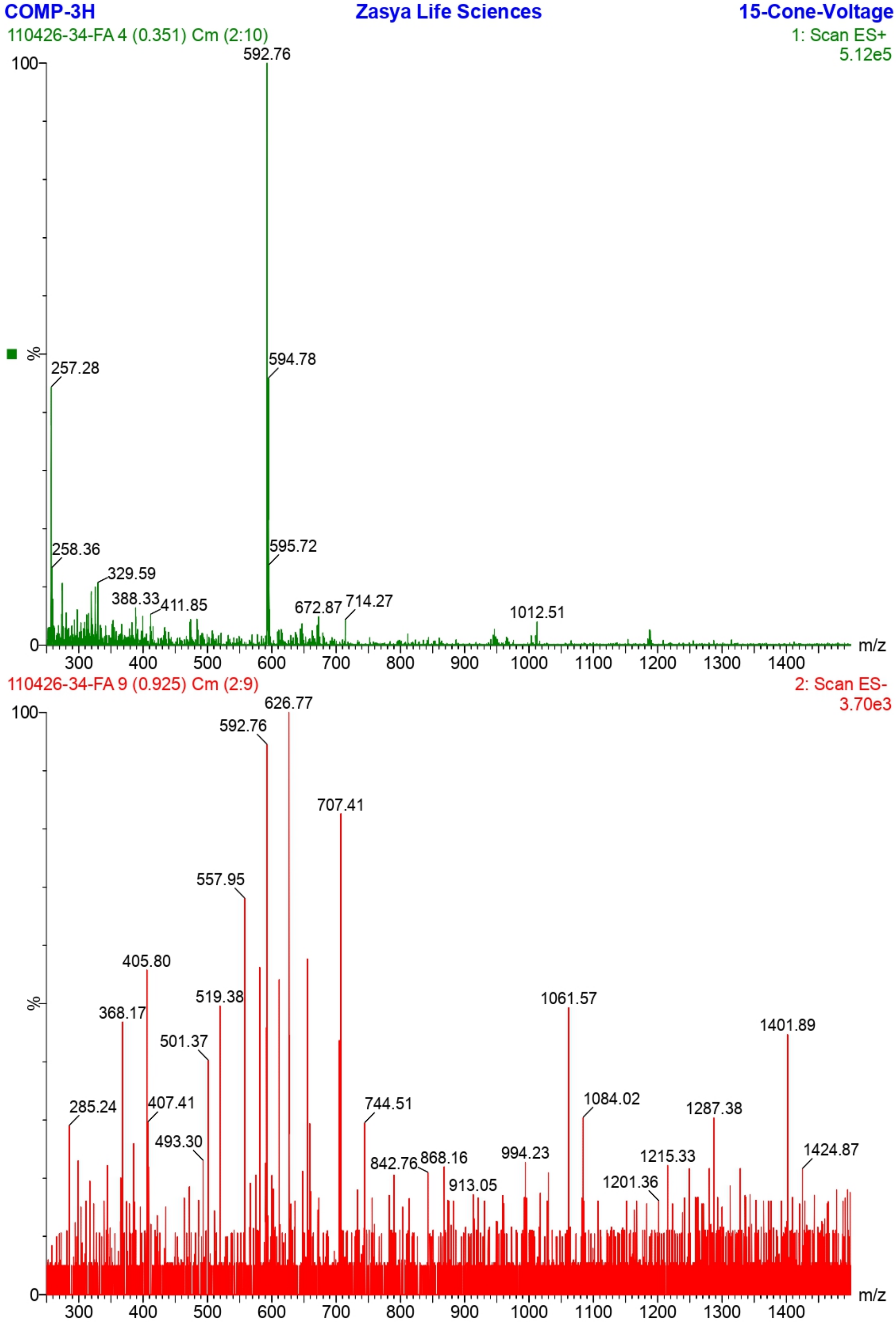
COMP-3H
Zasya Life Sciences
15-Cone-Voltage
110426-34-FA 4 (0.351) Cm (2:10)
1: Scan ES+
5.12e5
592.76
594.78
257.28
258.36
595.72
329.59
388.33
411.85
672.87
714.27
1012.51
%
m/z
110426-34-FA 9 (0.925) Cm (2:9)
2: Scan ES-
3.70e3
626.77
592.76
707.41
557.95
405.80
519.38
368.17
501.37
1061.57
1401.89
285.24
407.41
744.51
1084.02
1287.38
493.30
842.76
868.16
994.23
913.05
1215.33
1201.36
1424.87
%
m/z

Supplementary material 5. Molecular docking studies.

Compound	Binding Energy (kcal/mol)	H-Bonds (backbone)	Halogen Bonds	π-Interactions	Key Hydrophobic Contacts
**4a**	-7.549	Val420	—	—	Leu365, Ala366, Cys368, Ile416, Val418, Ile559, Thr560, Val561, Cys513, Val514, Leu515, Val604, Val606, Ala607
**4b**	-8.087	Val420	—	—	Leu365, Ala366, Cys368, Ile416, Val418, Val512, Cys513, Val514, Leu515, Val467, Leu468, Ile559, Thr560, Val561, Val604/606
**4c**	-7.891	—	—	—	Leu365, Ala366, Gly367/368, Val369/370, Val463–467, Ile416, Val418, Ile421, Val512–514, Val604 (purely hydrophobic pocket; no polar contacts marked)
**4d**	-8.325	—	Arg326 → Cl	Hie516 ↔ fluorophenyl ring (π–π)	Leu365, Cys368, Val369, Ile559, Thr560, Val561, Val604/606/608, Ala607, Leu610, Cys513, Val512/514, Leu515
**4e**	-8.038	Val467	Arg326 → Cl	—	Ile416, Val418, Val463/465, Ala466, Leu365, Ala366, Val514, Leu515, Ala556, Leu557, Ile559, Val561, Val604/606/608
**4f**	-8.603	Val420	—	—	Leu365, Ala366, Cys368, Ile416, Val418, Cys513, Val514, Leu515, Ile559, Thr560, Val561, Val604/606

**4g**	-8.126	Arg326 → ring N; Val514 → HN	Val418 → Cl; Val465 → Cl; Val606 → Cl; Ile559 → Cl; Asn517 → Cl (5 total)	—	Ile416, Leu365, Ala366, Val463/467/468, Cys513, Leu515, Val604/608/610
**4h**	-7.325	Val420	—	—	Leu365, Ala366, Cys368, Ile416, Val418, Val514, Leu515, Ile559, Thr560, Val561, Val604–607
**Ascorbic acid**	-7.541	Val418; Val606; Gly367; Val512; Val465 (5 total)	—	—	Ile559, Leu557, Gly558, Val604, Gly605, Ala510, Gly511, Cys513, Ala366

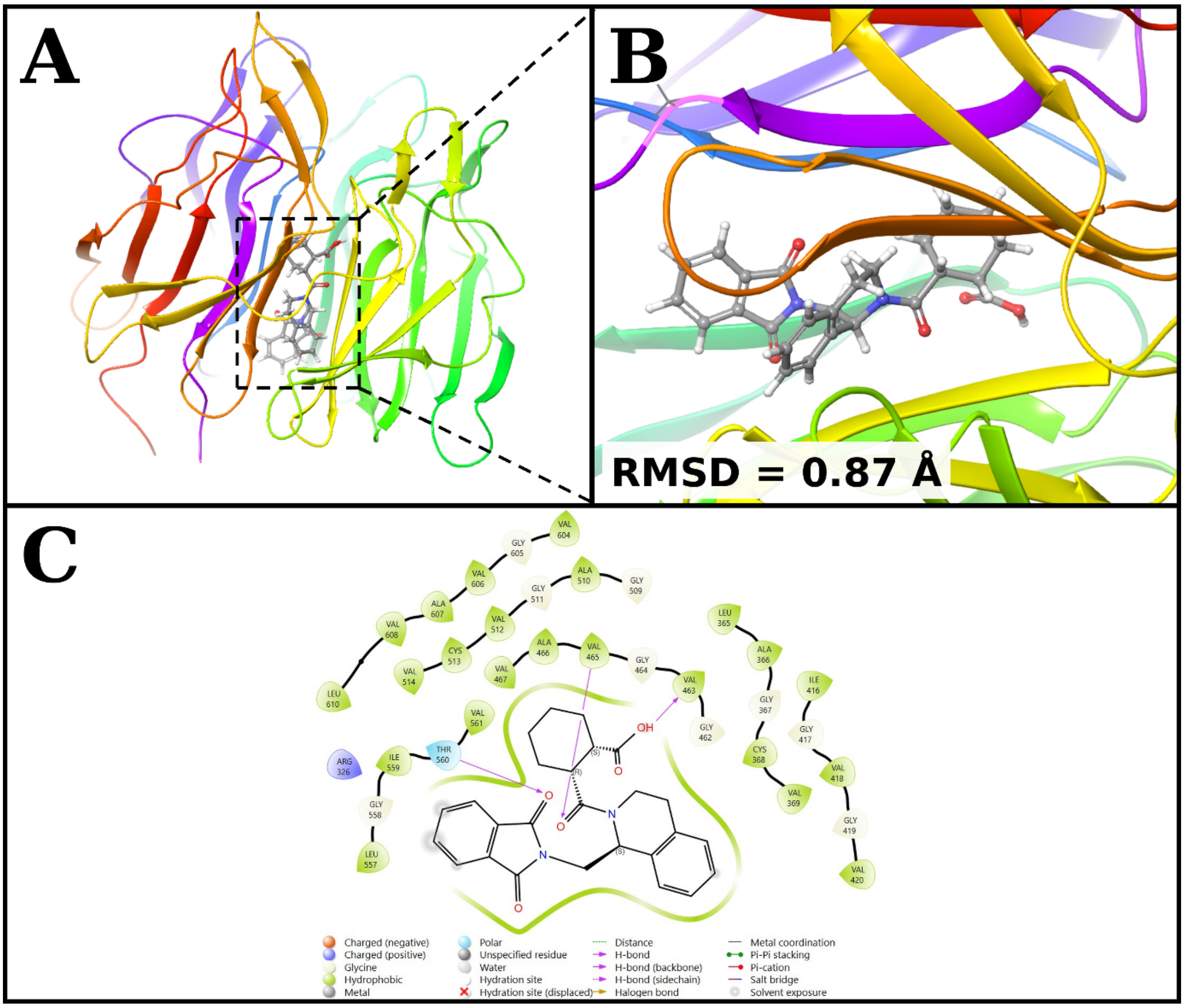

Fig. S1. Validation of the docking protocol by re-docking the co-crystallized ligand into the Kelch domain of Keap1 (PDB: 4L7B). (A) Overall view of the re-docked ligand within the Kelch β-propeller. (B) Close-up of the binding pocket; RMSD between the re-docked and crystallographic poses = 0.87 Å. (C) Two-dimensional interaction diagram of the re-docked ligand.

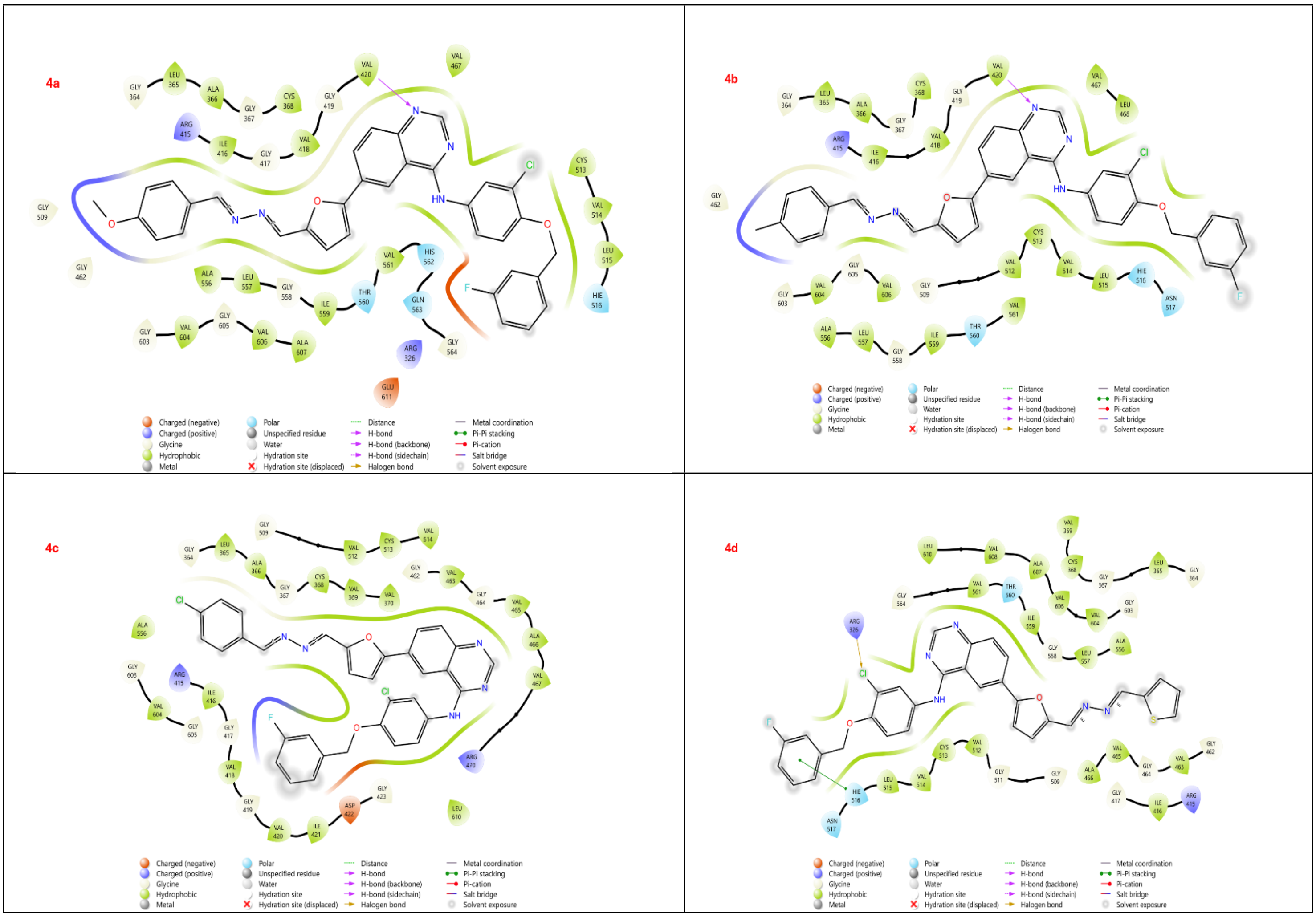
4a
4b
4c
4d
Charged (negative)
Charged (positive)
Glycine
Hydrophobic
Metal
Polar
Unspecified residue
Water
Hydration site
Hydration site (displaced)
Distance
H-bond
H-bond (backbone)
H-bond (sidechain)
Halogen bond
Metal coordination
Pi-Pi stacking
Pi-cation
Salt bridge
Solvent exposure

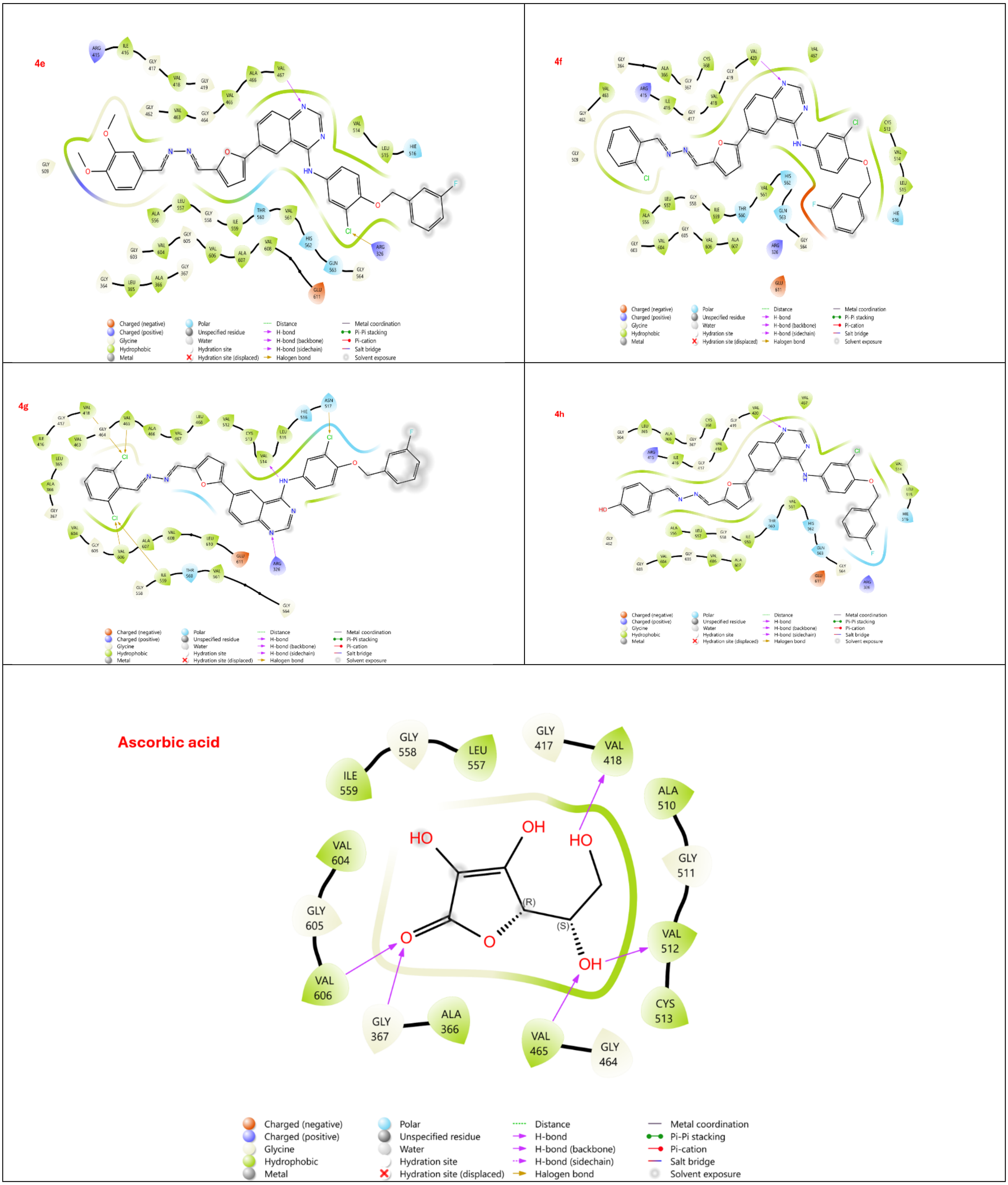
4e
4f
4g
4h
Ascorbic acid
GLY 558
LEU 557
GLY 417
VAL 418
ILE 559
ALA 510
VAL 604
GLY 511
GLY 605
VAL 512
VAL 606
CYS 513
GLY 367
ALA 366
VAL 465
GLY 464
HO
OH
HO
O
O
OH
(R)
(S)
Charged (negative)
Charged (positive)
Glycine
Hydrophobic
Metal
Polar
Unspecified residue
Water
Hydration site
Hydration site (displaced)
Distance
H-bond
H-bond (backbone)
H-bond (sidechain)
Halogen bond
Metal coordination
Pi-Pi stacking
Pi-cation
Salt bridge
Solvent exposure

Fig. S2. Two-dimensional ligand–interaction diagrams of the binding pose within the Kelch β-propeller domain of Keap1 (right) for the representative compounds **4a-h** and ascorbic acid (PDB: 4L7B).

Supplementary material 6. Antioxidant study graph.

Antioxidant Graph

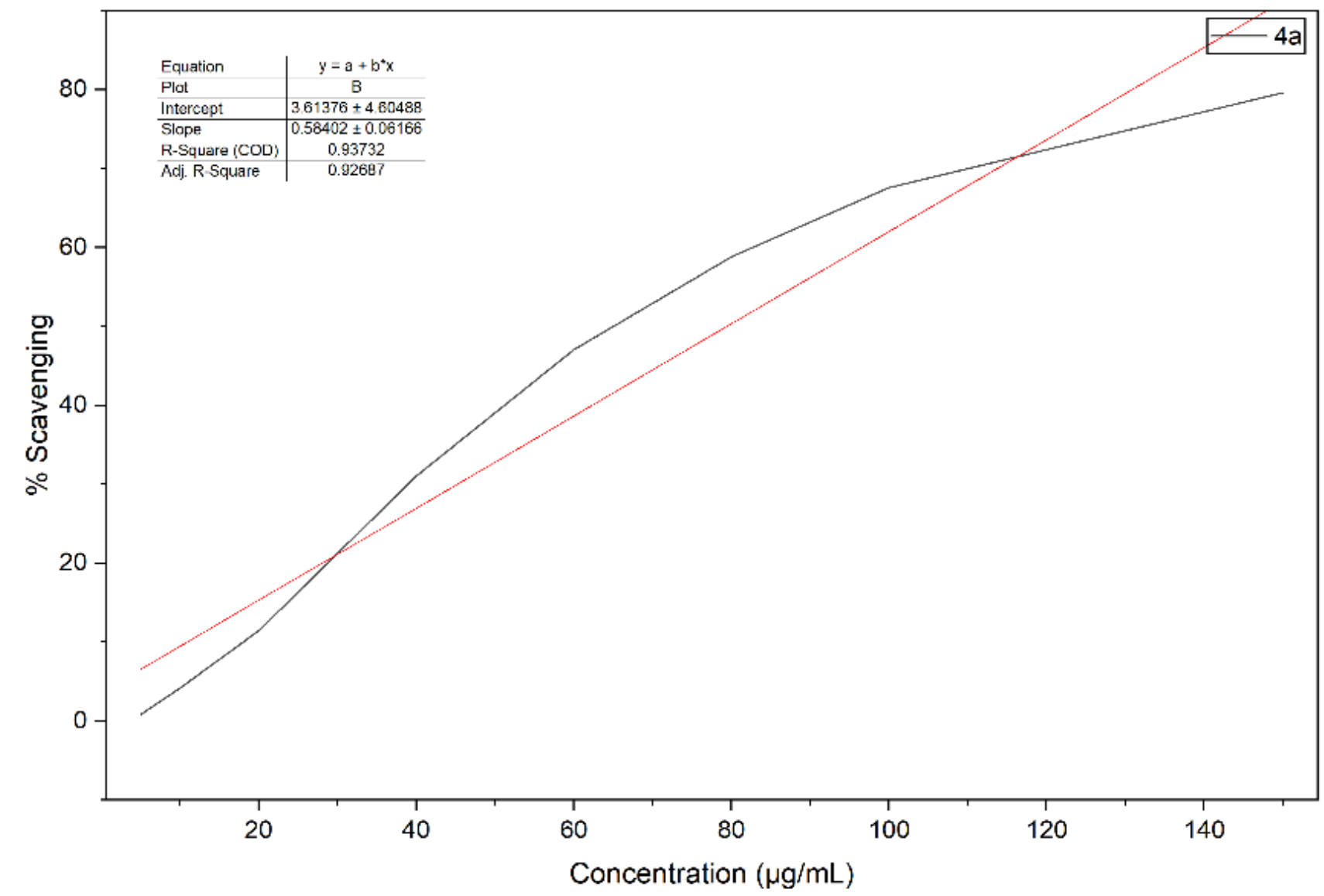

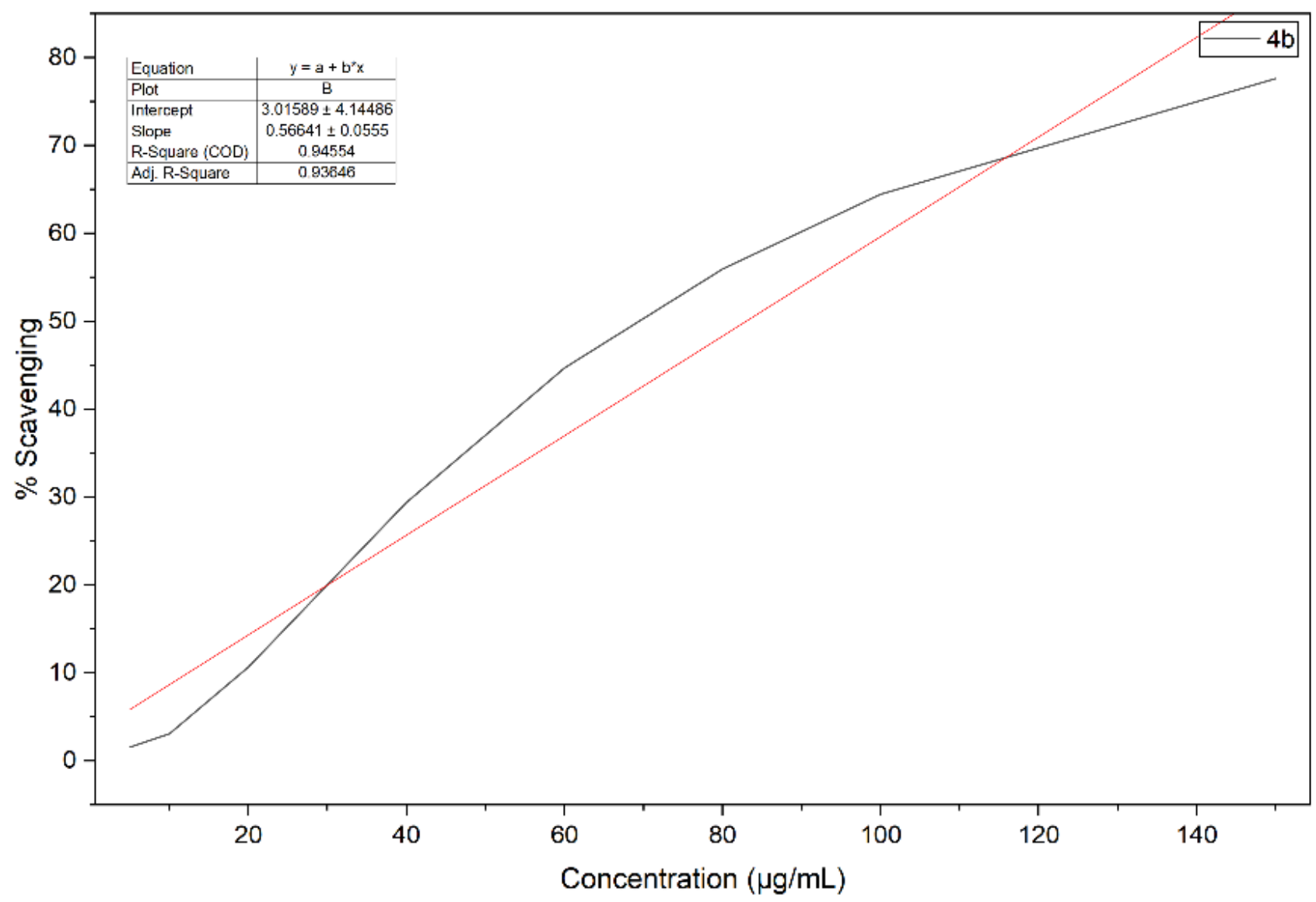

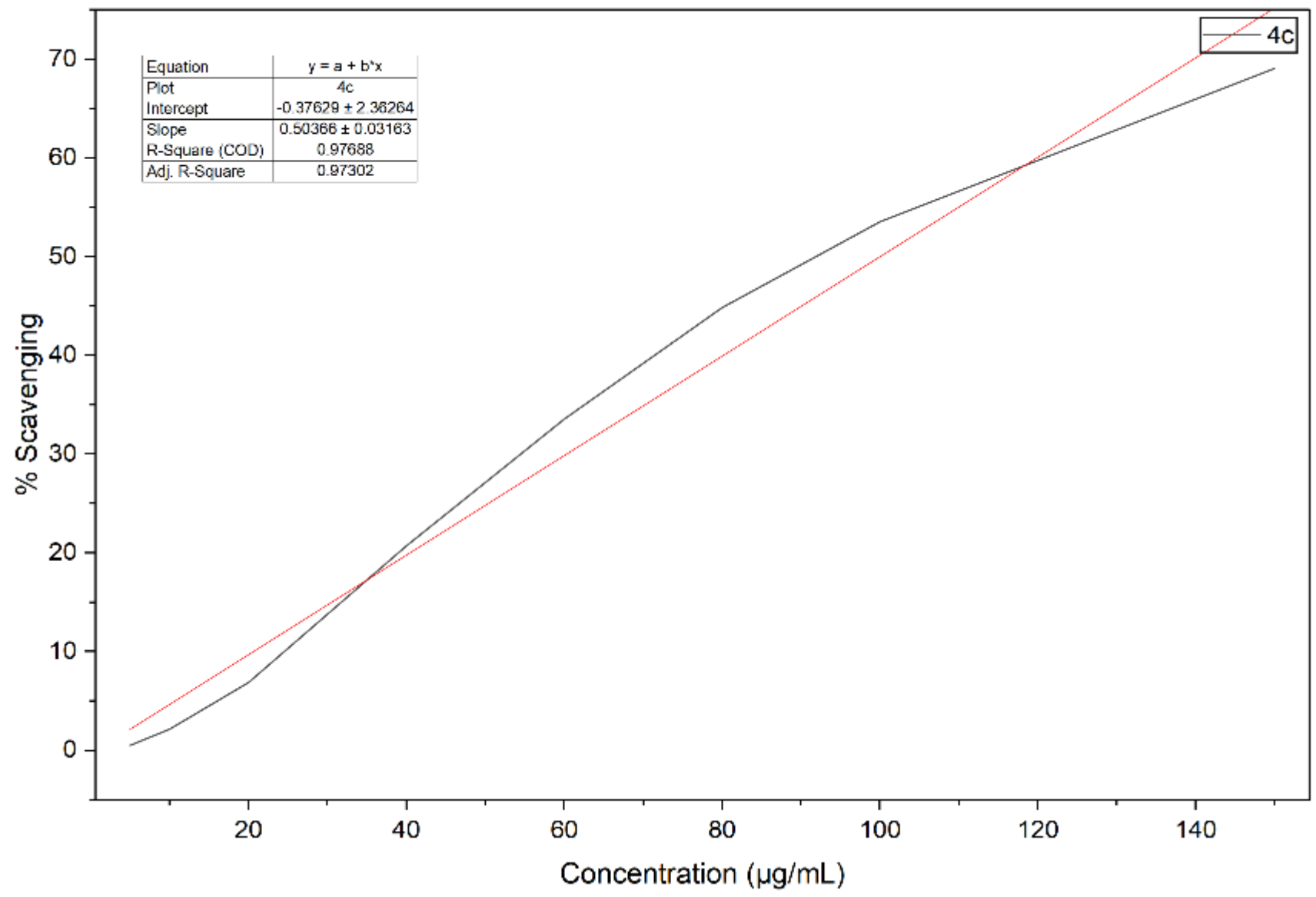
Equation | y = a + b*x
Plot | 4c
Intercept | -0.37629 ± 2.36264
Slope | 0.50366 ± 0.03163
R-Square (COD) | 0.97688
Adj. R-Square | 0.97302
4c
% Scavenging
0
10
20
30
40
50
60
70
20
40
60
80
100
120
140
Concentration (µg/mL)

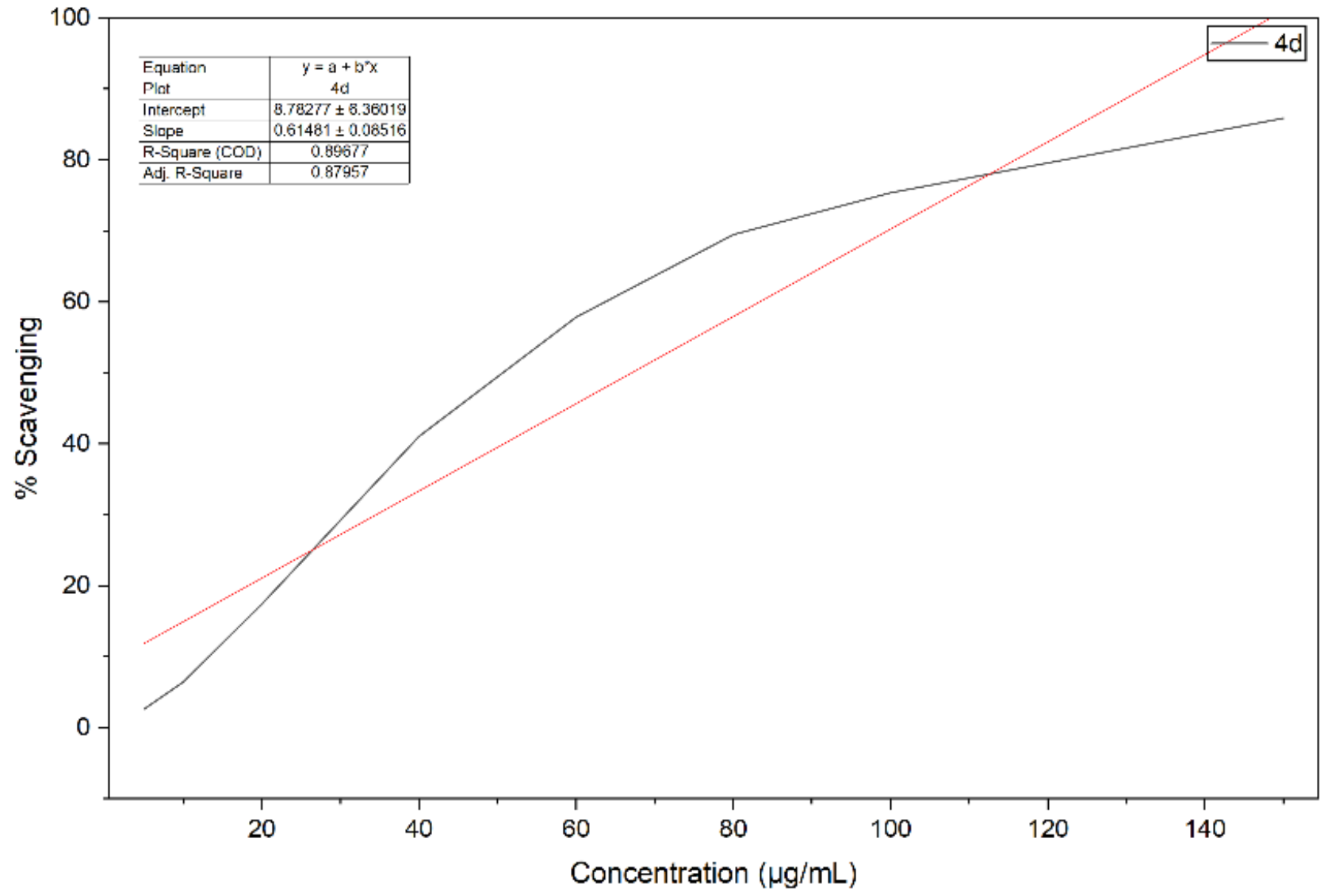
Equation | y = a + b*x
Plot | 4d
Intercept | 8.78277 ± 6.36019
Slope | 0.61481 ± 0.08516
R-Square (COD) | 0.89677
Adj. R-Square | 0.87957
4d
% Scavenging
0
20
40
60
80
100
20
40
60
80
100
120
140
Concentration (µg/mL)

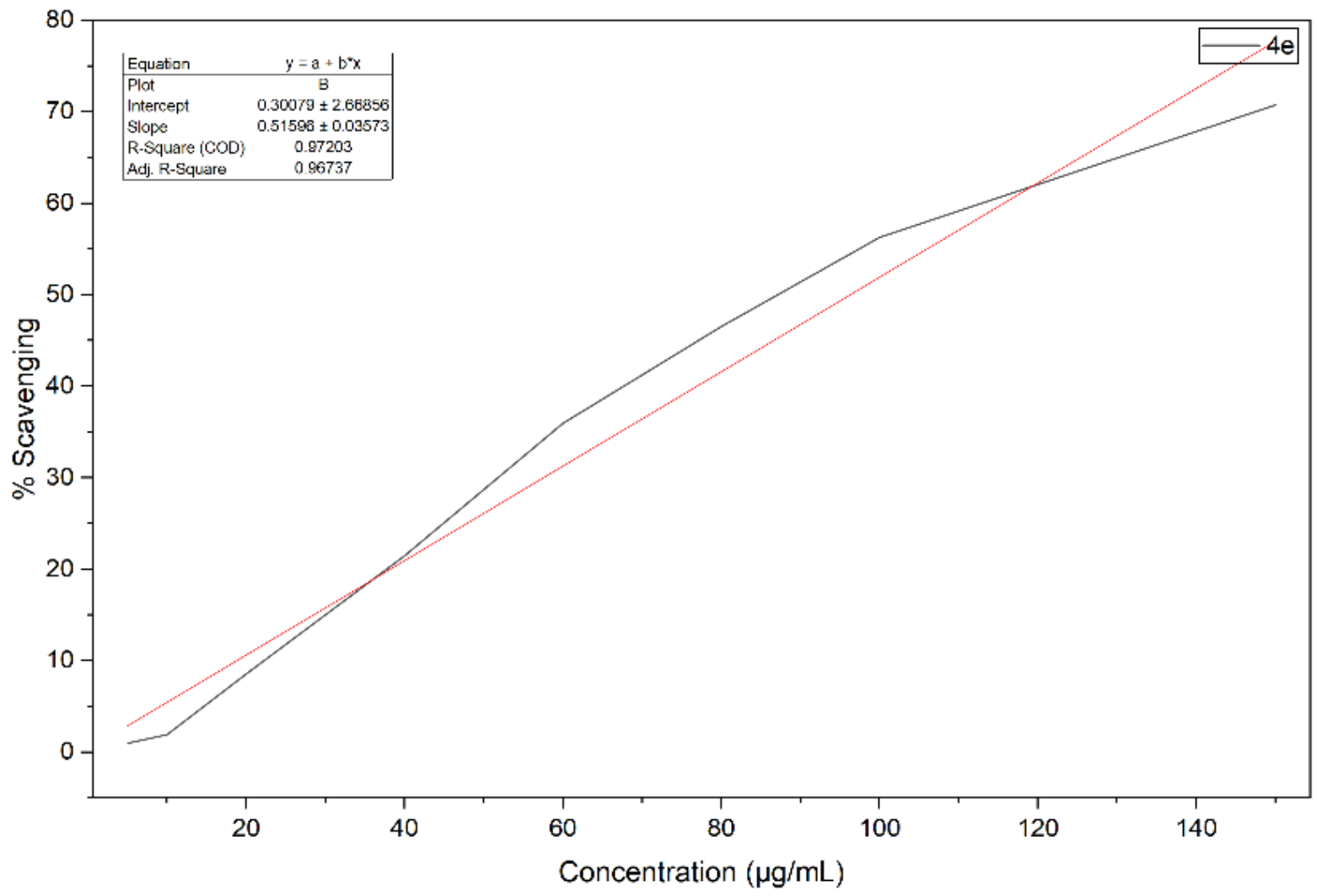
Equation | y = a + b*x
Plot | B
Intercept | 0.30079 ± 2.66856
Slope | 0.51596 ± 0.03573
R-Square (COD) | 0.97203
Adj. R-Square | 0.96737
4e
% Scavenging
0
10
20
30
40
50
60
70
80
20
40
60
80
100
120
140
Concentration (µg/mL)

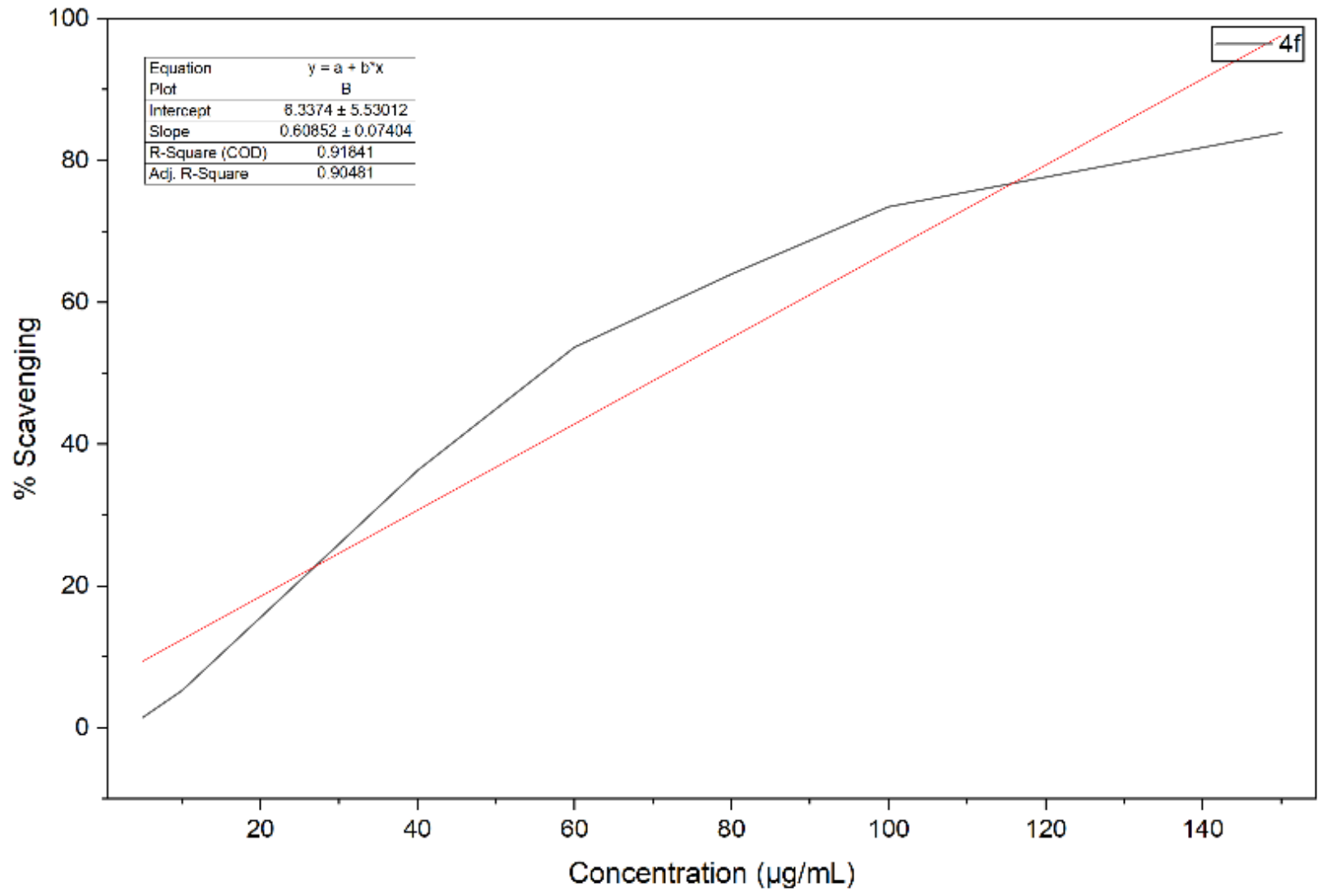

Equation y = a + b*x
Plot B
Intercept 8.3374 ± 5.53012
Slope 0.60852 ± 0.07404
R-Square (COD) 0.91841
Adj. R-Square 0.90481
4f
% Scavenging
Concentration (µg/mL)

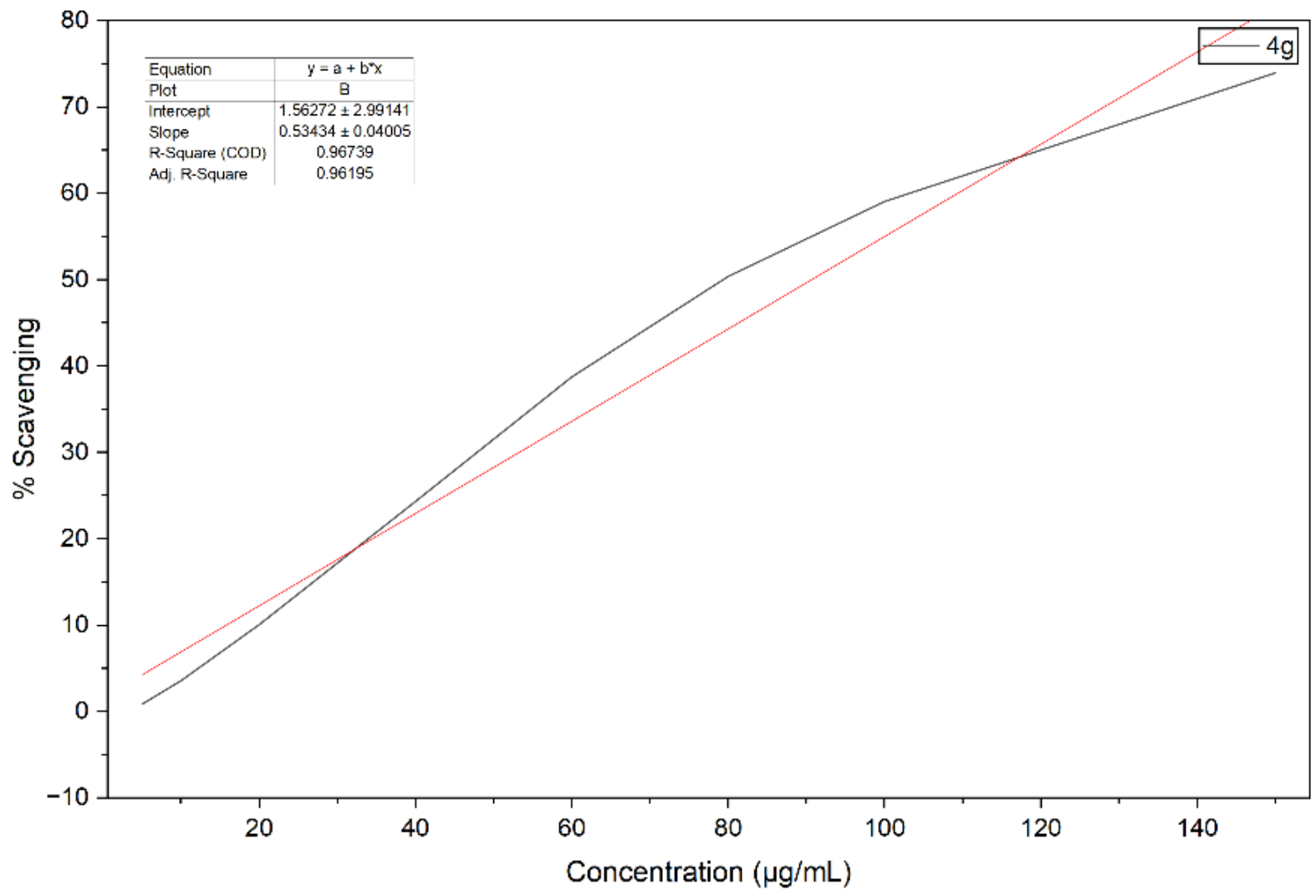

Equation y = a + b*x
Plot B
Intercept 1.56272 ± 2.99141
Slope 0.53434 ± 0.04005
R-Square (COD) 0.96739
Adj. R-Square 0.96195
4g
% Scavenging
Concentration (µg/mL)

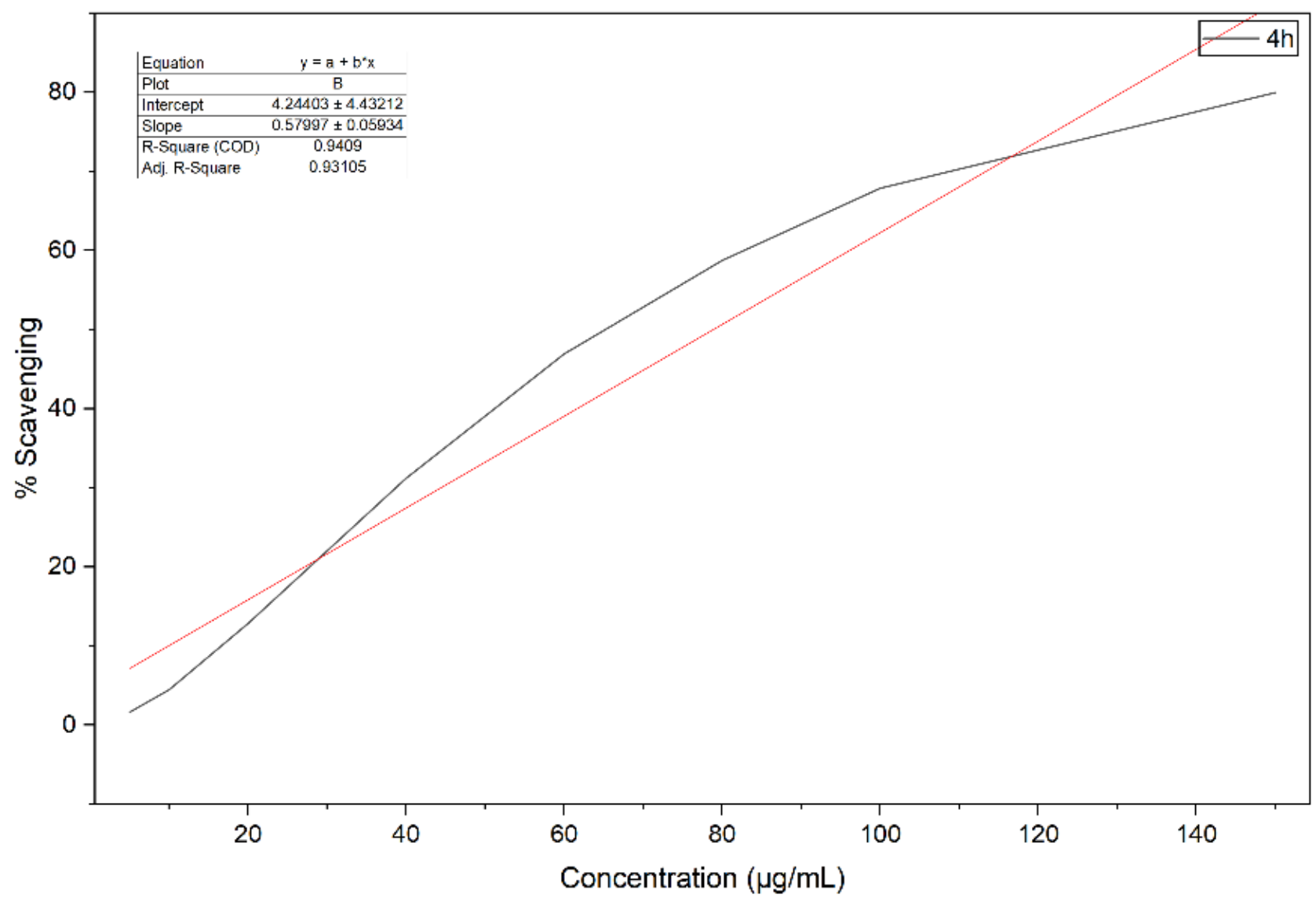
Equation y = a + b*x
Plot B
Intercept 4.24403 ± 4.43212
Slope 0.57997 ± 0.05934
R-Square (COD) 0.9409
Adj. R-Square 0.93105
4h
% Scavenging
Concentration (μg/mL)

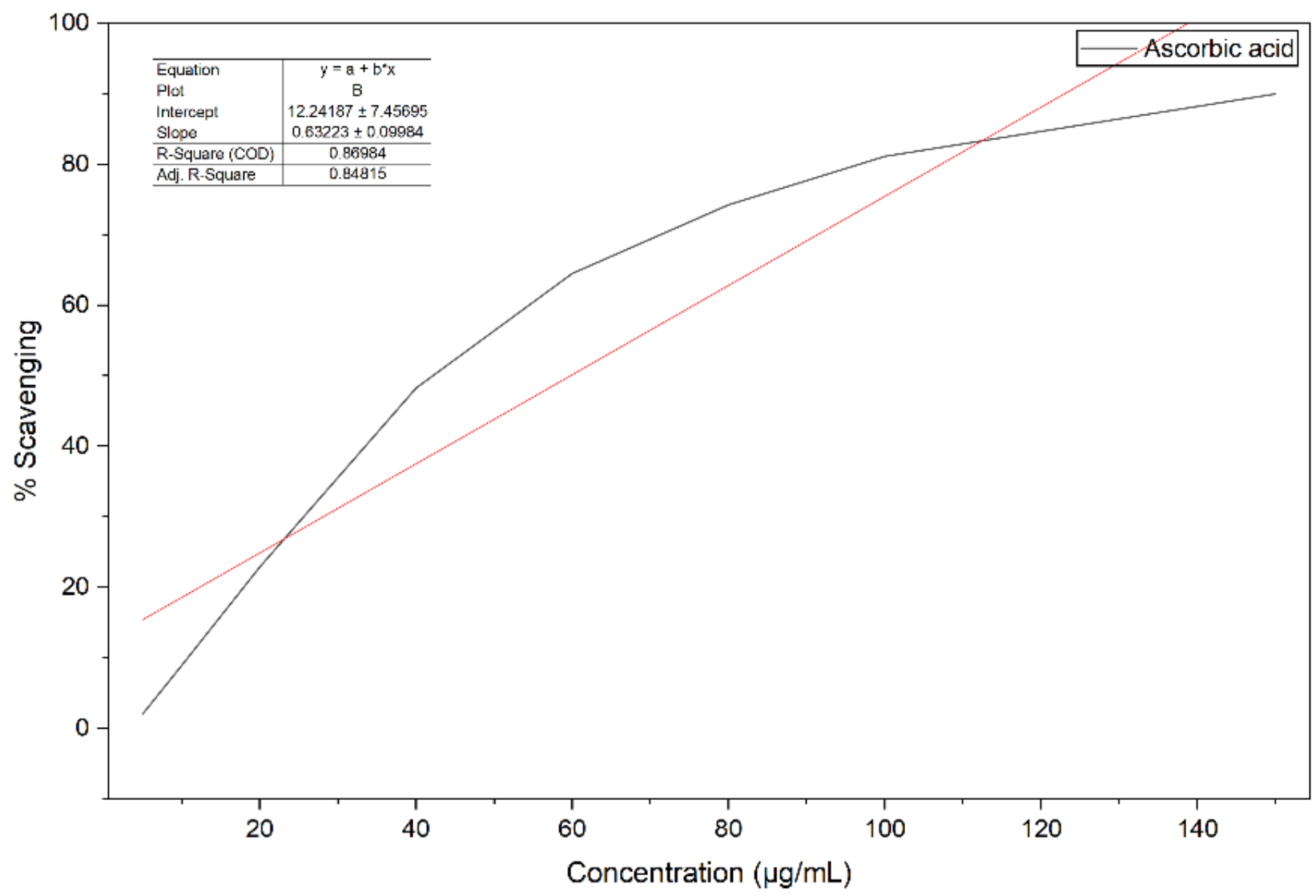
Equation y = a + b*x
Plot B
Intercept 12.24187 ± 7.45695
Slope 0.63223 ± 0.09984
R-Square (COD) 0.86984
Adj. R-Square 0.84815
Ascorbic acid
% Scavenging
Concentration (μg/mL)
